# Supplementary material for: The cholesterol uptake regulator PCSK9 promotes and is a therapeutic target in APC/KRAS-mutant colorectal cancer
Source: Nat Commun. 2022 Jul 8;13:3971. doi: 10.1038/s41467-022-31663-z (PMC9270407; doi:10.1038/s41467-022-31663-z)
Supplement: Supplementary file 1 — Supplementary Information [file 41467_2022_31663_MOESM1_ESM.pdf]

Supplementary Information for The cholesterol uptake regulator PCSK9 promotes and is a therapeutic target in APC/KRAS-mutant colorectal cancer

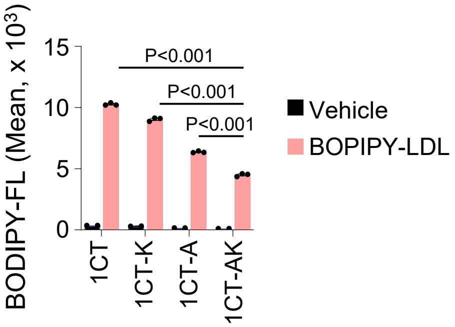

Supplementary Figure S1. Quantitative analysis of intracellular fluorescence after incubation with BODIPY-LDL (10μg/ml, 4h) by flow cytometry (n=3). Data shown are means of biological replicates; ± S.E.M. Two-tailed Student's t-test.

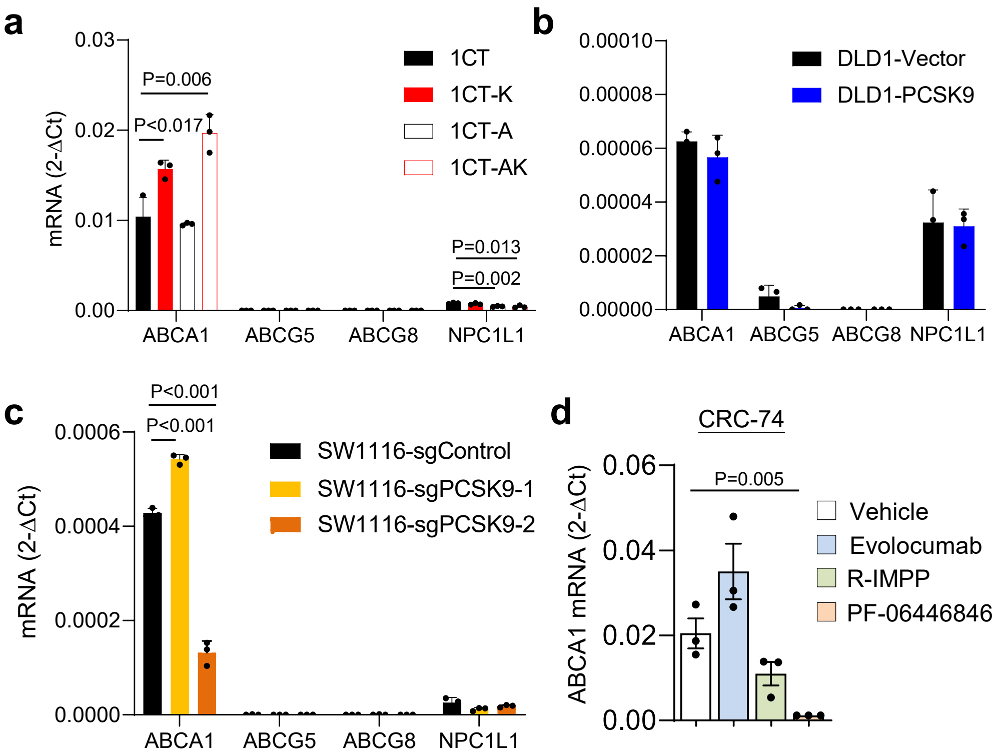

Supplementary Figure S2. qPCR of ABCA1, ABCG, ABCG8, and NPCL11 expression in **a** 1CT isogenic cells (n=3), **b** DLD1 cells with PCSK9overexpression (n=3), **c** SW1116 cells with PCSK9 knockout (n=3) and **d** CRC-74 organoids (n=3). Data shown are means of biological replicates; ± S.E.M (**a**, **b**, **c**, **d**). Two-tailed Student's t-test (**a**, **b**, **c**, **d**).

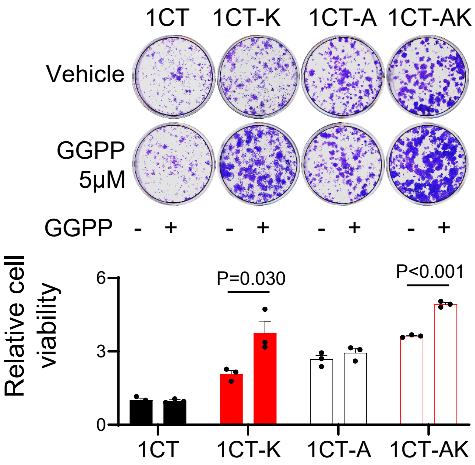

Supplementary Figure S3. Colony formation assays showed that GGPP promoted the cell growth of 1CT-K and 1CT-AK cells, but not in 1CT and 1CT-A cells (14 days, n=3). Data shown are means of biological replicates; ± S.E.M. Two-tailed Student's t-test.

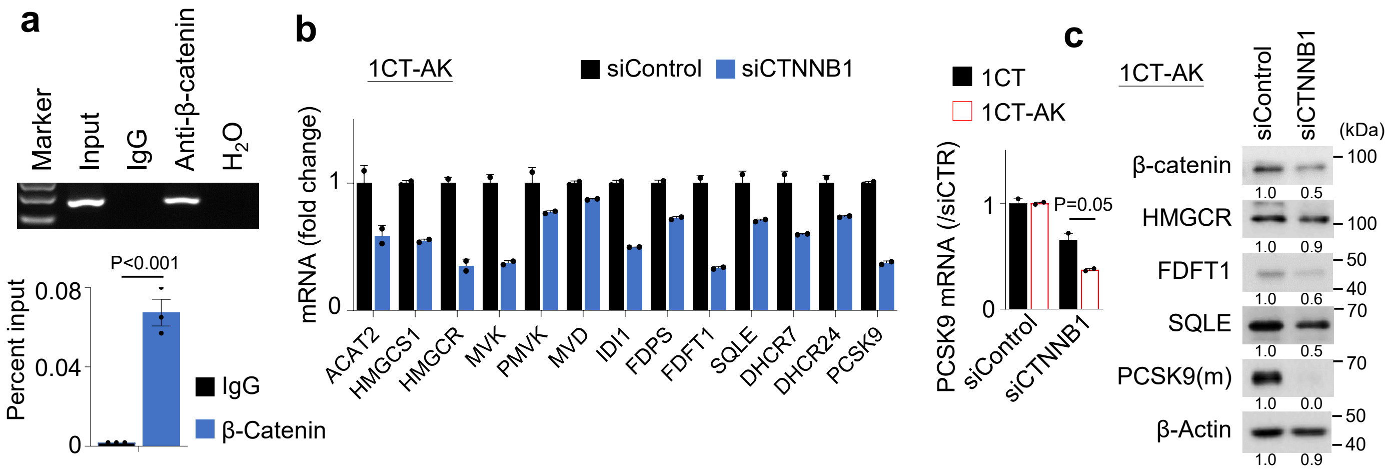

Supplementary Figure S4. β-catenin regulates PCSK9. **a** Chromatin immunoprecipitation-PCR (ChIP-PCR) and qPCR analysis of the binding of β-catenin to PCSK9 promoter (n=3). **b** β-catenin knockdown (72h) inhibited mRNA expression of PCSK9 and cholesterol biosynthesis genes in 1CT-AK cells (left) (n=2), with a more pronounced effect on PCSK9 mRNA in 1CT-AK cells compared to 1CT cells (right). Data are presented as means ± S.E.M. t-test (two-sided) **c** Western blot of effects of β-catenin knockdown in 1CT-AK cells. Data shown are means of biological replicates; ± S.E.M. (**a**, **b**). Two-tailed Student's t-test (**a**, **b**).

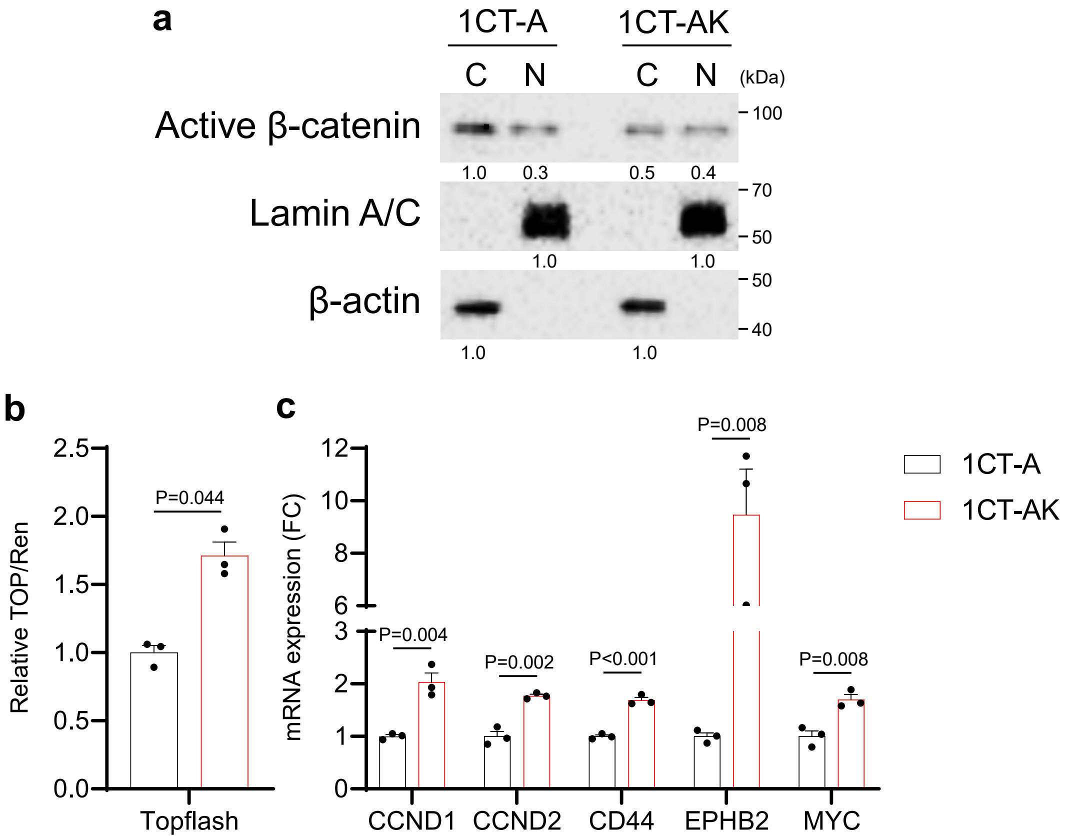

Supplementary Figure S5. Mutant KRAS promotes WNT/β-catenin signaling. **a** The localization of active β-catenin, **b** TOPflash activity (n=3), and **c** mRNA expression of β-catenin targets in 1CT-A and 1CT-AK cells (n=3). Data shown are means of biological replicates; ± S.E.M. (**b**, **c**). Two-tailed Student's t-test (**b**, **c**).

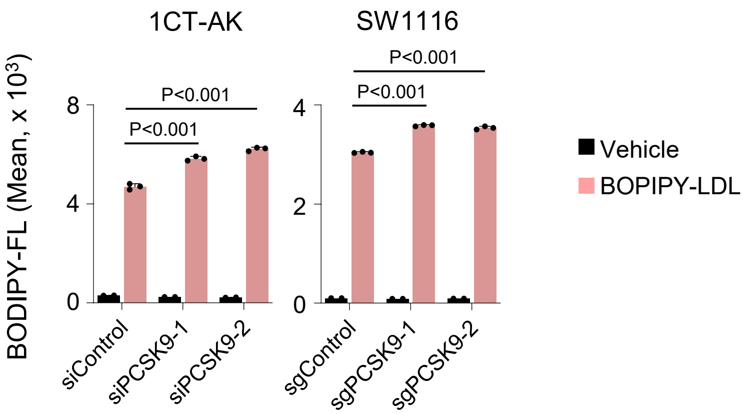

Supplementary Figure S6. Knockdown or knockout of PCSK9 induced the uptake of BODIPY-FL LDL (10μg/mL for 4h), as determined by flow cytometry (n=3). Data shown are means of biological replicates; ± S.E.M. Two-tailed Student's t-test.

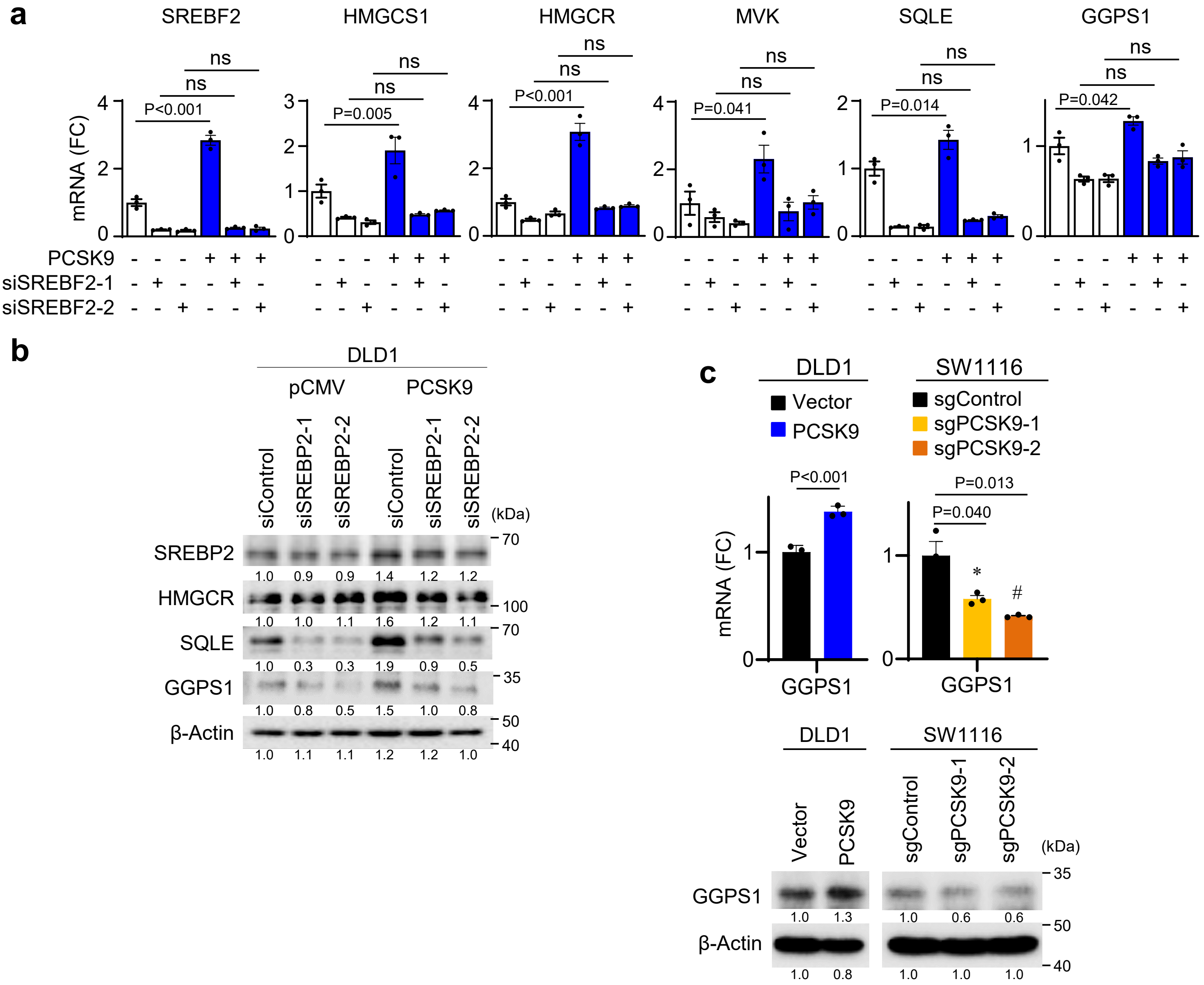

Supplementary Figure S7. a SREBP2 knockdown abrogated PCSK9 overexpression-mediated expression of cholesterol biosynthesis genes (n=3) and **b** validation by western blot. **c** PCSK9 knockout reduced GGPS1, whereas PCSK9 overexpression exerted an opposite effect (n=3). Data shown are means of biological replicates; ±S.E.M. (**a**, **c**) Two-tailed one way ANOVA (**a**). Two-tailed Student's t-test (**c**).

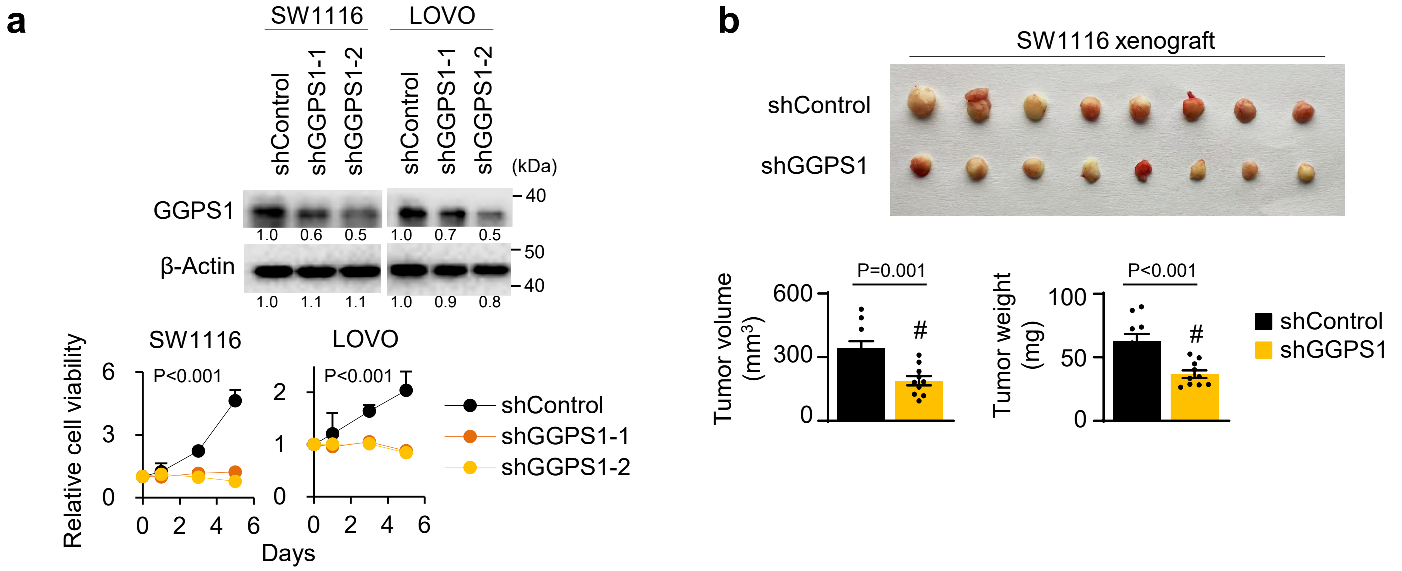

Supplementary Figure S8. GGPS1 knockdown inhibited **a** cell viability in vitro (n=6) and **b** growth of SW1116 xenografts in vivo (day 12, n=1 experiment, n=12 mice per group). Data shown are means of biological replicates; ± S.E.M. (**a**, **b**). Two-tailed Student's t-test (**a**, **b**).

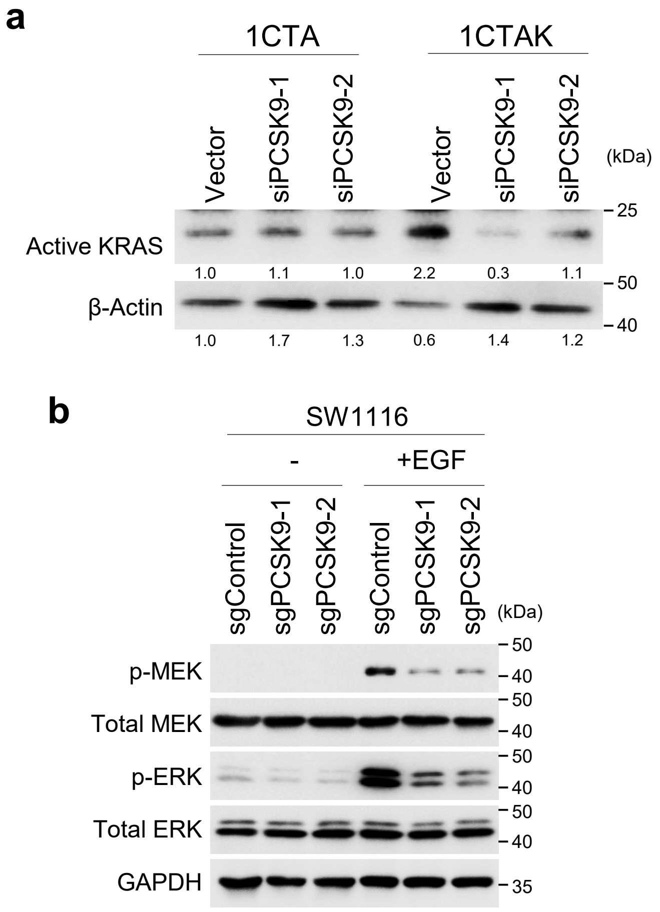

Supplementary Figure S9. a Active KRAS in isogenic 1CT cells with or without the knockdown of PCSK9. **b** SW1116-sgControl and SW1116-sgPCSK9 cells were serum-starved overnight, and then stimulated with EGF (10ng/mL) for 30min. sgPCSK9 suppressed EGF-mediated up-regulation of p-MEK and p-ERK.

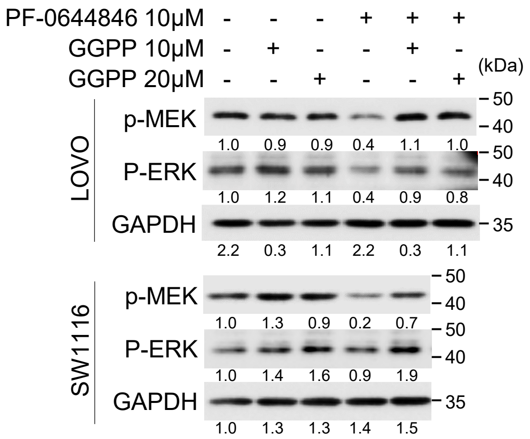

Supplementary Figure S10. GGPP restored p-MEK and p-ERK expression in LOVO and SW1116 cells treated with PF-0644846.

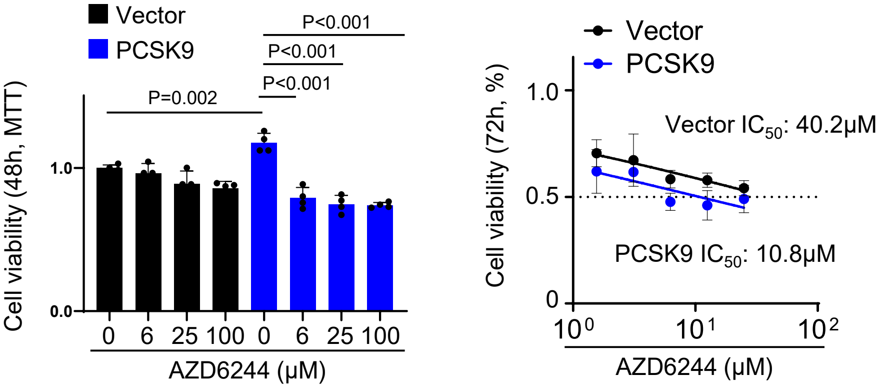

Supplementary Figure S11. MEK1/2 inhibitor AZD6244 abrogated the growth promoting effect of PCSK9 overexpression in DLD1 cells (n=4). Data shown are means of biological replicates; ± S.E.M. Two-tailed Student's t-test.

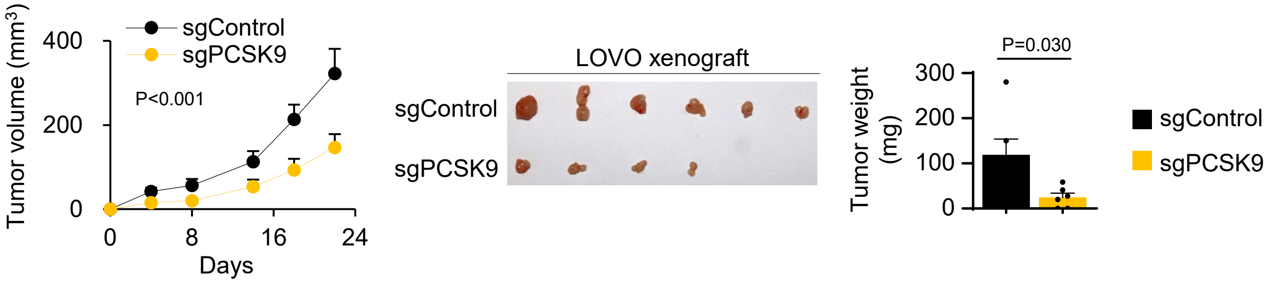

Supplementary Figure S12. PCSK9 knockout inhibited growth of LOVO xenografts in nude mice (n=1 experiment, n=6 mice per group). Data shown are means of biological replicates; ±S.E.M. Two-tailed Student's t-test. Two-tailed two-way ANOVA (for growth curve).

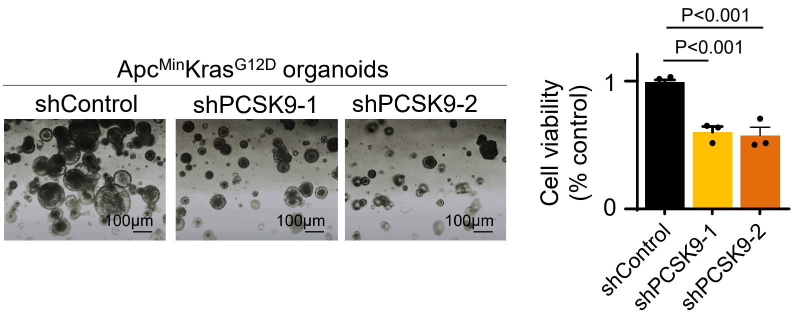

Supplementary Figure S13. Colon tumor organoids isolated from $Apc^{Min/+}Kras^{G12D/+}$Villin-Cre mice were transduced with lentiviral Pcsk9-shRNA and cell viability was assessed (n=3). Data shown are means of biological replicates; ± S.E.M. Two-tailed Student's t-test.

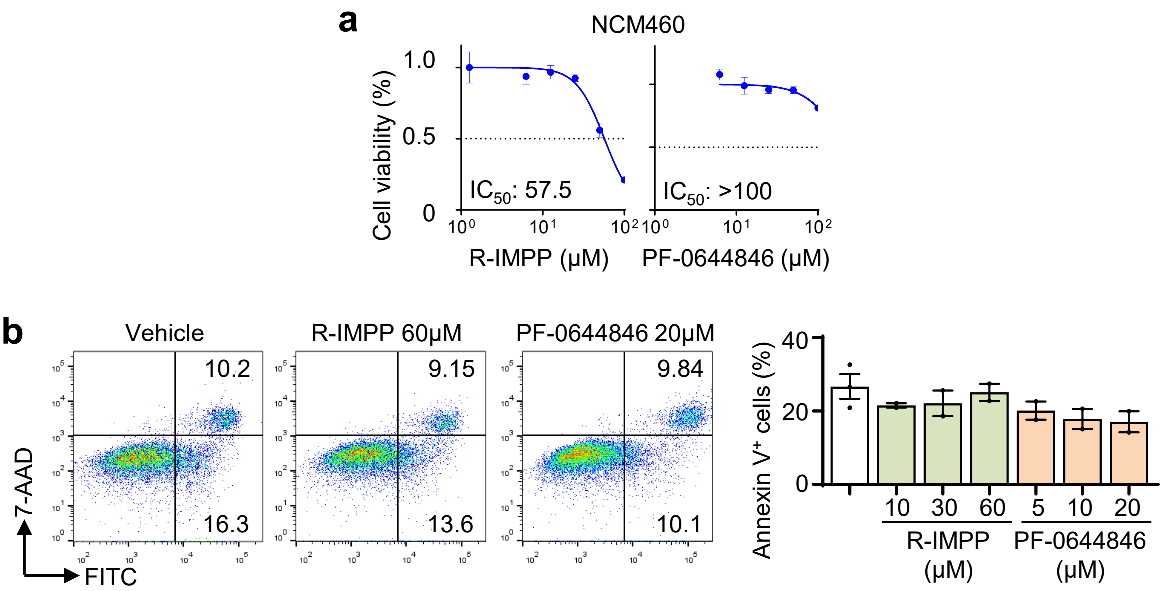

Supplementary Figure S14. Effect of PCSK9 inhibitors on NCM460 normal colon cell **a** growth and **b** apoptosis. Cell were treated for 72h and analyzed by MTT (n=3) or flow cytometry (n=2). Data shown are means of biological replicates; ± S.E.M. (**a**, **b**). Two-tailed Student's t-test (**a**, **b**).

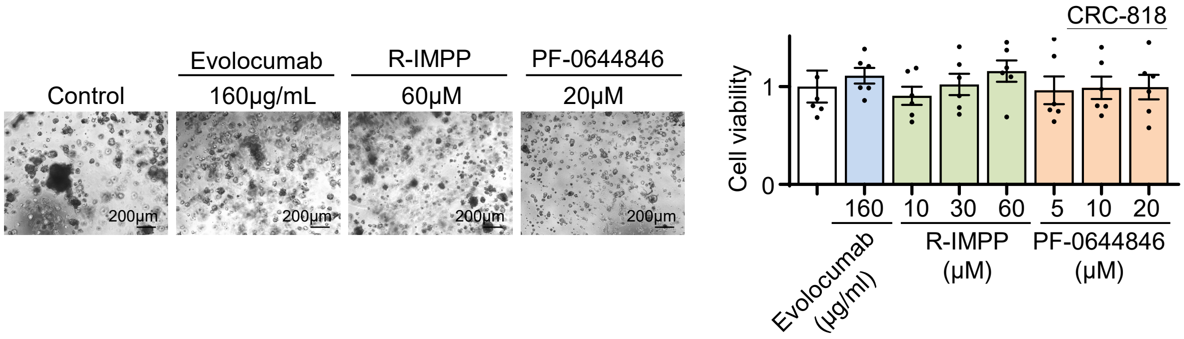

Supplementary Figure S15. Effect of PCSK9 inhibitors on CRC-818 organoids. Organoids were treated with these drugs for 5 days (n=6). Data shown are means of biological replicates; ± S.E.M. Two-tailed Student's t-test.

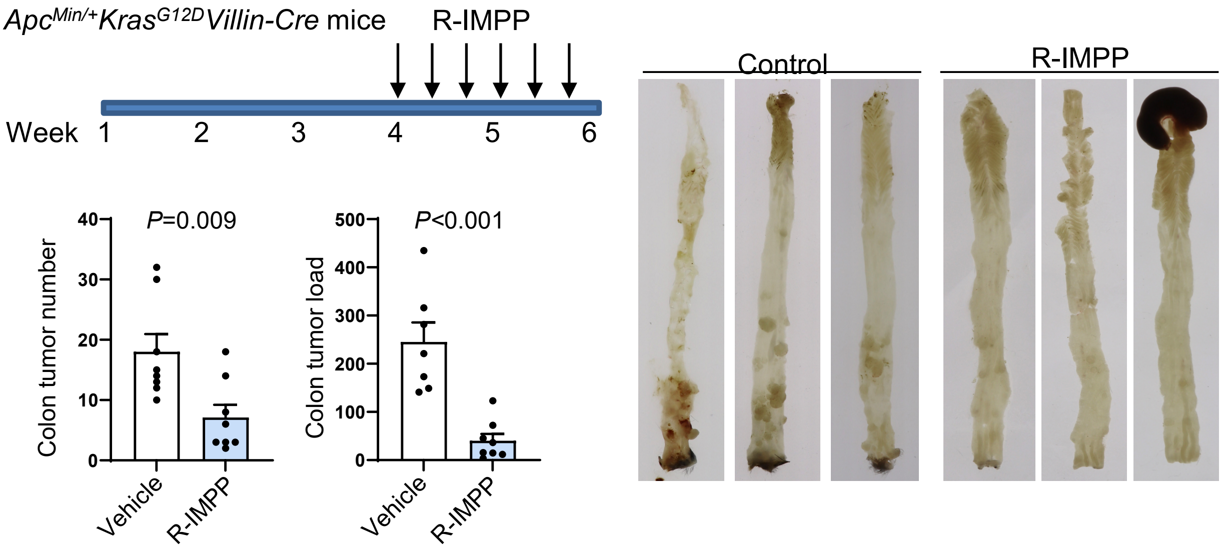

Supplementary Figure S16. Treatment of R-IMPP suppressed CRC in $Apc^{Min/+}Kras^{G12D/+}$Villin-Cre mice. Four weeks old mice were treated with vehicle or R-IMPP (50mg/kg, i.p., 3 times/week), and sacrificed at week 7 (n=1 experiment, n=7 for vehicle, n=8 for R-IMPP). Data shown are means of biological replicates; ± S.E.M. t-test (two-sided).

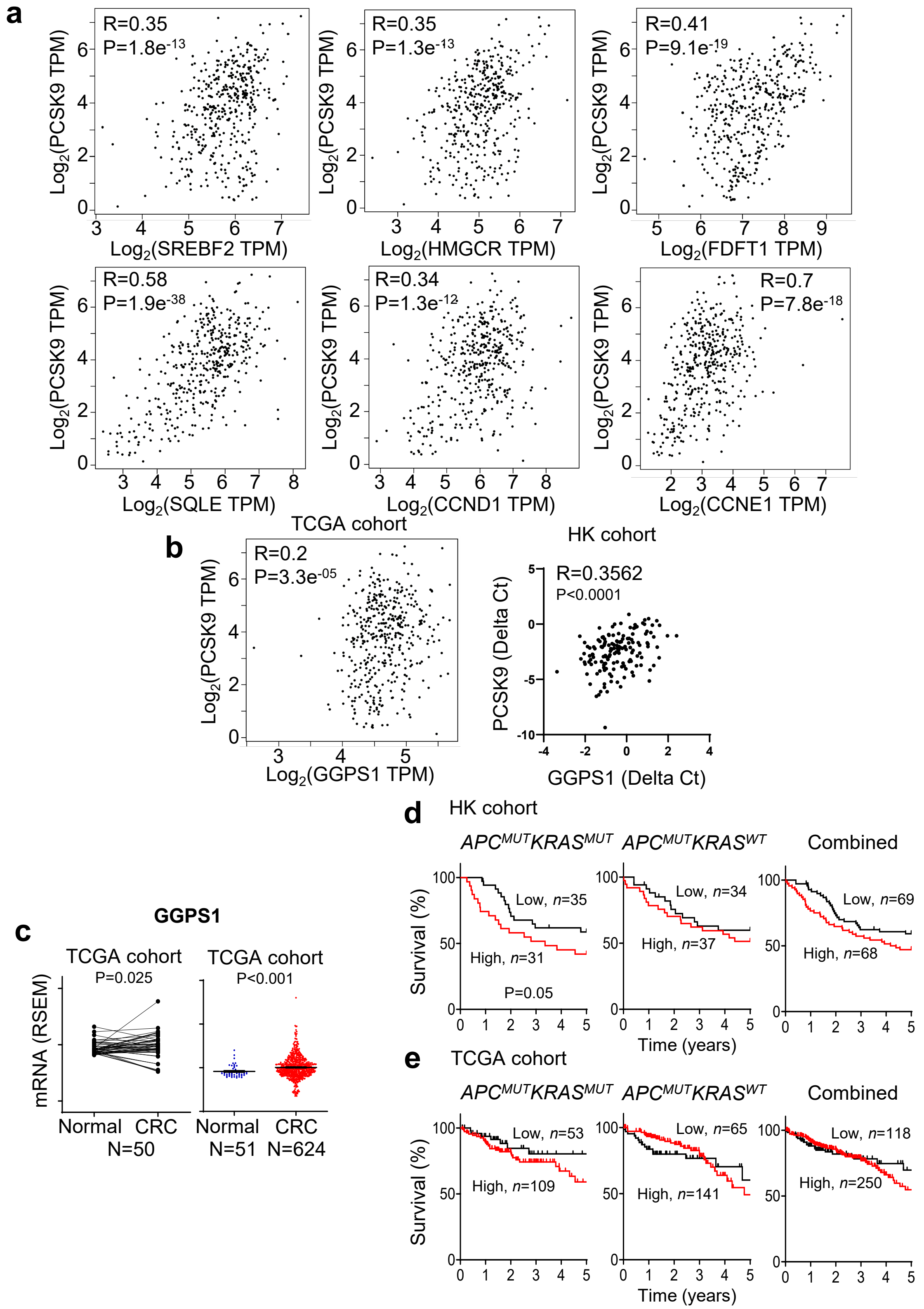

Supplementary Figure S17. a Correlation between PCSK9 mRNA and its downstream signaling in TCGA cohort. Spearman's rank-order correlation. **b** Correlation between PCSK9 and GGPS1 mRNA in TCGA and Hong Kong CRC cohort. Spearman's rank-order correlation. **c** GGPS1 mRNA is up-regulated in TCGA CRC dataset. t-test (two-tailed). **d** GGPS1 protein predicted poor survival of APC/KRAS-mutant CRC patients. Log rank test (two-tailed). **e** Association of GGPS1 mRNA expression with patient survival in TCGA cohort. Log rank test (two-tailed).

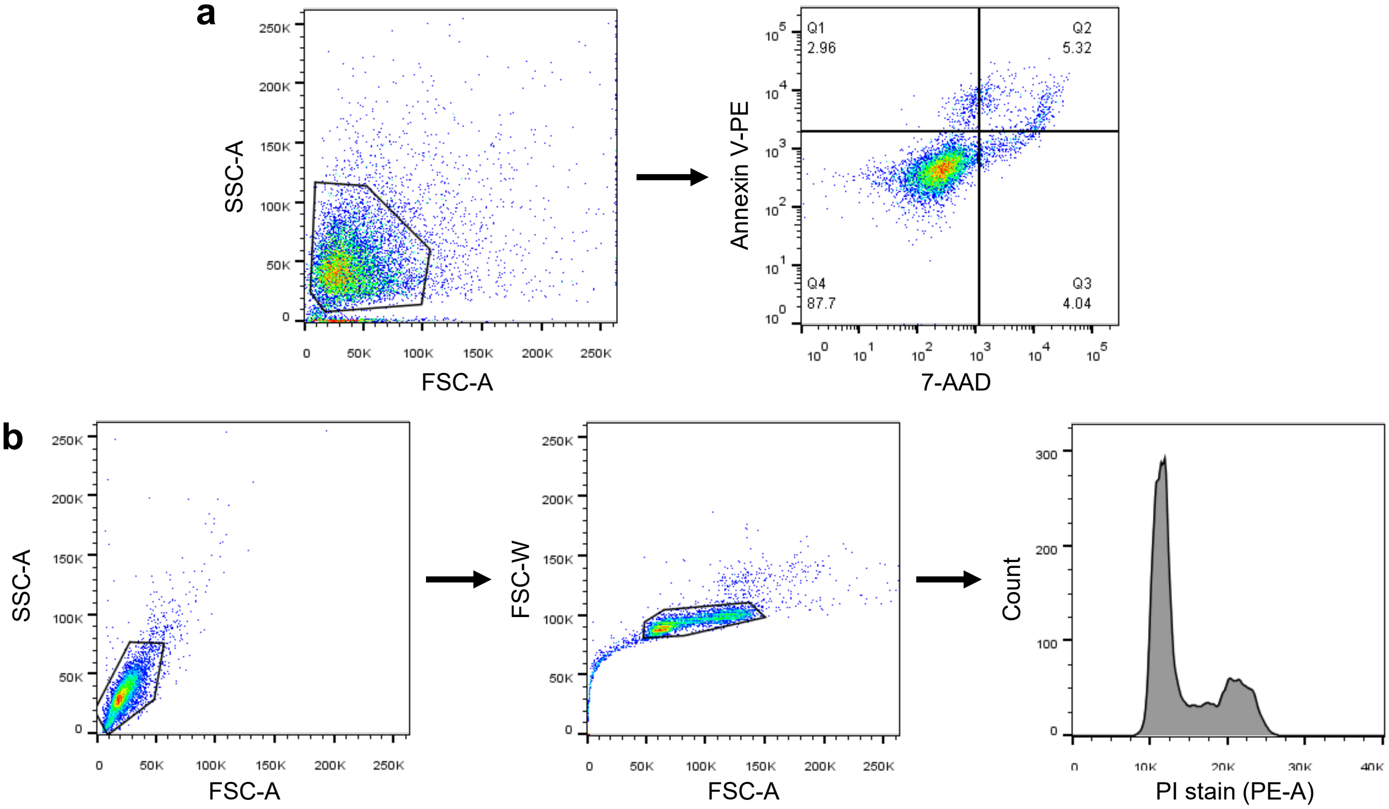

Supplementary Figure S18. Flow cytometry gating for **a** apoptosis and **b** cell cycle assays.

Figure 5E

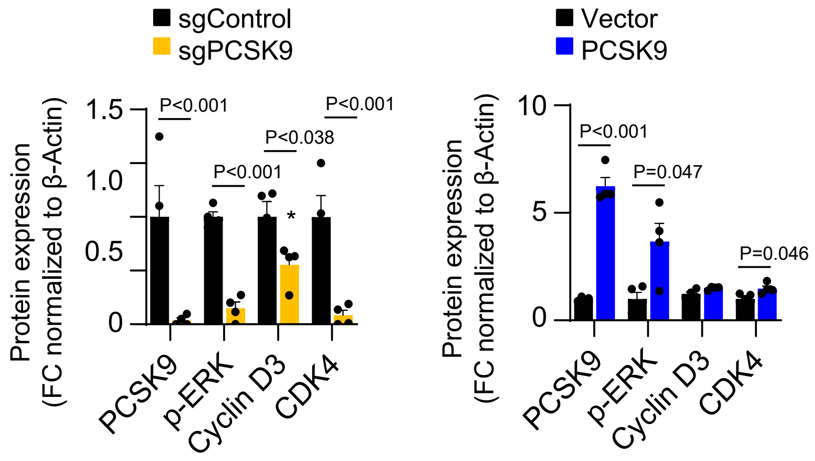

Figure 6E

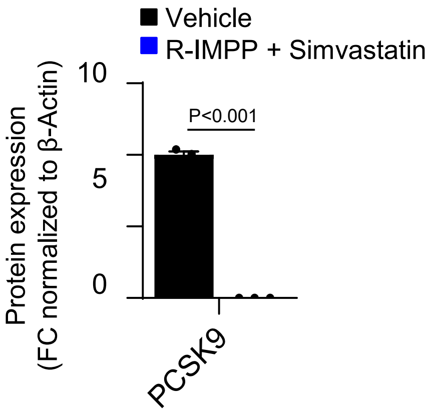

Figure 6F

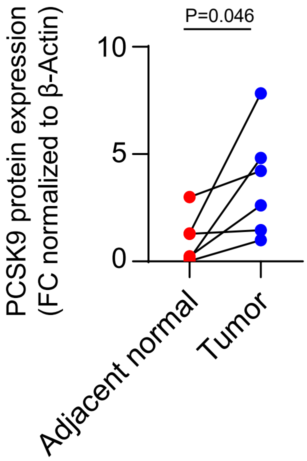

Supplementary Figure S19. Statistical analysis for Figure 5E, 6E and 6G. Data are presented as means ± S.E.M. t-test (two-sided).

Table S1. In silico prediction indicates that binding sites for PCSK9 promoter.

Transcript	PROMO	JASPAR
PCSK9-001	LEF1 (1)	LEF1 (1)
	TCF4 (1)	TCF3 (4)
		TCF4 (3)

Table S2. Clinicopathologic features of PCSK9 protein expression in CRC (TMA). Chi-square test (two-tailed).

**Variables**	**Low PCSK9 expression (N=94)**	**High PCSK9 expression (N=43)**	**P value**
**Age**, *y*, mean ± SD	67.2 ± 11.3	68.9 ± 12.5	0.492
**Gender**			0.061
Male	61 (75.3)	20 (24.7)	
Female	33 (58.9)	23 (41.1)	
**Location**			0.042
Colon	40 (59.7)	27 (40.3)	
Rectum	54 (77.1)	16 (22.9)	
**KRAS status**			0.463
Wildtype	51 (71.8)	20 (28.2)	
Mutant	43 (65.2)	23 (34.8)	
**TNM stage**			0.177
I	8 (72.7)	3 (27.2)	
II	34 (75.6)	11 (24.4)	
III	30 (73.2)	11 (26.8)	
IV	22 (55.0)	18 (45.0)	

Table S3. Clinicopathologic features of PCSK9 protein expression in CRC (TCGA). Chi-square test (two-tailed).

**Variables**	**Low PCSK9 expression (N=174)**	**High PCSK9 expression (N=194)**	**P value**
**Age**, *y*, mean ± SD	66.8 ± 11.1	65.35 ± 12.8	0.416
**Gender**			0.472
Male	98 (49.0)	102 (51.0)	
Female	76 (45.2)	92 (54.8)	
**Location**			0.958
Colon	126 (47.4)	140 (52.6)	
Rectum	48 (47.1)	54 (52.9)	
**KRAS status**			0.585
Wildtype	100 (48.5)	106 (51.5)	
Mutant	74 (45.7)	88 (54.3)	
**TNM stage**			0.661
I	34 (48.6)	36 (51.4)	
II	63 (51.2)	60 (48.8)	
III	51 (43.6)	66 (56.4)	
IV	26 (44.8)	32 (55.2)	

Table S4. Cox-regression analysis of potential survival predictor for patients with CRC (TMA). Cox-regression analysis (two-tailed).

**Variables**	**Univariate Cox regression analysis**		**Multivariate Cox regression analysis**	
	HR (95% C.I.)	P-Value	HR (95% C.I.)	P-Value
**Age**		0.066		0.034
≤65	1		1	
>65	1.93 (0.957-3.89)		2.23 (1.08-4.57)	
**Gender**		0.970		0.059
Male	1		1	
Female	0.987 (0.49-1.98)		0.436 (0.18-1.03)	
**TNM stage**		0.000		0.000
Early (I & II)	1		1	
Late (III & IV)	8.15 (3.32-20.03)		9.90 (3.80-25.79)	
**PCSK9 protein**		0.003		0.007
Low	1		1	
High	2.99 (1.49-6.02)		3.21 (1.37-7.51)	

Table S5. Cox-regression analysis of potential survival predictor for patients with CRC (TCGA). Cox-regression analysis (two-tailed).

**Variables**	**Univariate Cox regression analysis**		**Multivariate Cox regression analysis**	
	HR (95% C.I.)	P-Value	HR (95% C.I.)	P-Value
**Age**		0.063		0.200
≤65	1		1	
>65	2.21 (0.96-5.08)		1.80 (0.73-4.44)	
**Gender**		0.471		0.718
Male	1		1	
Female	0.78 (0.39-1.54)		0.88 (0.43-1.79)	
**TNM stage**		0.000		0.000
Early (I & II)	1		1	
Late (III & IV)	4.18 (1.98-8.79)		4.38 (2.08-9.23)	
**PCSK9 protein**		0.047		0.022
Low	1		1	
High	2.10 (1.01-4.36)		2.29 (1.10-4.77)	

Table S6. List of antibodies used in this study

Antibody	Company	Catalogue number
β-Catenin	Santa Cruz Biotechnology	sc-7199 (1:500)
Active β-Catenin	Cell Signalling Technology	8814 (1:1000)
β-Catenin (ChIP grade)	Abcam	ab227499 (1:1000)
CDK4	Cell Signalling Technology	12790 (1:1000)
Cleaved PARP	Cell Signalling Technology	5625 (1:1000)
Cyclin D1	Cell Signalling Technology	2922S (1:1000)
Cyclin D3	Cell Signalling Technology	2936S (1:1000)
FDFT1	Abcam	ab195046 (1:1000)
GGPS1	Abcam	ab167168 (1:1000)
HMGCR	Abcam	ab174830 (1:1000)
KRAS	Abcam	ab180772 (1:1000)
Lamin AC	Cell Signalling Technology	4777 (1:1000)
LDLR	Abcam	ab52818 (1:500)
MYC	Abcam	ab32072 (1:1000)
Na^+/K^+-ATPase	Abcam	ab76020 (1:1000)
p-ERK1/2	Cell Signalling Technology	4377 (1:1000)
p-MEK1/2	Cell Signalling Technology	9154 (1:1000)
p-p90RSK	Cell Signalling Technology	11989 (1:1000)
p27kip1	Cell Signalling Technology	3686 (1:1000)
PCSK9	Abcam	ab181142 (1:1000)
SQLE	Abcam	ab76896 (1:1000)
SREBP2	R&D Systems	AF7119 (1:200)
Total ERK1/2	Abcam	ab184699 (1:1000)
Total MEK1/2	Abcam	ab178876 (1:1000)
β-Actin (13E5)	Cell Signalling Technology	4970 (1:1000)
GAPDH (FL-335)	Santa Cruz Biotechnology	sc-25778 (1:500)

Table S7: Primers used in this study

Primer	Sequence	Application
ABCA1 mRNA	F: 5′-GAAGTACATCAGAACATGGGC-3′<br>R: 5′-GATCAAAGCCATGGCTGTAG-3′	qPCR
ABCG5 mRNA	F: 5′-TCCTGAGGAGAGTGACAAGAAAC-3′<br>R: 5′-ACGGGAAACAGATTCACAGC-3′	qPCR
ABCG8 mRNA	F: 5′-GGAACCCAGGAATCCTTATTCTC-3′<br>R: 5′-GGTCAGGTCCACATAGAAGTCAG-3′	qPCR
DHCR7 mRNA	F: 5′-CGCAGGACTTTAGCCGGT-3′<br>R: 5′-TGTCATTGGTGACGCCATCT-3′	qPCR
DHCR24 mRNA	F: 5′-TGAAGACAAACCGAGAGGGC-3′<br>R: 5′-CAGCCAAAGAGGTAGCGGAA-3′	qPCR
FDFT1 mRNA	F: 5′-CCACCCCGAAGAGTTCTACAA-3′<br>R: 5′-TGCGACTGGTCTGATTGAGATA-3′	qPCR
FDPS mRNA	F: 5′-CAGCTTTCTACTCCTTCTACCTTCC-3′<br>R: 5′-GCTCCTTCTCGCCATCAAT-3′	qPCR
GGPS1 mRNA	F: 5′-CCAGGTAAACAAGTGAGAACCAA-3′<br>R: 5′-CGTCGGAGTTTTGAGTTGTCT-3′	qPCR
HMGCR mRNA	F: 5′-TGATTGACCTTTCCAGAGCAAG-3′<br>R: 5′-CTAAAATTGCCATTCCACGAGC-3′	qPCR
HMGCS1 mRNA	F: 5′-CATTAGACCGCTGCTATTCTGTC-3′<br>R: 5′-TTCAGCAACATCCGAGCTAGA-3′	qPCR
IDI1 mRNA	F: 5′-TGGATAAAACCCCTGTGGTG-3′<br>R: 5′-CAACATCCGGCATAACTGTG-3′	qPCR
LDLR mRNA	F: 5′-TACAAGTGGGTCTGCGATGG-3′<br>R: 5′-TGAAGTCCCCGGATTTGCAG-3′	qPCR
MVD mRNA	F: 5′-GTAAGTGGCTGTGGAGCTGG-3′<br>R: 5′-GGAGTTGATGGGCAGAACCA-3′	qPCR
MVK mRNA	F: 5′-CTCTGATTGGCTGGCCTGAA-3′<br>R: 5′-CCAACTCCCACAACCCAGAG-3′	qPCR
NPC1L1 mRNA	F: 5′-TATCTTCCCTGGTTCCTGAACGAC-3′<br>R: 5′-CCGCAGAGCTTCTGTGTAATCC-3′	qPCR
PCSK9 mRNA	F: 5′-GACGATGCCTGCCTCTACTC-3′<br>R: 5′-CCAATGATGTCCTCCCCTGG-3′	qPCR
PMVK mRNA	F: 5′-GCTGATGTCTGTGCTGTCCT-3′<br>R: 5′-GAAAGGCCTCCTTGTAGGTG-3′	qPCR

SQLE mRNA	F: 5′-TGACAATTCTCATCTGAGGTCCA-3′<br>R: 5′-CAGGGATACCCTTTAGCAGTTTT-3′	qPCR
ACTIN mRNA	F: 5′-AGAGCTACGAGCTGCCTGAC-3′<br>R: 5′-AGCACTGTGTTGGCGTACAG-3′	qPCR
Acat2 mRNA	F: 5′-CCCGTGGTCATCGTCTCAG-3′<br>R: 5′-GGACAGGGCACCATTGAAGG-3′	qPCR
Dhcr7 mRNA	F: 5′-AGGCTGGATCTCAAGGACAAT-3′<br>R: 5′-GCCAGACTAGCATGGCCTG-3′	qPCR
Dhcr24 mRNA	F: 5′-CGCTGCGAGTCGGAAAGTA-3′<br>R: 5′-GTCACCTGACCCATAGACACC-3′	qPCR
Fdft1 mRNA	F: 5′-ATGGAGTTCGTCAAGTGTCTAGG-3′<br>R: 5′-CGTGCCGTATGTCCCCATC-3′	qPCR
Fdps mRNA	F: 5′-ATGCCATCAACGACGCTCTG-3′<br>R: 5′-CCGATCTCTGTCTGATAGGAACT-3′	qPCR
Hmgcr mRNA	F: 5′-AGCTTGCCCGAATTGTATGTG-3′<br>R: 5′-TCTGTTGTGAACCATGTGACTTC-3′	qPCR
Hmgcs1 mRNA	F: 5′-AACTGGTGCAGAAATCTCTAGC-3′<br>R: 5′-GGTTGAATAGCTCAGAACTAGCC-3′	qPCR
Idi1 mRNA	F: 5′-ACCAGCCATCTTGATGAAAAACA-3′<br>R: 5′-CAGCAACTATTGGTGAAACAACC-3′	qPCR
Mvd mRNA	F: 5′-ATGGCCTCAGAAAAGCCTCAG-3′<br>R: 5′-TGGTCGTTTTTAGCTGGTCCT-3′	qPCR
Mvk mRNA	F: 5′-AGCGTCAATTTACCCAACATCG-3′<br>R: 5′-GAGACATCACCTTGCTCAAGAAA-3′	qPCR
Pcsk9 mRNA	F: 5′-GAGACCCAGAGGCTACAGATT-3′<br>R: 5′-AATGTACTCCACATGGGGCAA-3′	qPCR
PCSK9 ChIP	F: 5′-CACTGTCTTTGTGCACTGGC-3′<br>R: 5′-TGCTCTTTCTGGAAGGGCTG-3′	ChIP-PCR
