# Supplementary material for: The cholesterol uptake regulator PCSK9 promotes and is a therapeutic target in APC/KRAS-mutant colorectal cancer
Source: Nat Commun. 2022 Jul 8;13:3971. doi: 10.1038/s41467-022-31663-z (PMC9270407; doi:10.1038/s41467-022-31663-z)
Supplement: Supplementary file 3 — Reporting Summary [file 41467_2022_31663_MOESM3_ESM.pdf]

# nature portfolio

Corresponding author(s): Chi Chun Wong, Jun Yu

Last updated by author(s): Feb 10, 2022

# Reporting Summary

Nature Portfolio wishes to improve the reproducibility of the work that we publish. This form provides structure for consistency and transparency in reporting. For further information on Nature Portfolio policies, see our Editorial Policies and the Editorial Policy Checklist.

## Statistics

For all statistical analyses, confirm that the following items are present in the figure legend, table legend, main text, or Methods section.

| n/a | Confirmed | |
|---|---|---|
| ☐ | ☒ | The exact sample size (*n*) for each experimental group/condition, given as a discrete number and unit of measurement |
| ☐ | ☒ | A statement on whether measurements were taken from distinct samples or whether the same sample was measured repeatedly |
| ☐ | ☒ | The statistical test(s) used AND whether they are one- or two-sided<br>*Only common tests should be described solely by name; describe more complex techniques in the Methods section.* |
| ☐ | ☒ | A description of all covariates tested |
| ☐ | ☒ | A description of any assumptions or corrections, such as tests of normality and adjustment for multiple comparisons |
| ☐ | ☒ | A full description of the statistical parameters including central tendency (e.g. means) or other basic estimates (e.g. regression coefficient) AND variation (e.g. standard deviation) or associated estimates of uncertainty (e.g. confidence intervals) |
| ☐ | ☒ | For null hypothesis testing, the test statistic (e.g. *F*, *t*, *r*) with confidence intervals, effect sizes, degrees of freedom and *P* value noted<br>*Give P values as exact values whenever suitable.* |
| ☒ | ☐ | For Bayesian analysis, information on the choice of priors and Markov chain Monte Carlo settings |
| ☒ | ☐ | For hierarchical and complex designs, identification of the appropriate level for tests and full reporting of outcomes |
| ☒ | ☐ | Estimates of effect sizes (e.g. Cohen's *d*, Pearson's *r*), indicating how they were calculated |

*Our web collection on statistics for biologists contains articles on many of the points above.*

## Software and code

Policy information about availability of computer code

| | |
|---|---|
| Data collection | Image Lab software v6.1.0 (Bio-rad), Quantstudio Real Time PCR software v1.7.2 (Applied Biosystems), Vsviewer (Metasystems), Leica Las X v5.02 (Leica), Xcalibur 4.2 (ThermoFisher) |
| Data analysis | GraphPad Prism v9, IBM SPSS statistics v27 |

For manuscripts utilizing custom algorithms or software that are central to the research but not yet described in published literature, software must be made available to editors and reviewers. We strongly encourage code deposition in a community repository (e.g. GitHub). See the Nature Portfolio guidelines for submitting code & software for further information.

## Data

Policy information about availability of data

All manuscripts must include a data availability statement. This statement should provide the following information, where applicable:

- Accession codes, unique identifiers, or web links for publicly available datasets
- A description of any restrictions on data availability
- For clinical datasets or third party data, please ensure that the statement adheres to our policy

Source data are provided with this paper. TCGA dataset was accessed using the UCSC Xena Browser (https://xenabrowser.net/)

# Field-specific reporting

Please select the one below that is the best fit for your research. If you are not sure, read the appropriate sections before making your selection.

☒ Life sciences ☐ Behavioural & social sciences ☐ Ecological, evolutionary & environmental sciences

For a reference copy of the document with all sections, see nature.com/documents/nr-reporting-summary-flat.pdf

# Life sciences study design

All studies must disclose on these points even when the disclosure is negative.

| | |
|---|---|
| Sample size | Sample size for in vitro and animal work were decided based on experience from similar experiences in our laboratory (Wong et al. Gastroenterology, 2020;159(6):2163-2180; Wong et al. Gastroenterology, 2016;151(5):945-960). |
| Data exclusions | No exclusion of data was performed. |
| Replication | All in vitro work was repeated in at least 2 independent experiments with good reproducibility. |
| Randomization | All samples and animals were analyzed and allocated randomly. |
| Blinding | The investigators were not blinded to allocation during experiments. Blinding was not possible as the same investigator performed the experiment and analysis. Pathological evaluation was performed in a blinded manner by a experienced pathologist. |

# Reporting for specific materials, systems and methods

We require information from authors about some types of materials, experimental systems and methods used in many studies. Here, indicate whether each material, system or method listed is relevant to your study. If you are not sure if a list item applies to your research, read the appropriate section before selecting a response.

## Materials & experimental systems

| n/a | Involved in the study | |
|---|---|---|
| ☐ | ☒ | Antibodies |
| ☐ | ☒ | Eukaryotic cell lines |
| ☒ | ☐ | Palaeontology and archaeology |
| ☐ | ☒ | Animals and other organisms |
| ☐ | ☒ | Human research participants |
| ☒ | ☐ | Clinical data |
| ☒ | ☐ | Dual use research of concern |

## Methods

| n/a | Involved in the study | |
|---|---|---|
| ☒ | ☐ | ChIP-seq |
| ☐ | ☒ | Flow cytometry |
| ☒ | ☐ | MRI-based neuroimaging |

## Antibodies

| | |
|---|---|
| Antibodies used | β-Catenin (Santa Cruz Biotechnology, sc-7199, H-102, 1:500), Active β-Catenin (Cell Signalling Technology, 8814, D13A1, 1:1000), β-Catenin (ChIP grade, Abcam, ab227499, 1:1000, 2µg per reaction); CDK4 (Cell Signalling Technology, 12790, D9G3E, 1:1000), Cleaved PARP (Cell Signalling Technology, 5625, D64E10, 1:1000), Cyclin D1 (Cell Signalling Technology, 2922S, 1:1000), Cyclin D3 (Cell Signalling Technology, 2936S, DCS22, 1:1000), FDFT1 (Abcam, ab195046, EPR16481, 1:1000), GGPS1 (Abcam, ab167168, EPR9682, 1:1000), HMGCR (Abcam, ab174830, EPR1685(N), 1:1000), KRAS (Abcam, ab180772, 1:1000), Lamin AC (Cell Signalling Technology, 4777, 4C11, 1:1000), LDLR (Abcam, ab52818, EP1553Y, 1:500), MYC (Abcam, ab32072, Y69, 1:1000), Na+/K+-ATPase (Abcam, ab76020, EP1845Y, 1:1000), p-ERK1/2 (Cell Signalling Technology, 4377, 197G2, 1:1000), p-MEK1/2 (Cell Signalling Technology, 9154, 41G9, 1:1000), p-p90RSK (Cell Signalling Technology, 11989, D3H11, 1:1000), p27kip1 (Cell Signalling Technology, 3686, D69C12, 1:1000), PCSK9 (Abcam, ab181142, EPR7627(2), 1:1000), SQLE (Abcam, ab76896, 1:1000), SREBP2 (R&D Systems, AF7119, 1:200), Total ERK1/2 (Abcam, ab184699, EPR17526, 1:1000), Total MEK1/2 (Abcam, ab178876, EPR16667, 1:1000), β-Actin (Cell Signalling Technology, 4970, 13E5, 1:1000), GAPDH (Santa Cruz Biotechnology, sc-25778, FL-335, 1:500) |
| Validation | All antibodies are obtained from commercial sources, and validation was available from the websites of respective vendors:<br>https://www.abcam.com/<br>https://www.cellsignal.com/<br>https://www.scbt.com/home<br>https://www.rndsystems.com/ |

# Eukaryotic cell lines

Policy information about cell lines

| | |
|---|---|
| Cell line source(s) | DLD1, HCT116, LOVO and SW1116 cell lines were obtained from the American Type Culture Collection (Rockville, MD). DKS8 cells were from Prof. Senji Shirasawa, Fukuoka University. 1CT normal colonic cells and 1CT cells expressing shAPC (1CT-A) was obtained from Prof. Jerry Shay (UT Southwestern Medical Center, TX). |
| Authentication | All commercial cell lines (DLD1, HCT116, LOVO and SW1116) were authenticated by STR Profiling. Non-commercial cell lines (DKS8, 1CT, 1CT-A, 1CT-K, 1CT-AK) were not authenticated. |
| Mycoplasma contamination | All commercial cell lines (DLD1, HCT116, LOVO and SW1116) were negative for Mycoplasma. Non-commercial cell lines (DKS8, 1CT, 1CT-A, 1CT-K, 1CT-AK) were not tested. |
| Commonly misidentified lines (See ICLAC register) | No commonly misidentified cell lines were used in the study. |

# Animals and other organisms

Policy information about studies involving animals; ARRIVE guidelines recommended for reporting animal research

| | |
|---|---|
| Laboratory animals | Six-weeks-old female nude mice were used in the implantation of human tumor xenografts. Nude mice were obtained from the Chinese University of Hong Kong (CUHK) mouse repository. Apcmin/+KrasG12DVillin-Cre were used between aged 3 to 7 weeks, and it was purchased commercially from Nanjing Biomedical Research Institute, Nanjing University. All mice were maintained under specific pathogen-free conditions at the animal facility of CUHK. These mice were maintained in 12hour light/dark cycle, and the housing temperature and humidity were at 23 degrees and 45%, respectively. |
| Wild animals | This study did not involve wild animals. |
| Field-collected samples | This study did not involve field-collected samples. |
| Ethics oversight | All animal work is approved by the Animal Experimentation Ethics Committee of the Chinese University of Hong Kong (18-118-GRF). |

Note that full information on the approval of the study protocol must also be provided in the manuscript.

# Human research participants

Policy information about studies involving human research participants

| | |
|---|---|
| Population characteristics | Informed consent was obtained to collect human CRC tissues, and the study was approved by by ethics committee of the Chinese University of Hong Kong and the Beijing University Cancer Hospital (2019.425). All patients were treatment naive CRC patients. The age, gender and the pathological stage of patients have been documented. |
| Recruitment | Patients were enrolled in by their oncologist at the Chinese University of Hong Kong and the Beijing University Cancer Hospital. After obtaining informed consent from patients/immediate family of patients, tumor specimens were collected after surgical removal of tumors. Since the majority of the population in Hong Kong and Beijing are predominantly Chinese, this might lead to selection bias towards Chinese patients. |
| Ethics oversight | This study was approved by ethics committee of the Chinese University of Hong Kong and the Beijing University Cancer Hospital. |

Note that full information on the approval of the study protocol must also be provided in the manuscript.

# Flow Cytometry

## Plots

Confirm that:

- ☒ The axis labels state the marker and fluorochrome used (e.g. CD4-FITC).
- ☒ The axis scales are clearly visible. Include numbers along axes only for bottom left plot of group (a 'group' is an analysis of identical markers).
- ☒ All plots are contour plots with outliers or pseudocolor plots.
- ☒ A numerical value for number of cells or percentage (with statistics) is provided.

## Methodology

| | |
|---|---|
| Sample preparation | Cells were harvested by trypsinization into single cells prior to staining. |
| Instrument | BD FACS Celesta |

| | |
|---|---|
| Software | FlowJo v10 |
| Cell population abundance | Abundance and purity of cell population determined by FlowJo V10 software with identical gating strategy across each set of samples |
| Gating strategy | FSC-A/SSC-A was used for gating colon cells. FSC-W/FSCH was used for gating singlets. The specific gating strategies are presented in Supplementary Figure S18. |

☒ Tick this box to confirm that a figure exemplifying the gating strategy is provided in the Supplementary Information.
