# Supplementary material for: The cholesterol uptake regulator PCSK9 promotes and is a therapeutic target in APC/KRAS-mutant colorectal cancer
Source: Nat Commun. 2022 Jul 8;13:3971. doi: 10.1038/s41467-022-31663-z (PMC9270407; doi:10.1038/s41467-022-31663-z)

Figure 1E

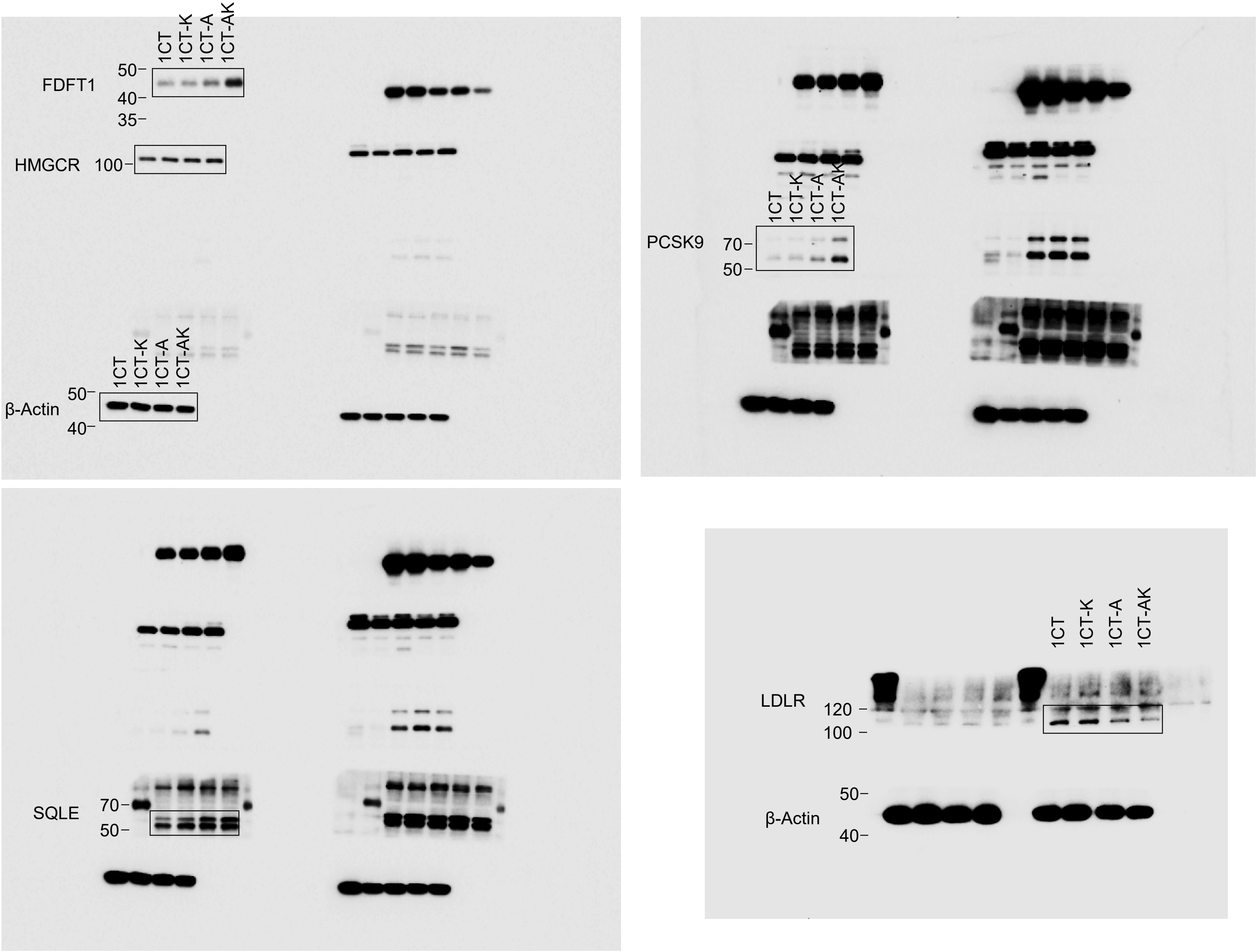
1CT
1CT-K
1CT-A
1CT-AK
FDFT1
50–
40–
35–
HMGCR
100–
β-Actin
50–
40–
PCSK9
70–
50–
SQLE
70–
50–
LDLR
120 –
100 –
β-Actin
50–
40–

Figure 2A

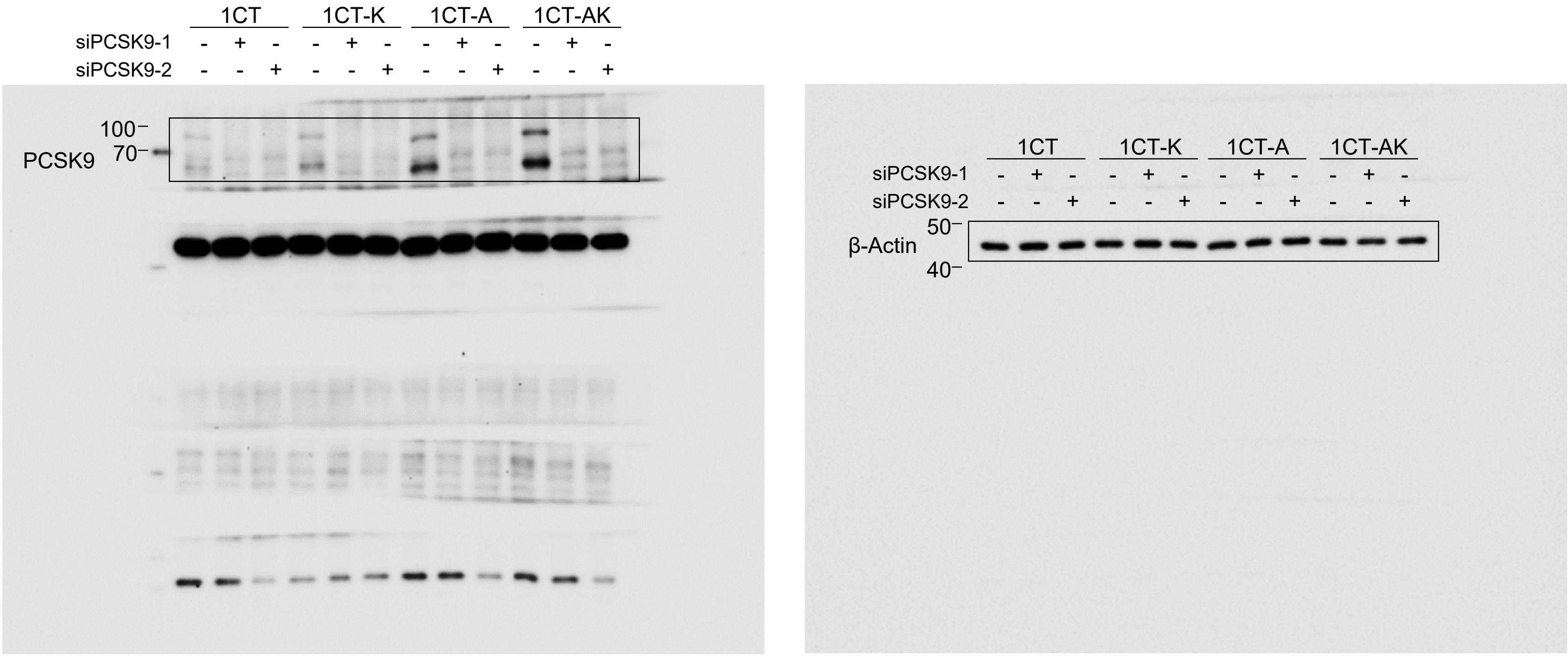
1CT
1CT-K
1CT-A
1CT-AK
siPCSK9-1
siPCSK9-2
100–
70–
PCSK9
1CT
1CT-K
1CT-A
1CT-AK
siPCSK9-1
siPCSK9-2
50–
β-Actin
40–

Figure 2D

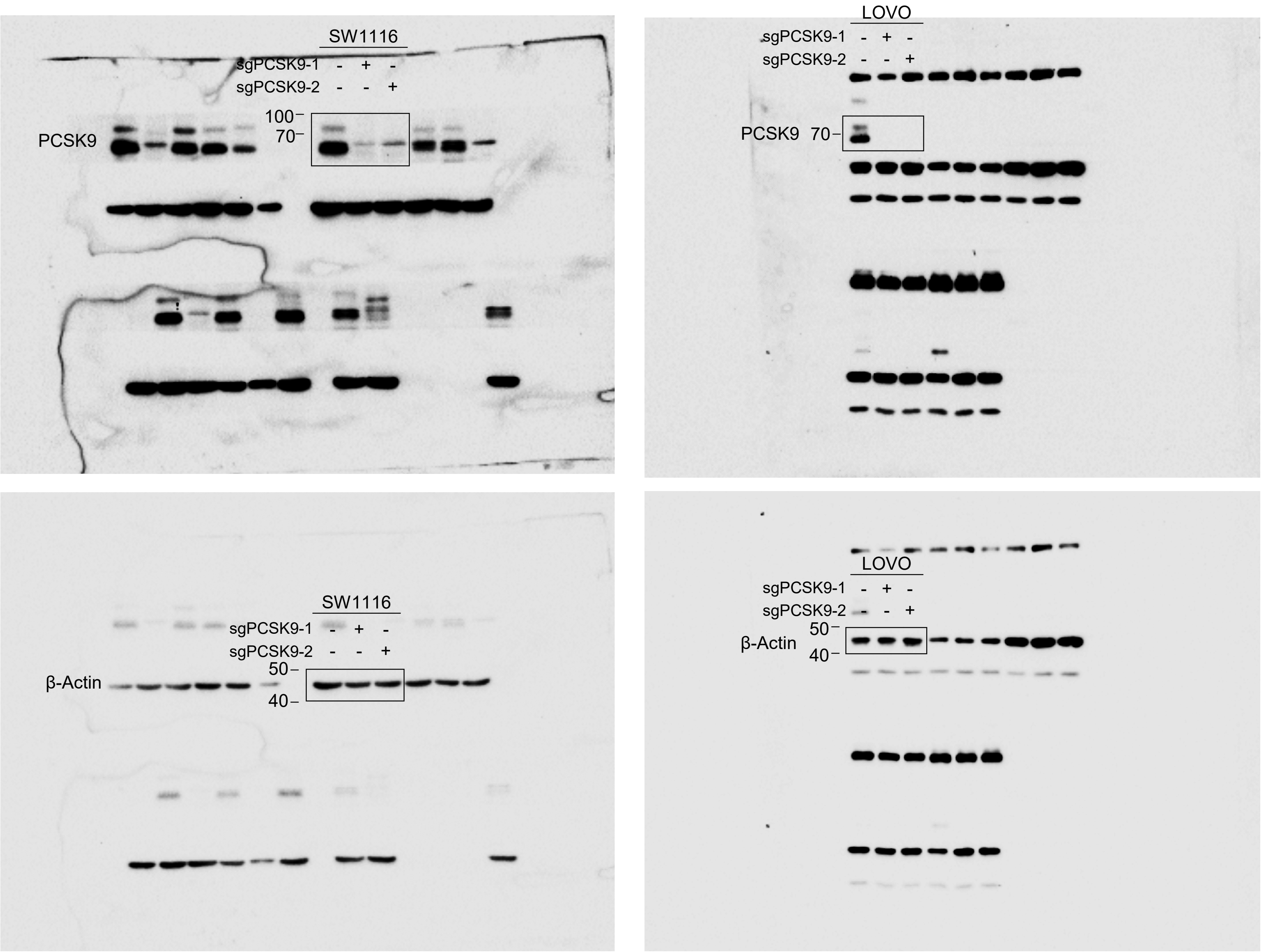
SW1116
sgPCSK9-1 - + -
sgPCSK9-2 - - +
PCSK9
100
70
LOVO
sgPCSK9-1 - + -
sgPCSK9-2 - - +
PCSK9 70
SW1116
sgPCSK9-1 - + -
sgPCSK9-2 - - +
β-Actin
50
40
LOVO
sgPCSK9-1 - + -
sgPCSK9-2 - - +
β-Actin
50
40

Figure 2H – left panel

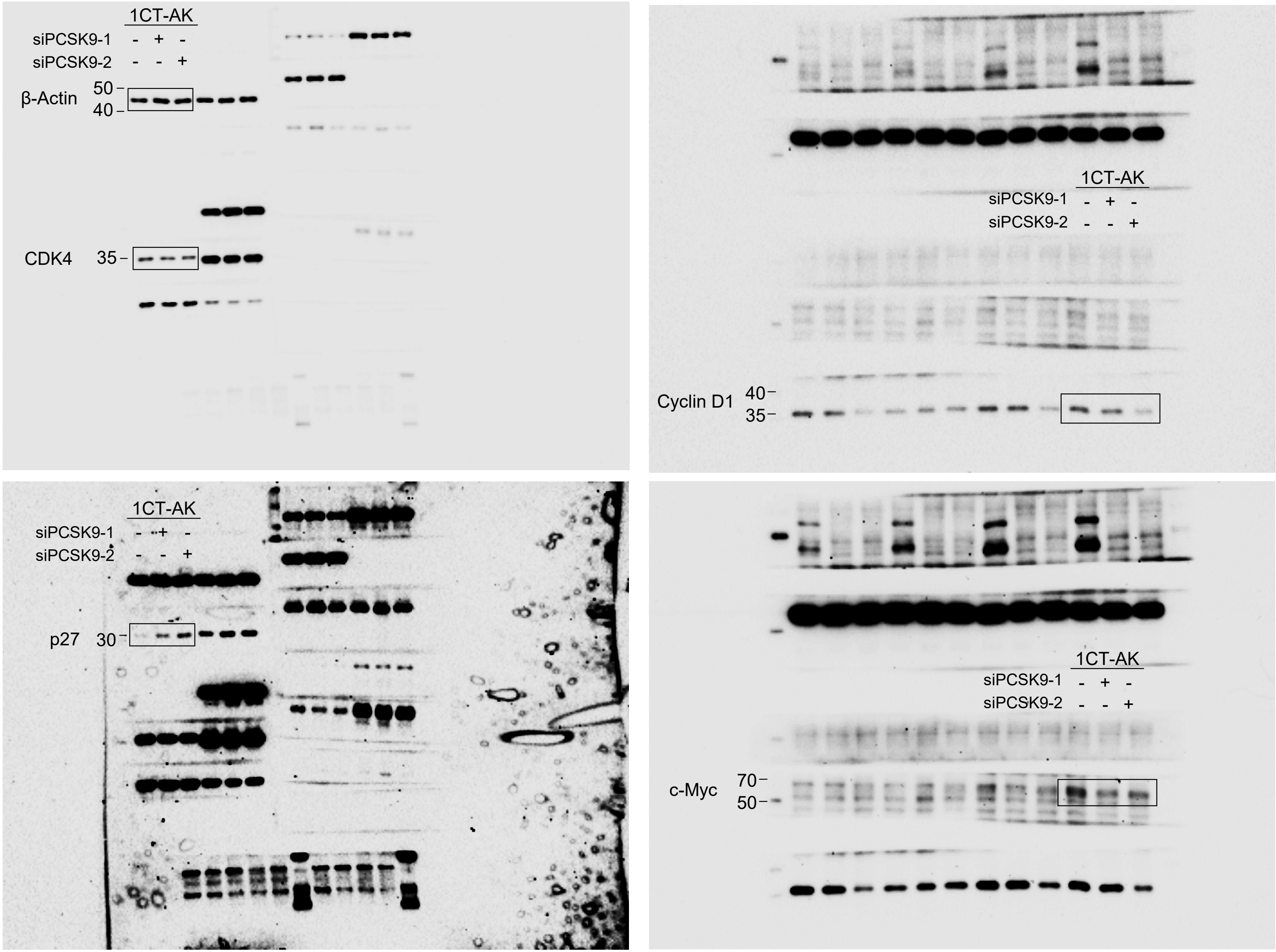

Figure 2H – right panel

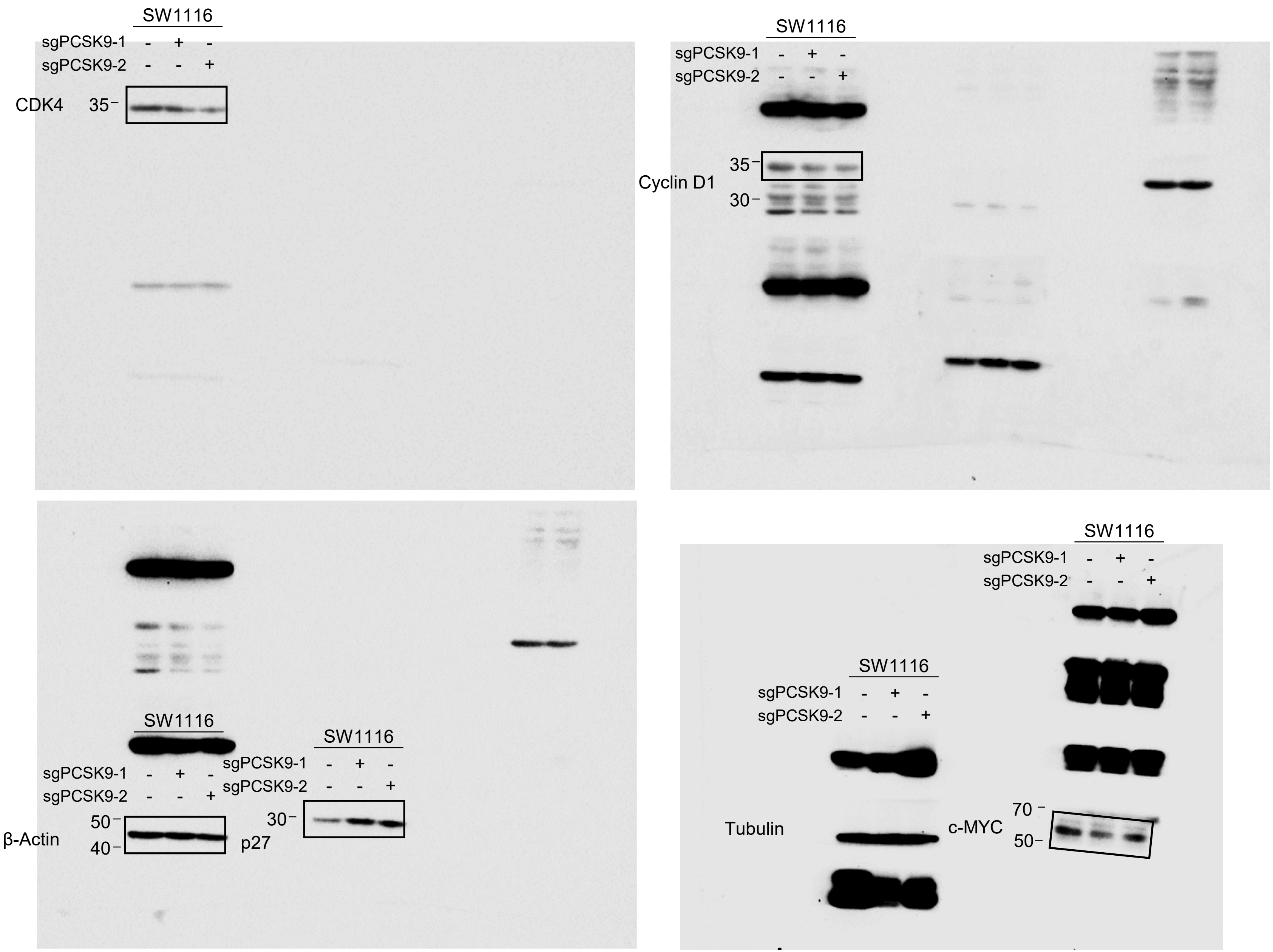

Figure 2K

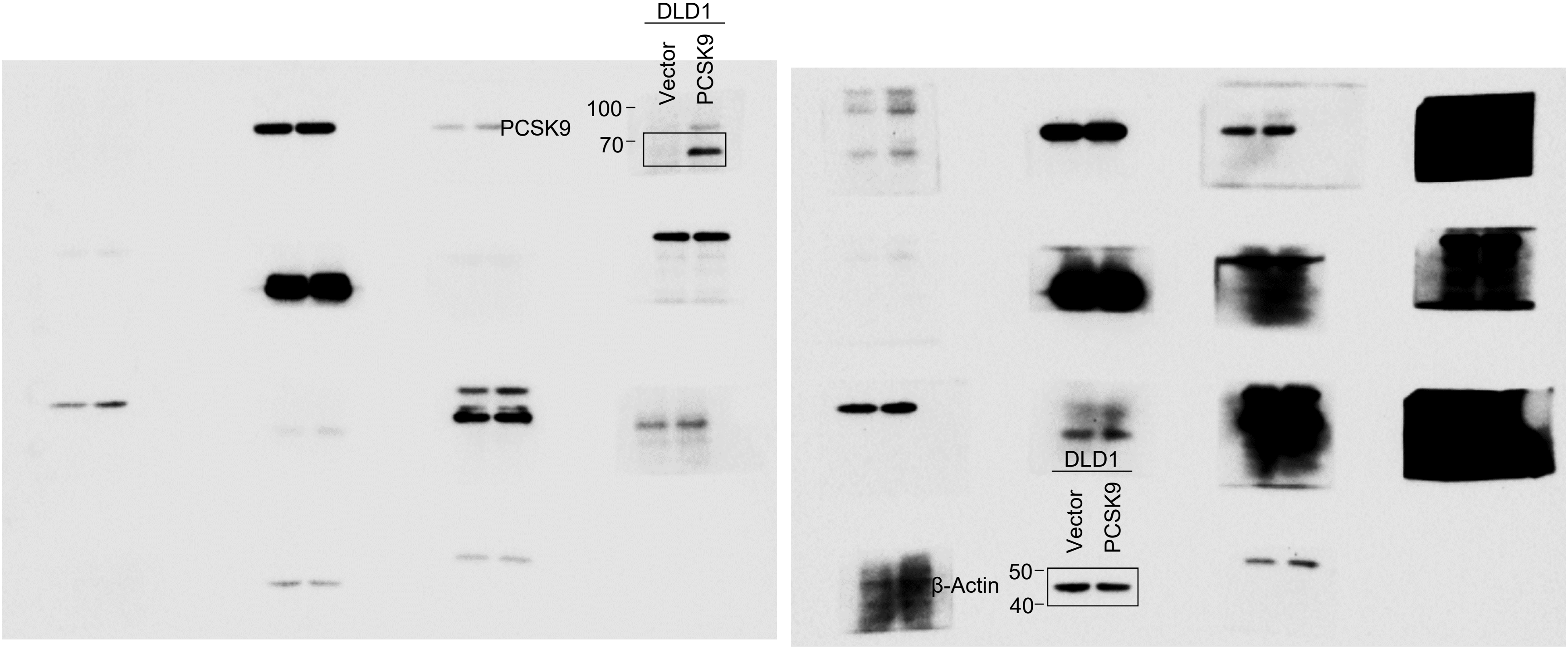

DLD1
Vector
PCSK9
100
70
PCSK9
DLD1
Vector
PCSK9
50
40
β-Actin

Figure 2N

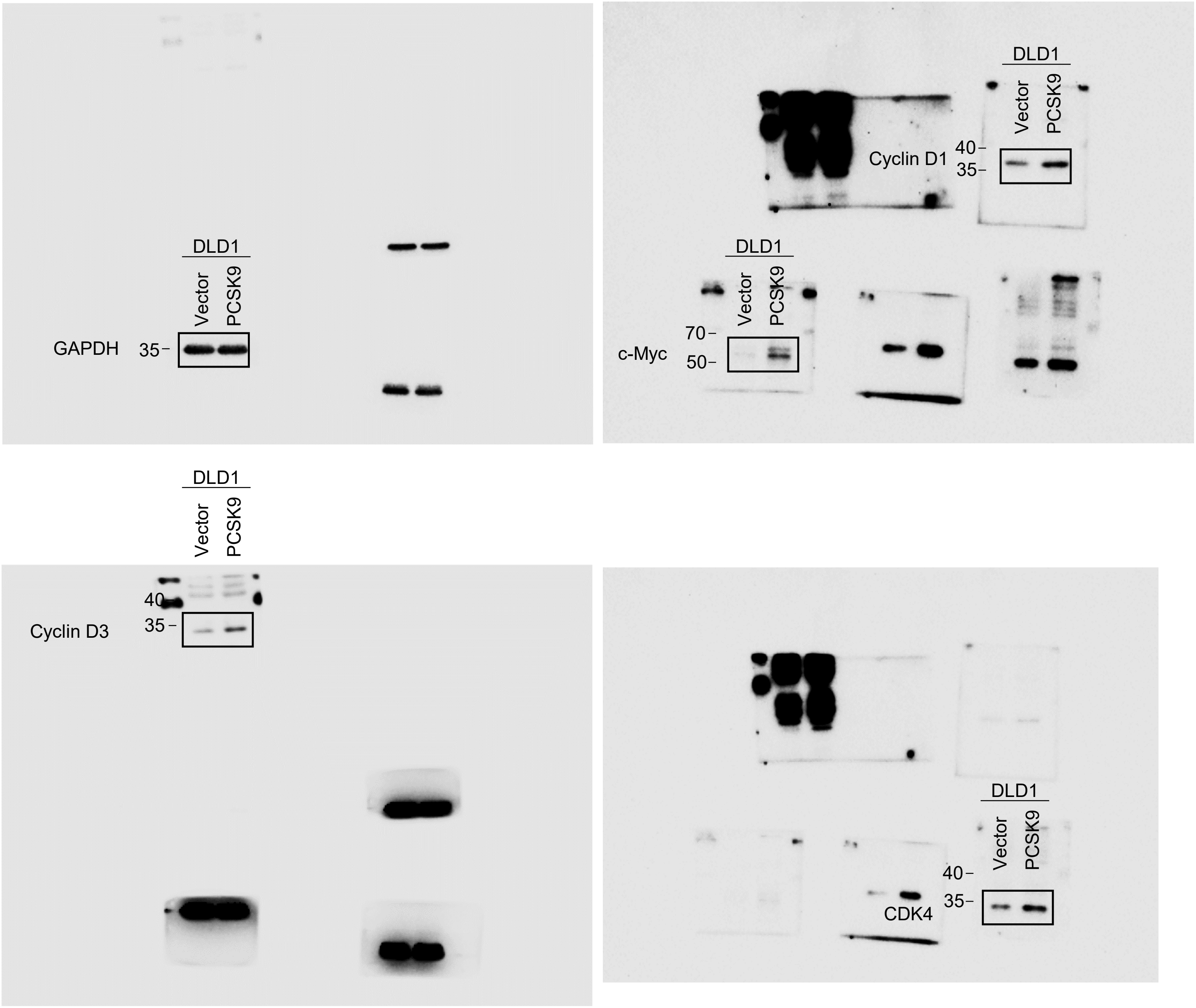
DLD1
Vector
PCSK9
GAPDH
35
Cyclin D1
40
35
c-Myc
70
50
Cyclin D3
CDK4

Figure 3C – 1CT-AK

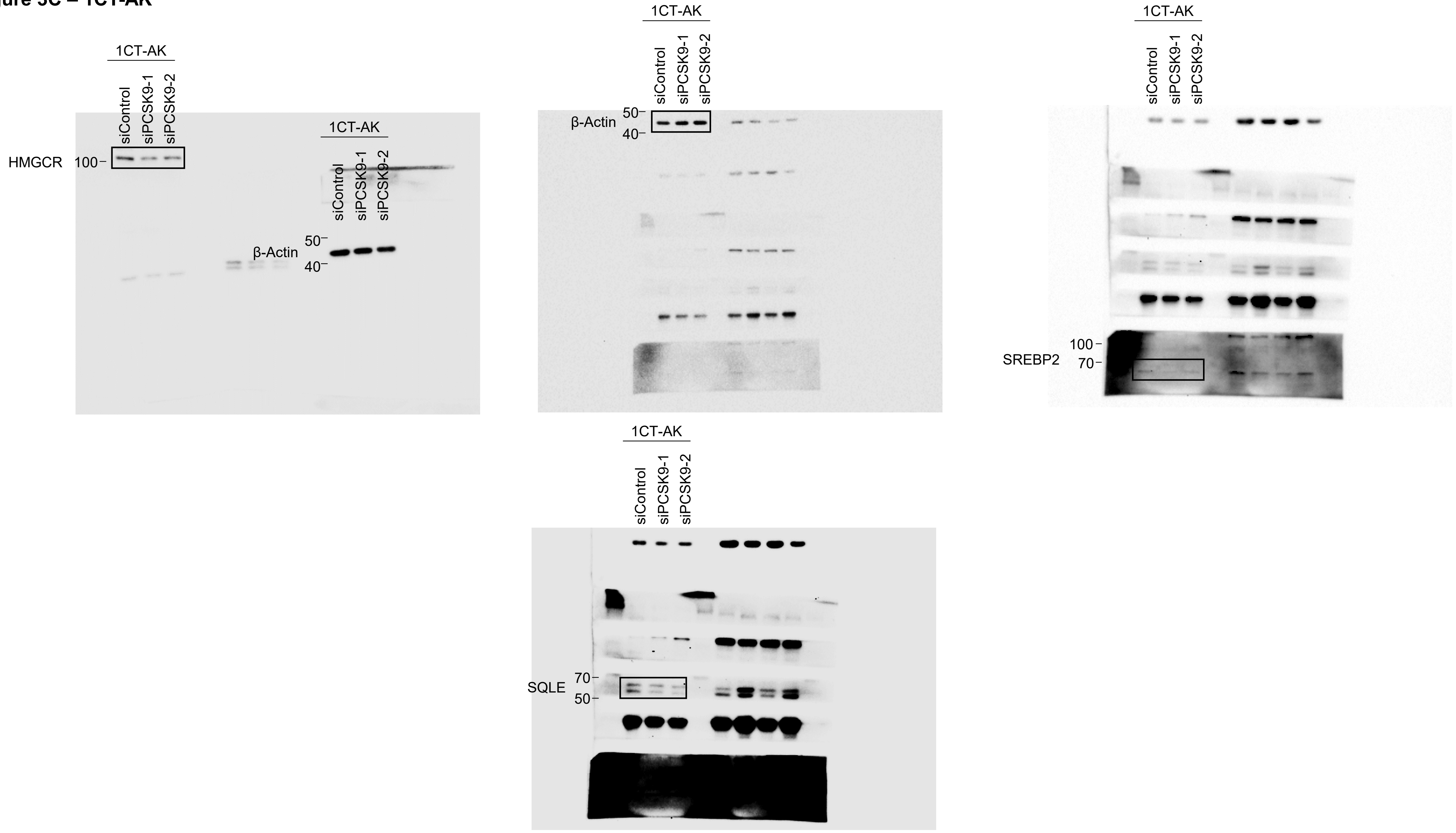

Figure 3C – LOVO

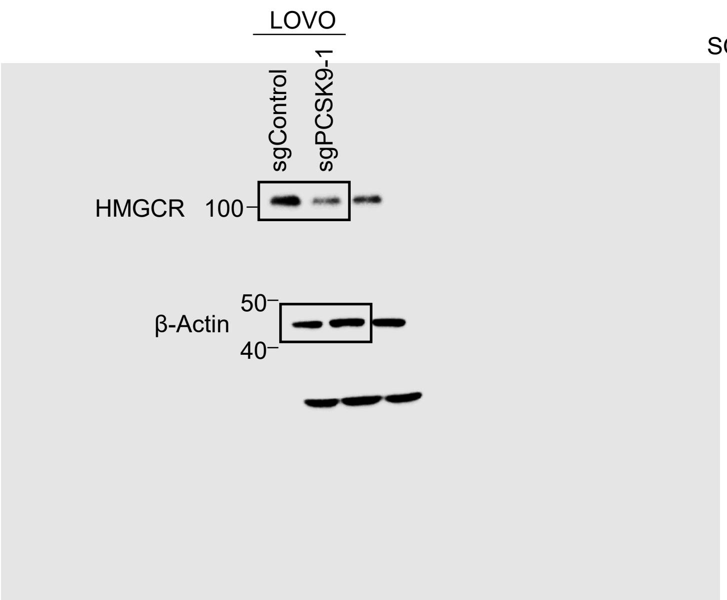

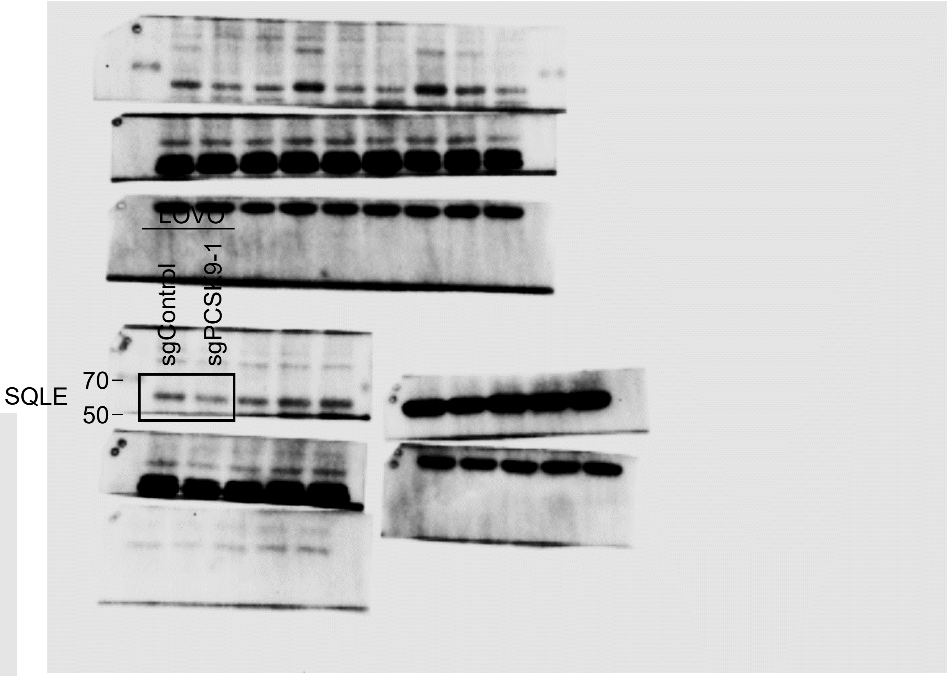

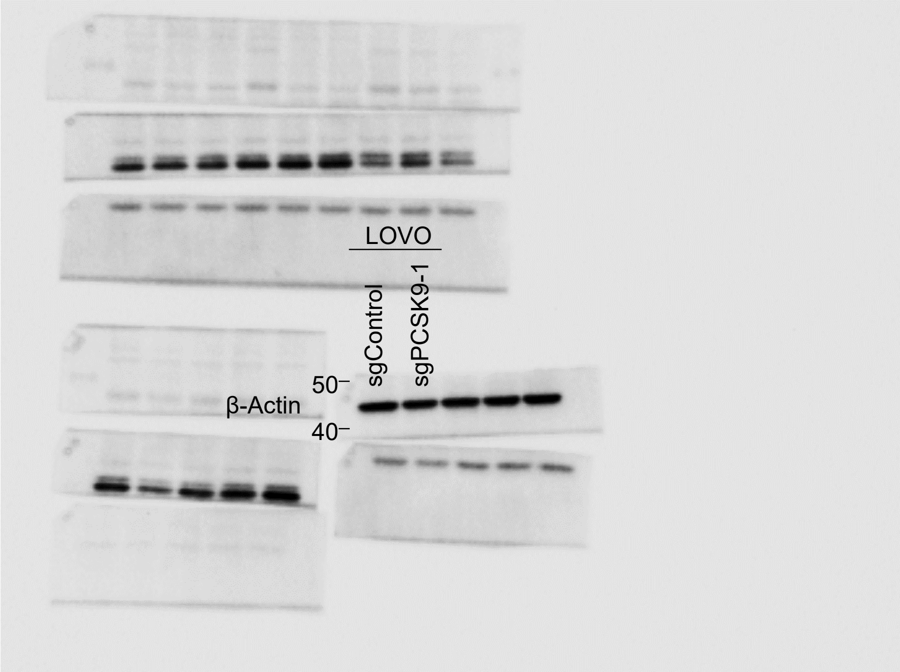

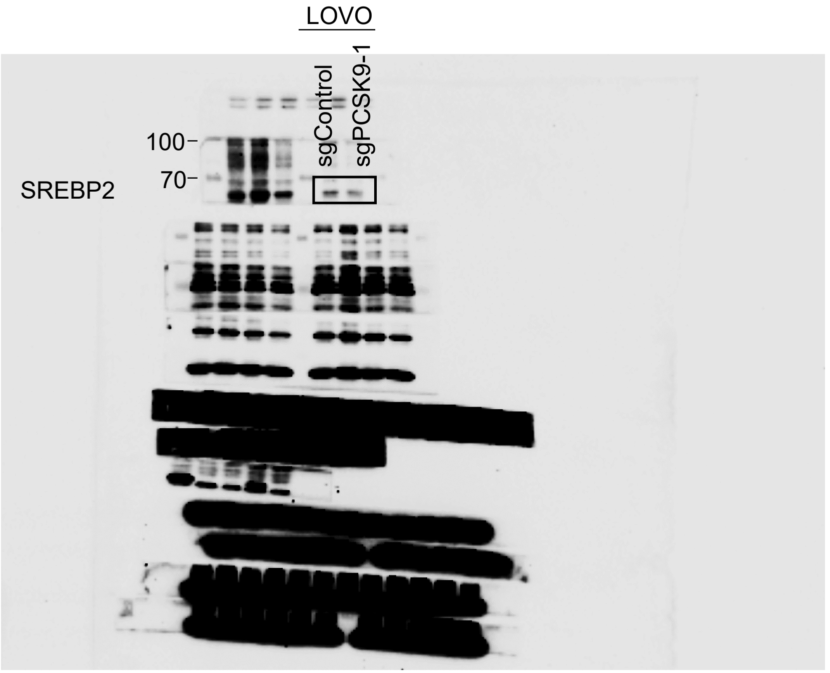

Figure 3C – SW1116

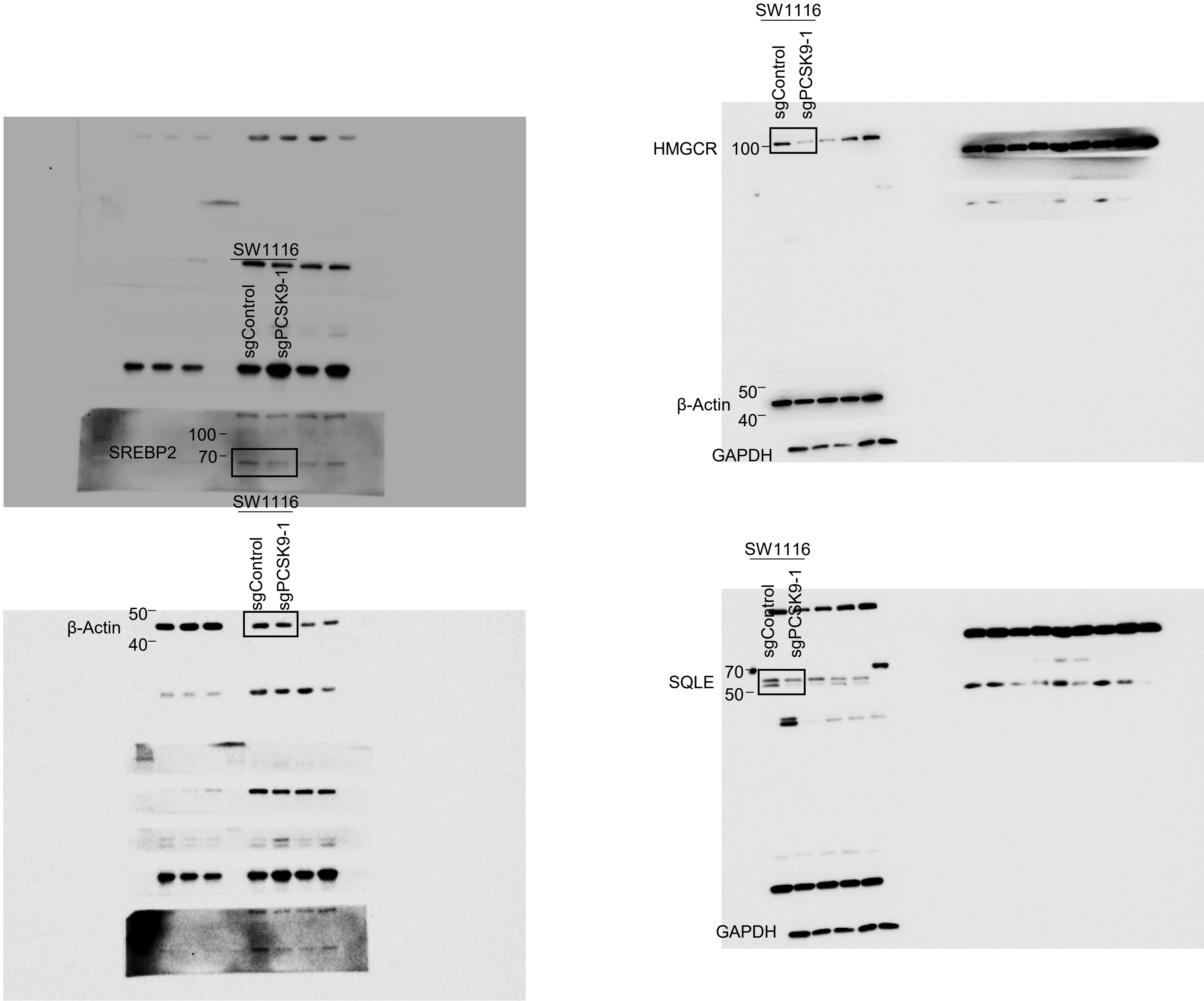

Figure 3C – DLD1

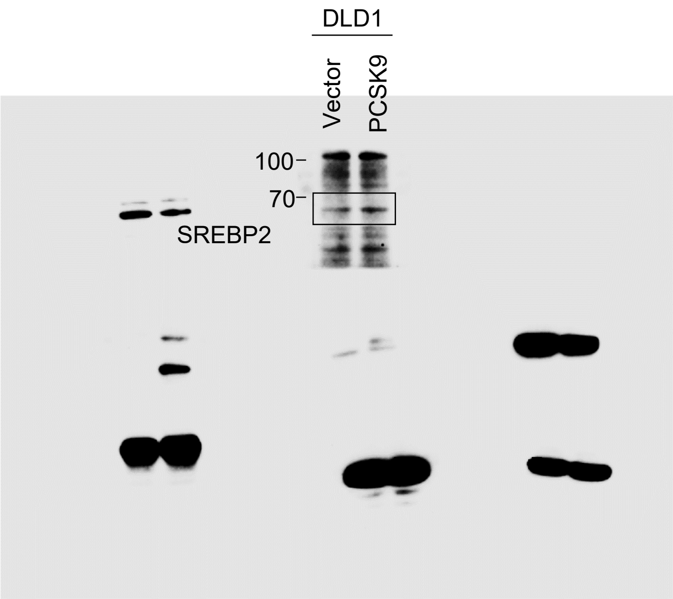

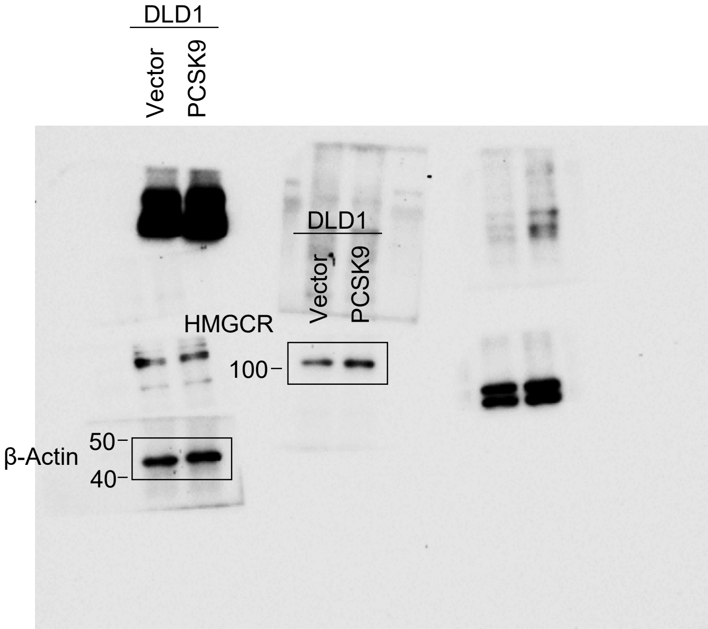

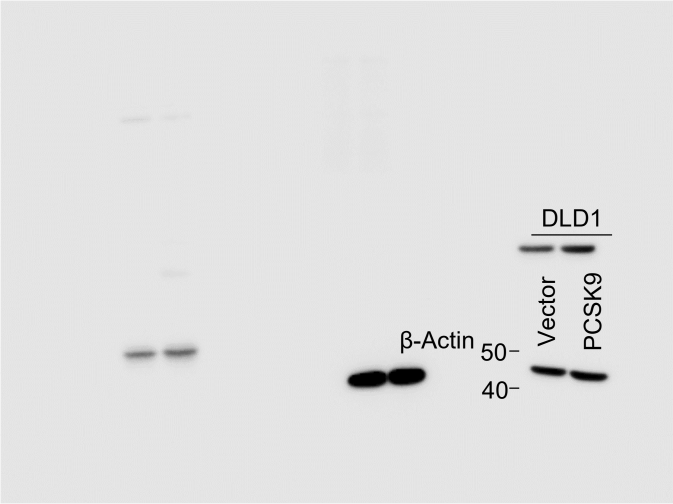

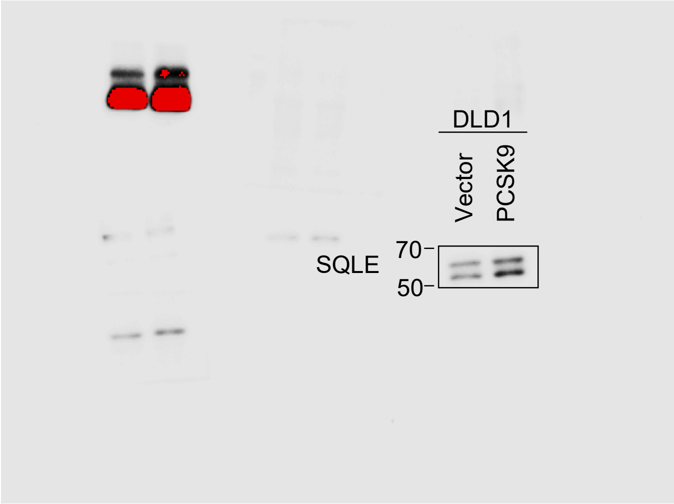

Figure 3D – upper panel

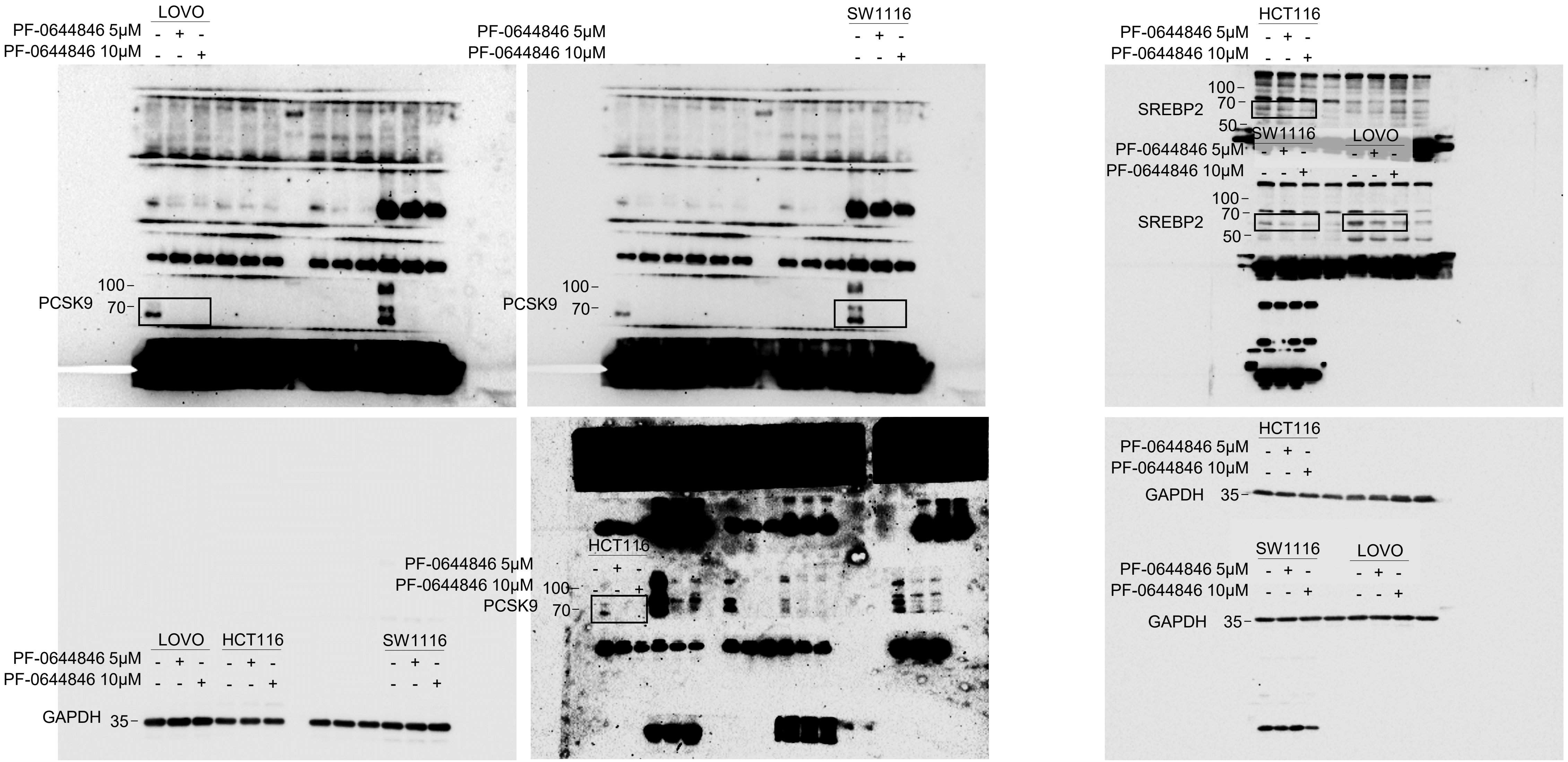

Figure 3D – upper panel

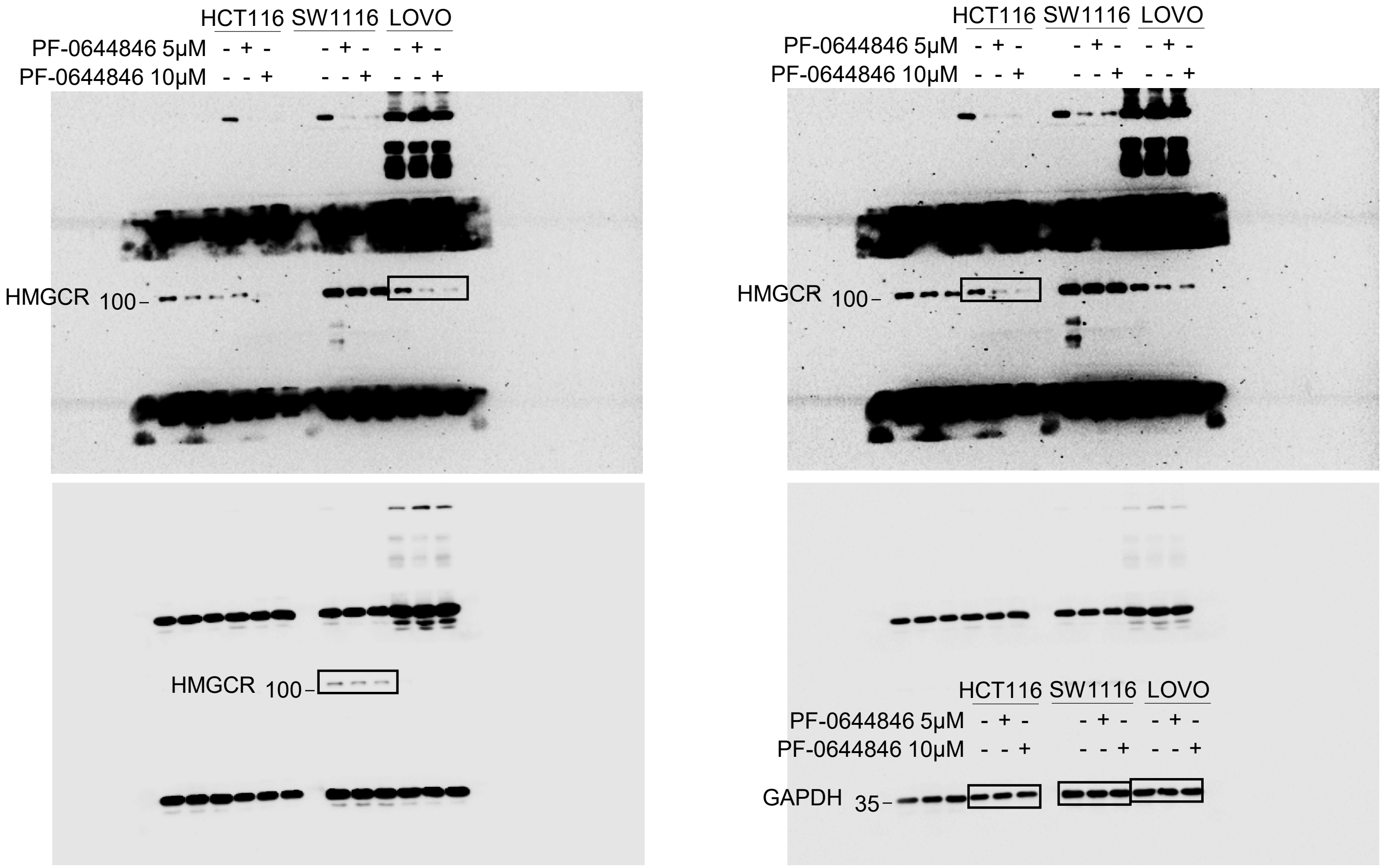

Figure 3D – lower panel

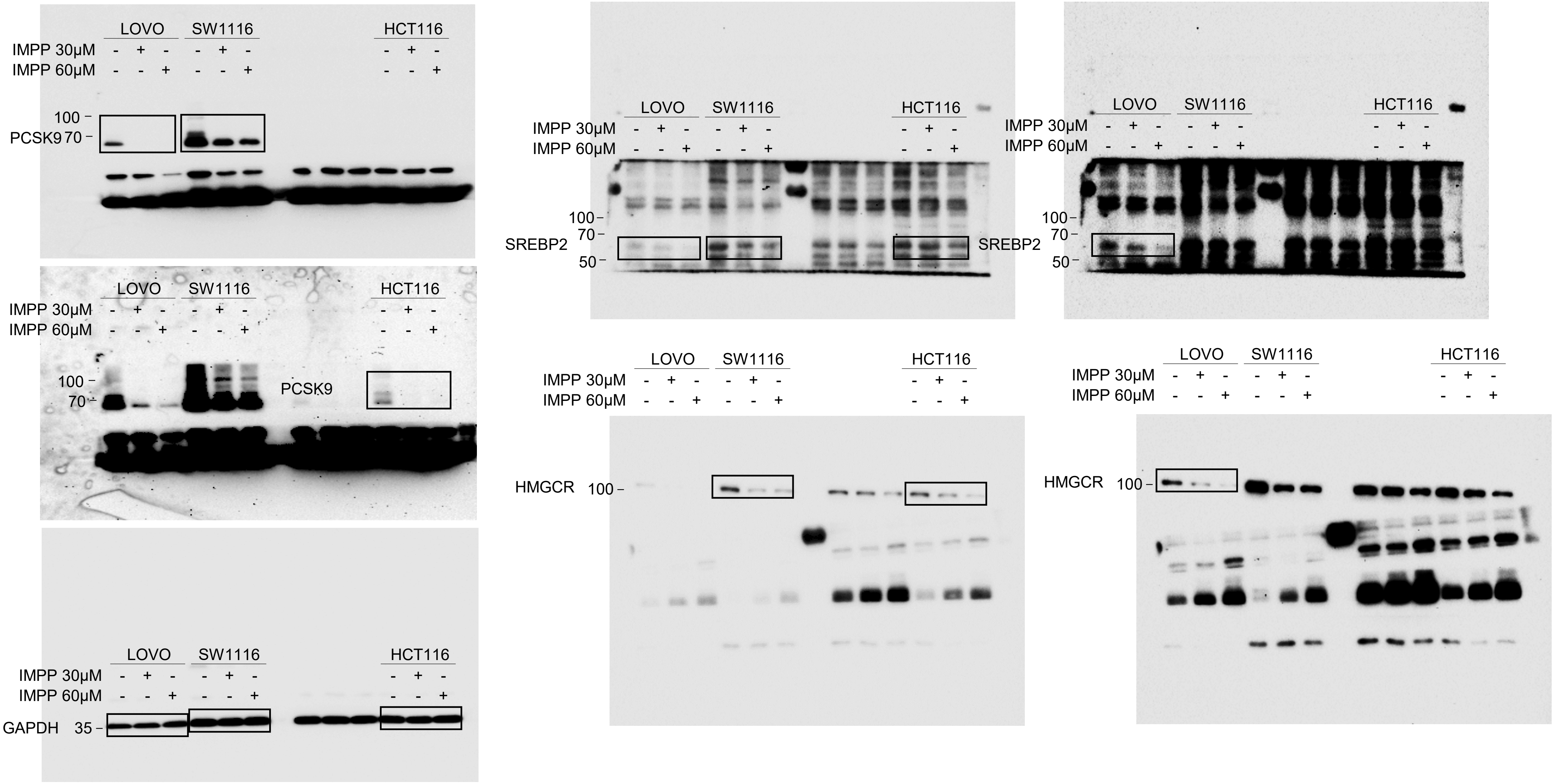

Figure 3G – lower panel

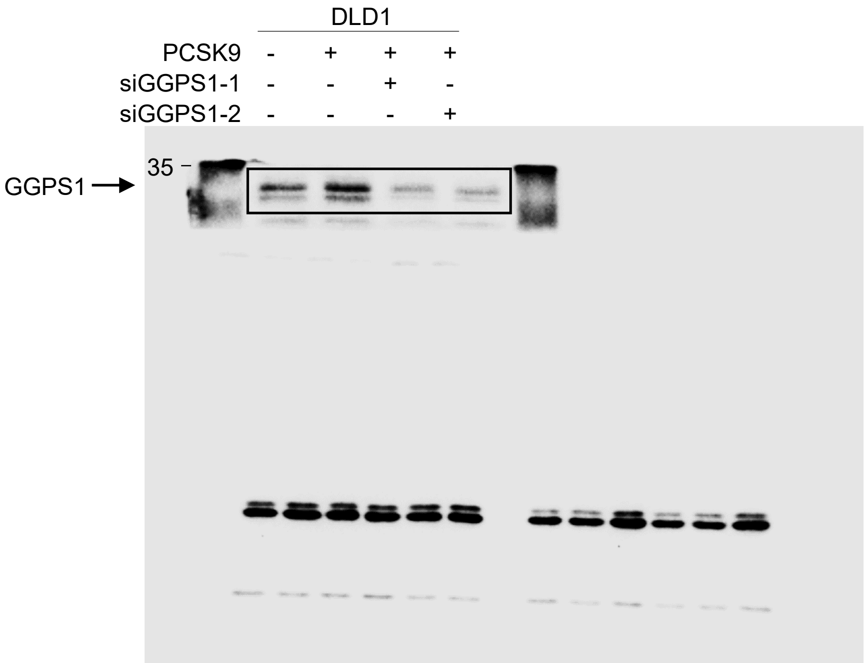

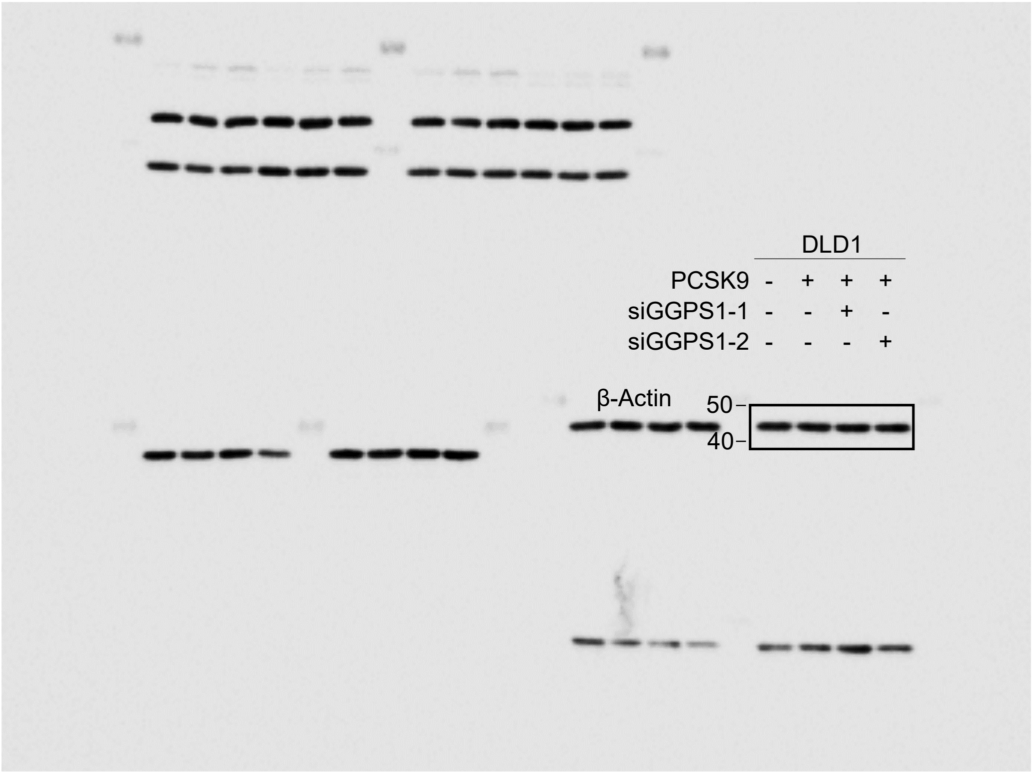

Figure 4B

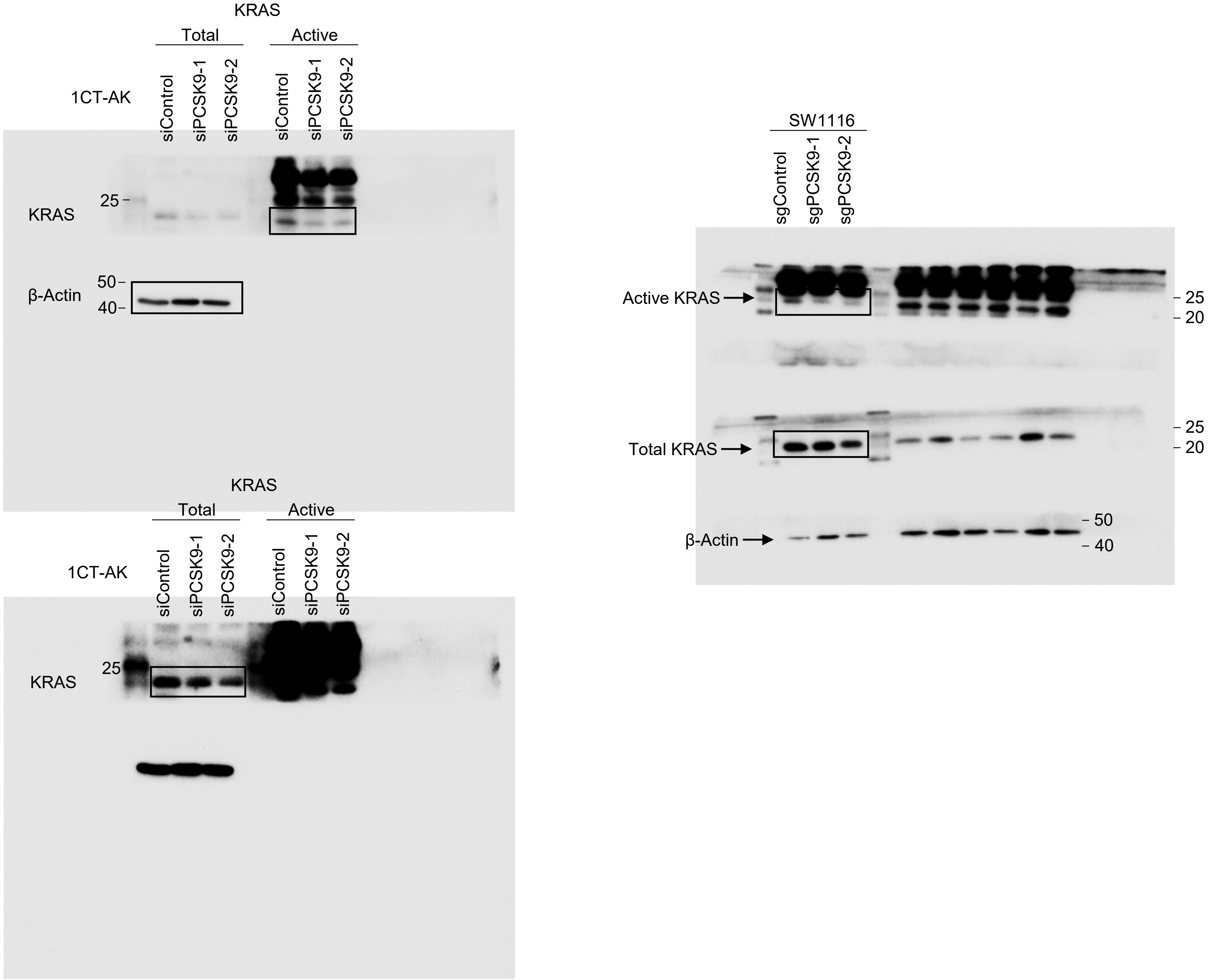
KRAS
Total
Active
1CT-AK
siControl
siPCSK9-1
siPCSK9-2
siControl
siPCSK9-1
siPCSK9-2
25
KRAS
50
β-Actin
40
KRAS
Total
Active
1CT-AK
siControl
siPCSK9-1
siPCSK9-2
siControl
siPCSK9-1
siPCSK9-2
25
KRAS
SW1116
sgControl
sgPCSK9-1
sgPCSK9-2
Active KRAS
25
20
Total KRAS
25
20
β-Actin
50
40

Figure 4C – left panel

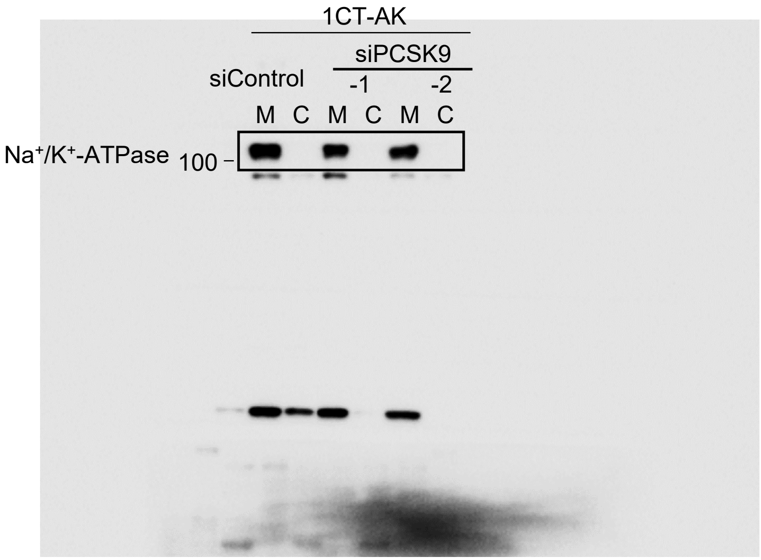

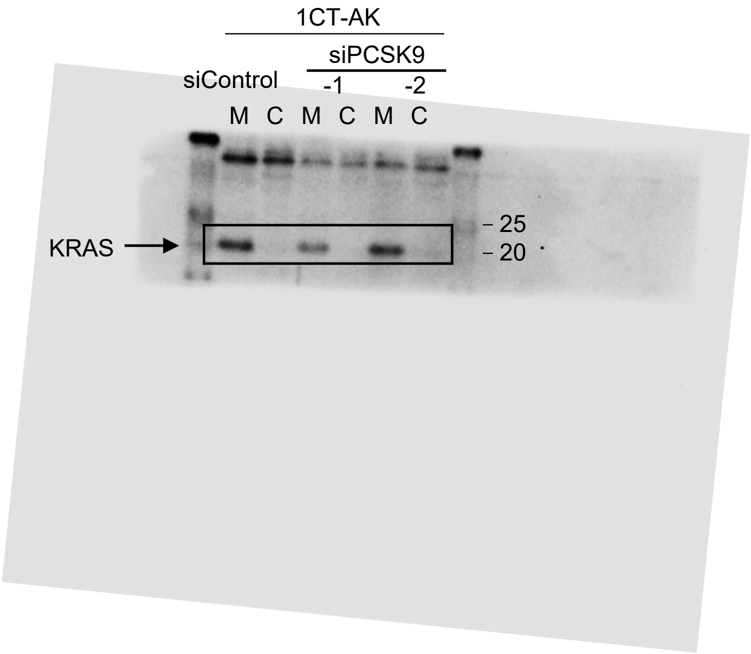

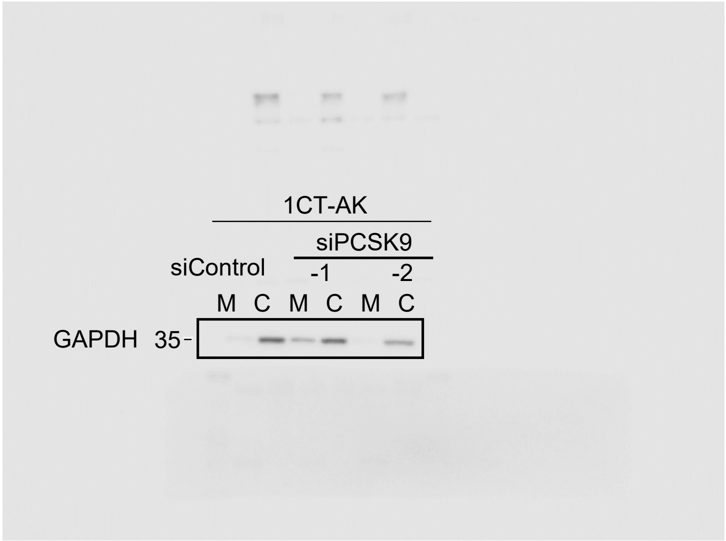

Figure 4C – right panel

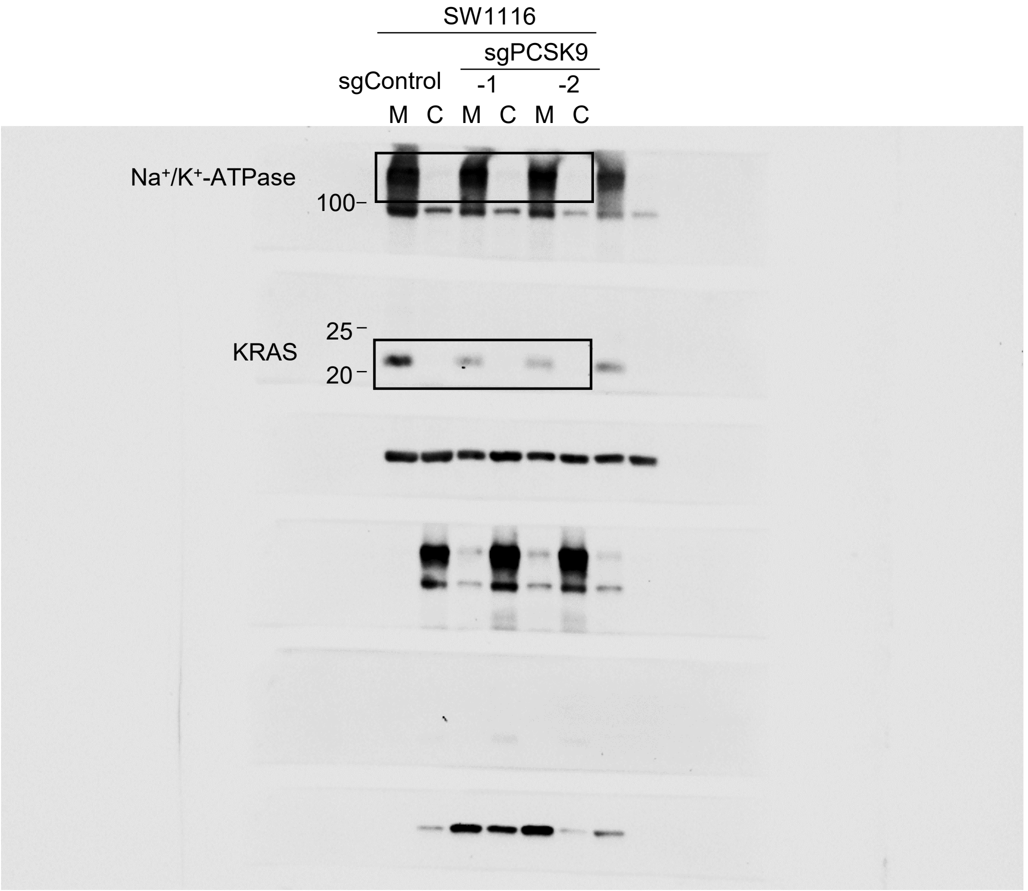

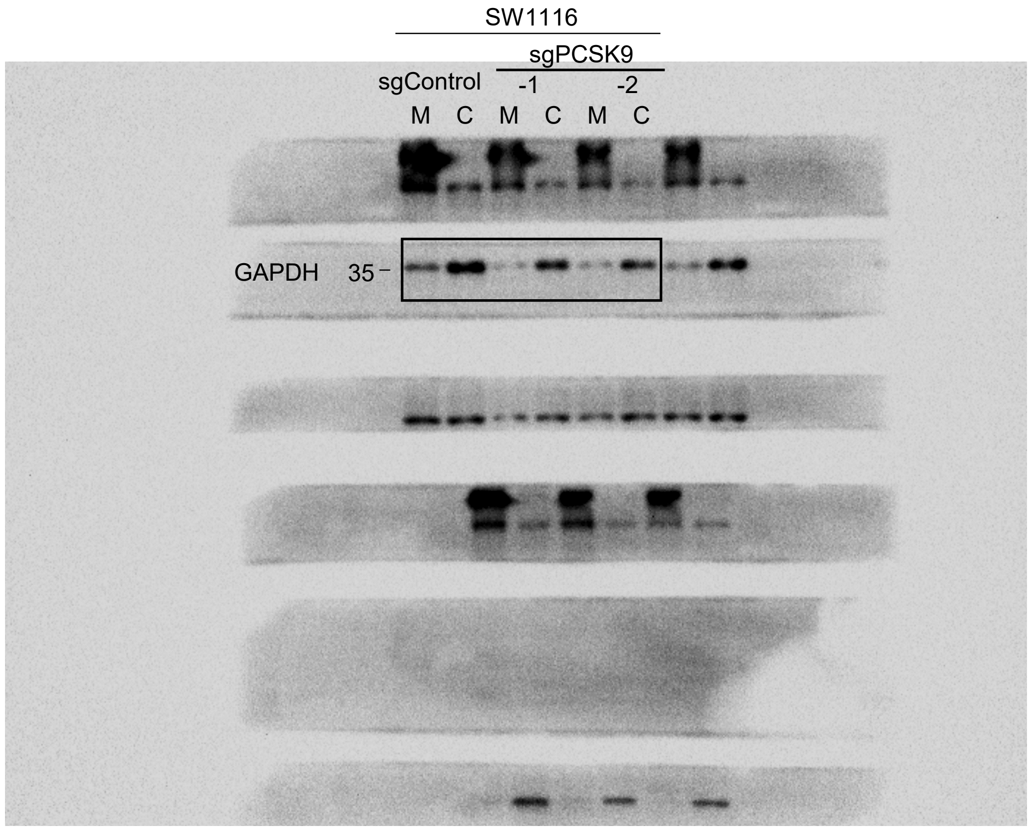

Figure 4D – left panel

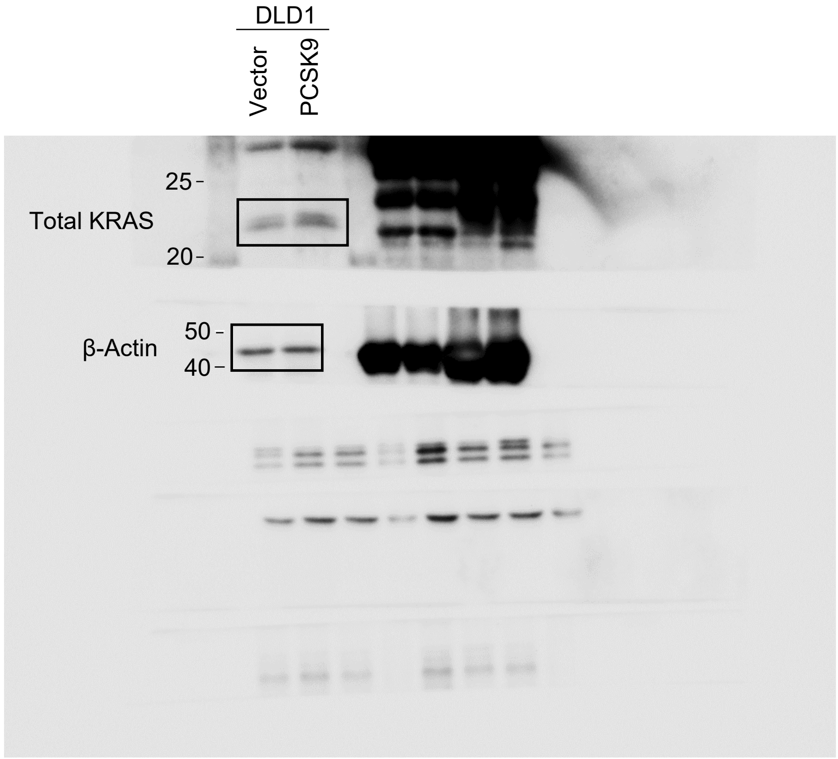

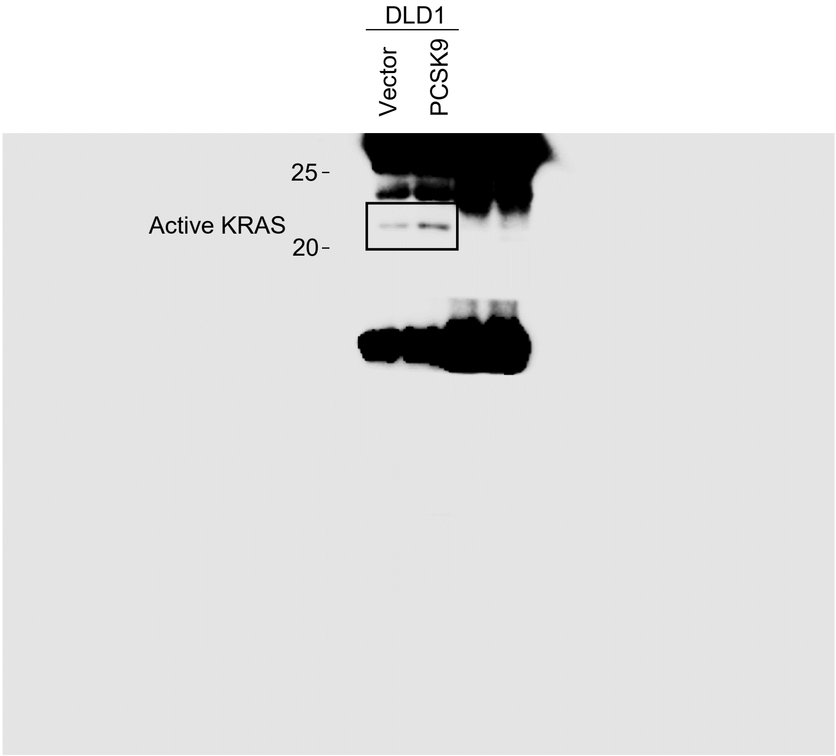

Figure 4D – right panel

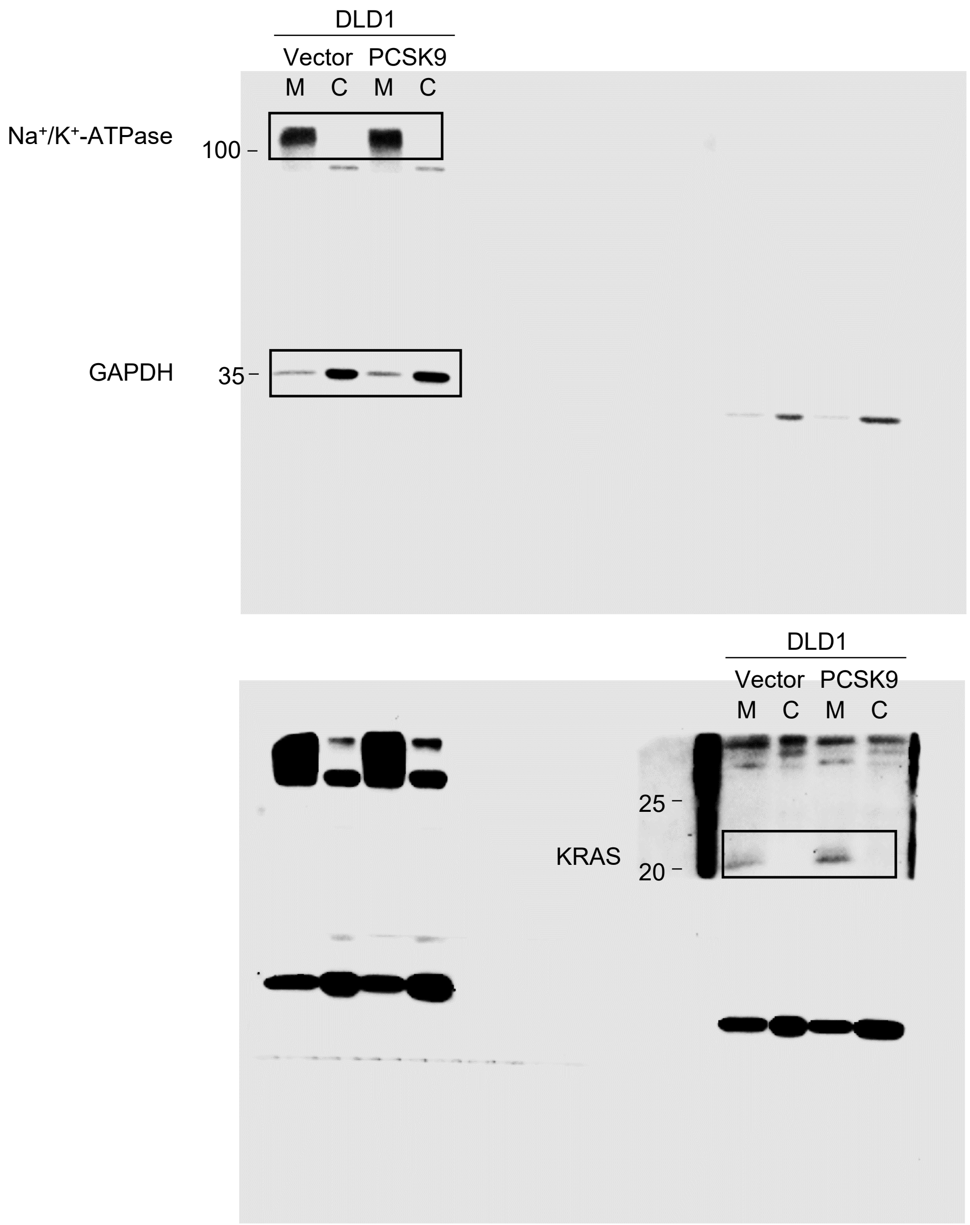

Figure 4E

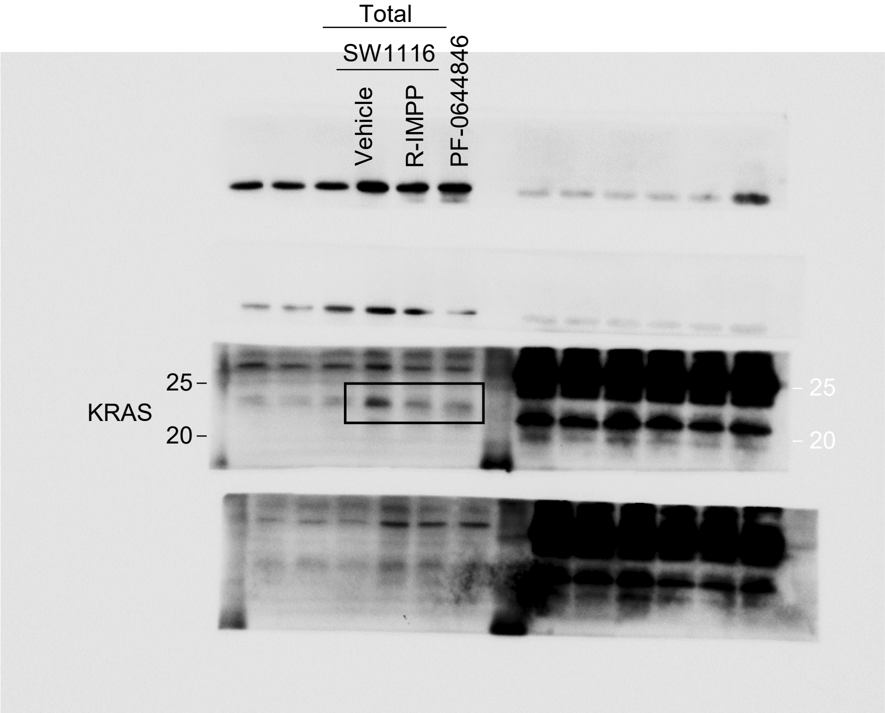
Total
SW1116
Vehicle
R-IMPP
PF-0644846
25
KRAS
20
25
20

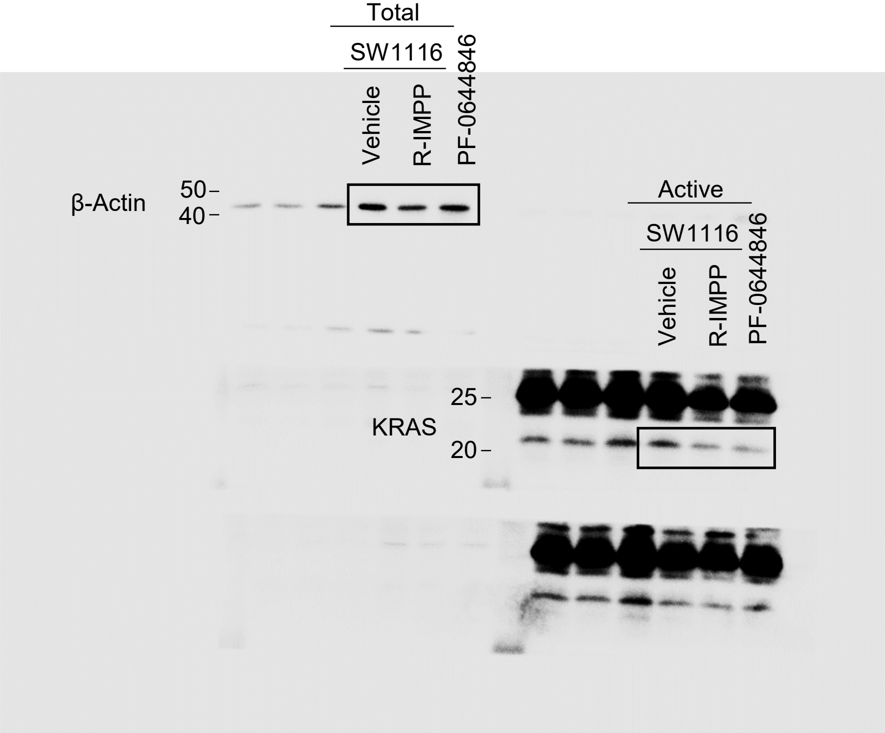
Total
SW1116
Vehicle
R-IMPP
PF-0644846
β-Actin
50
40
Active
SW1116
Vehicle
R-IMPP
PF-0644846
25
KRAS
20

Figure 4F – left panel

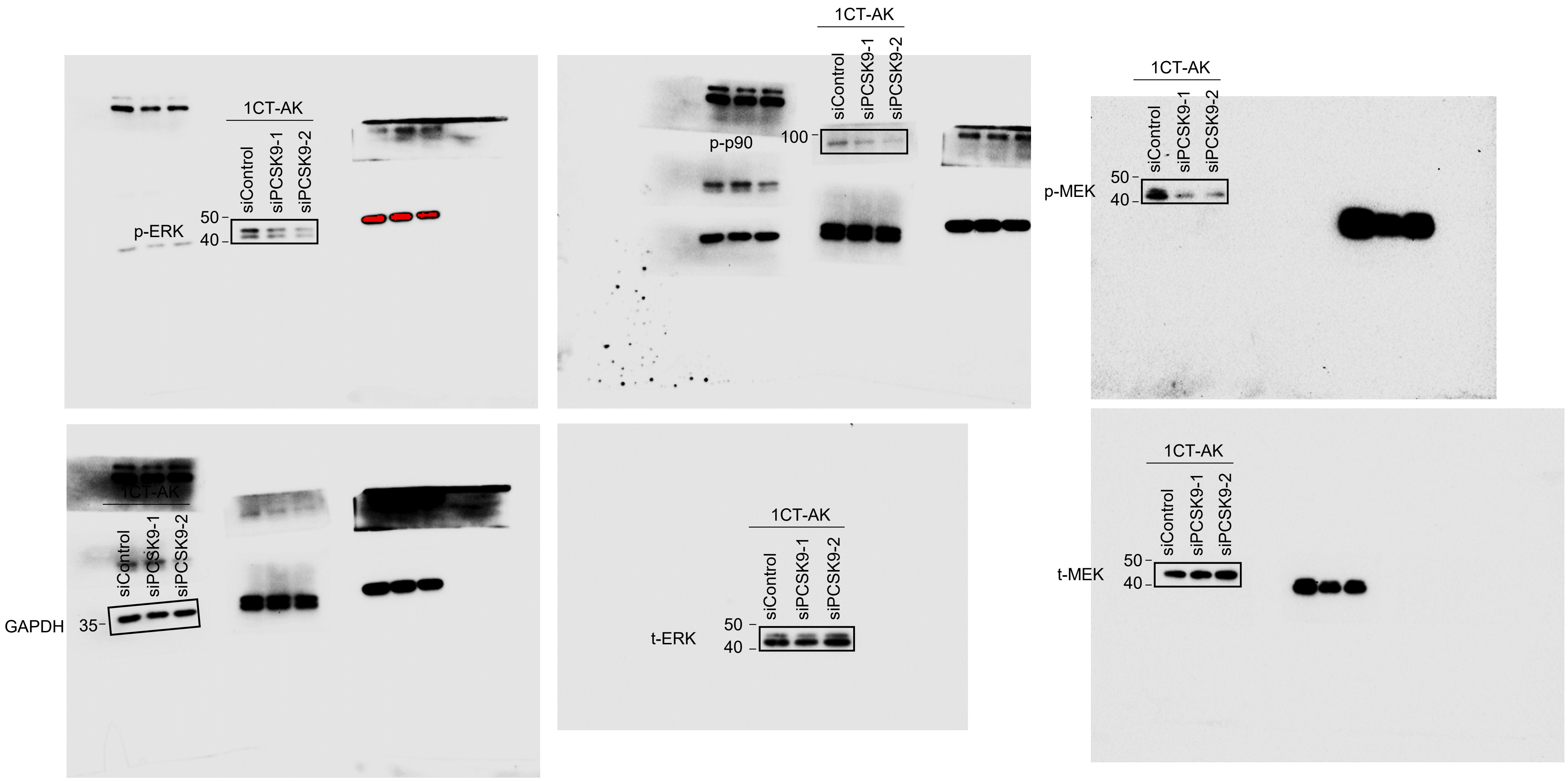

Figure 4F – right panel

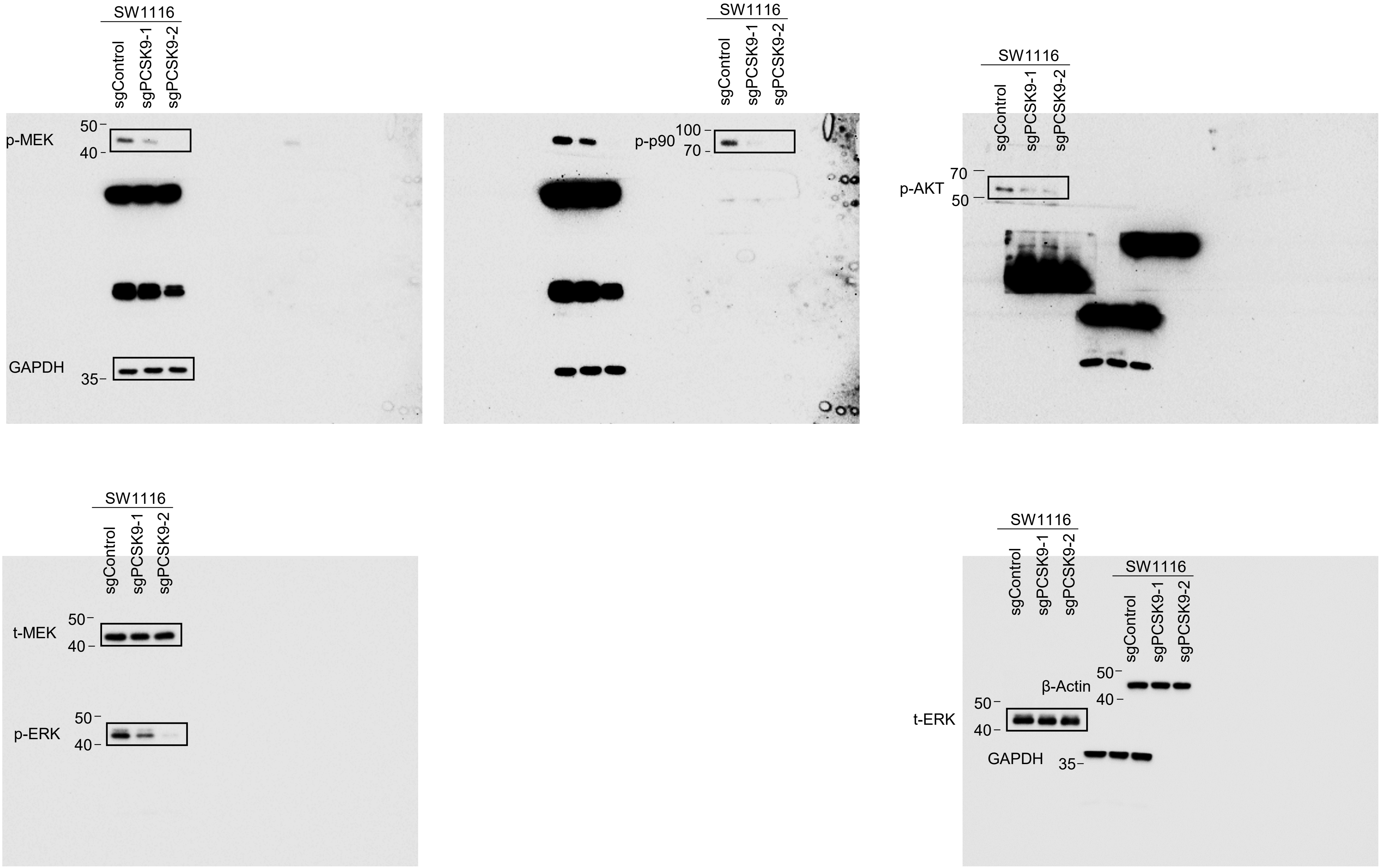

Figure 4G

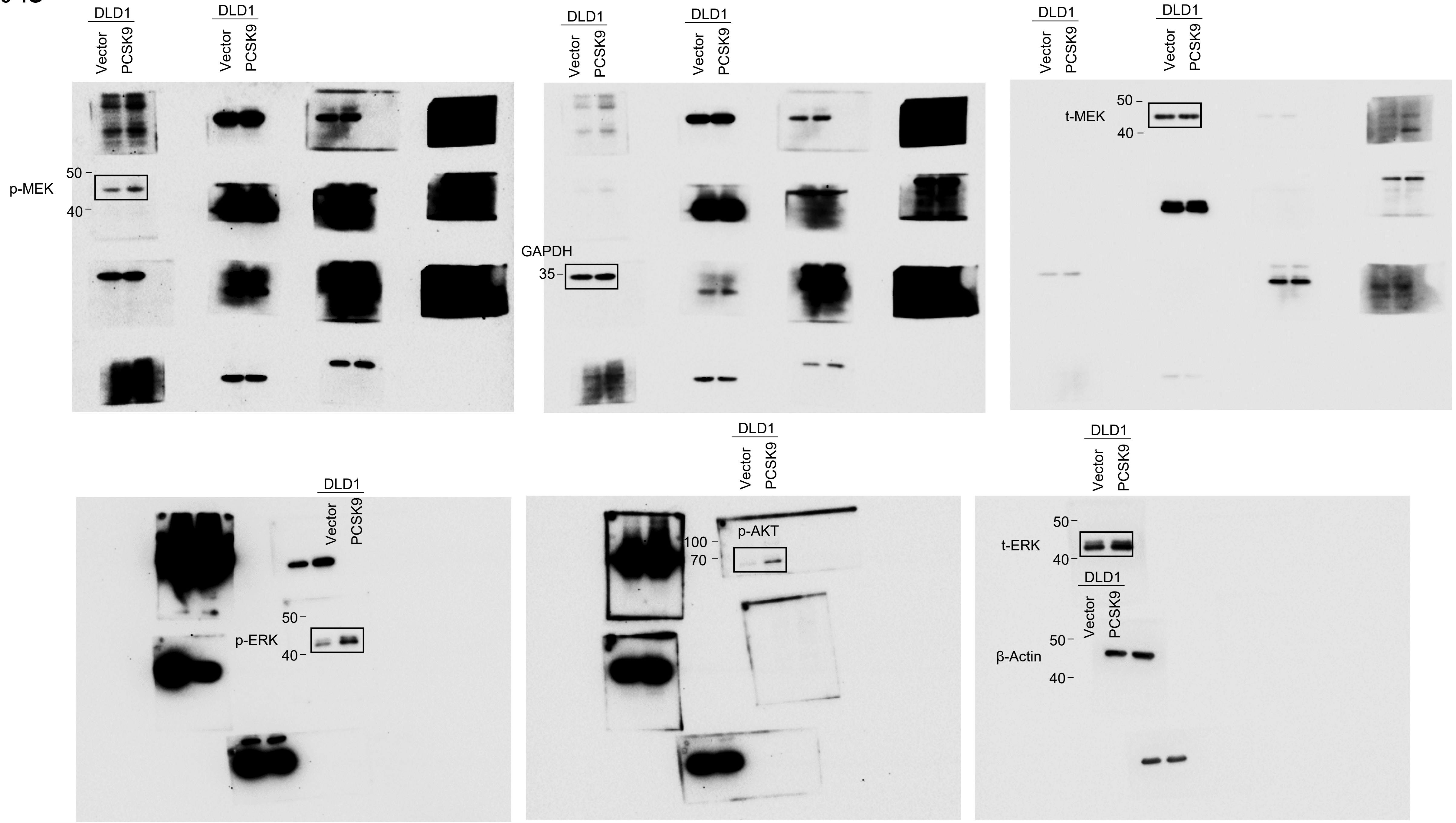
DLD1
Vector
PCSK9
p-MEK
50
40
GAPDH
35
t-MEK
p-ERK
p-AKT
100
70
t-ERK
β-Actin

Figure 4H – upper panel

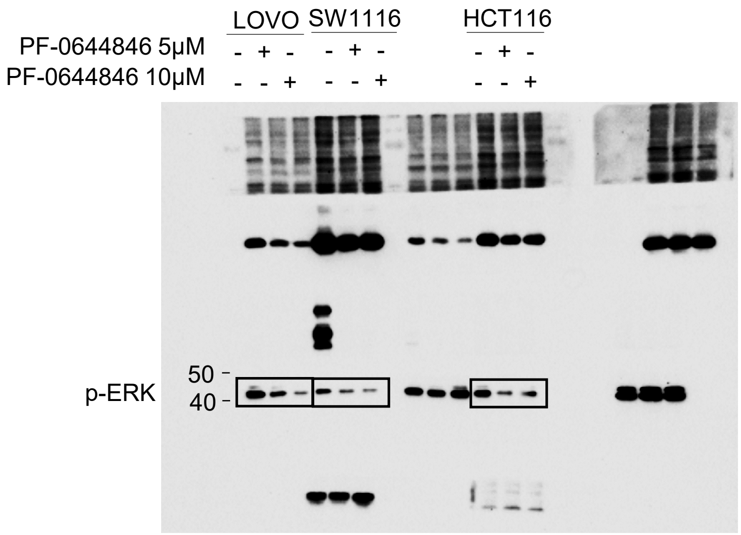

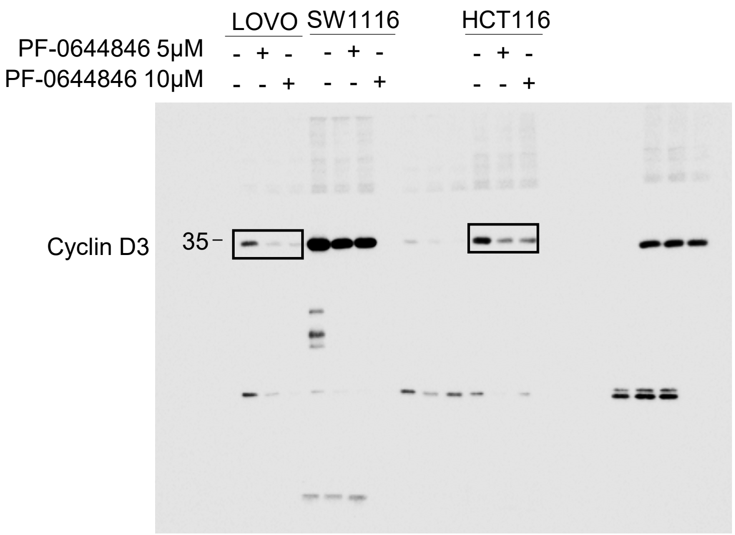

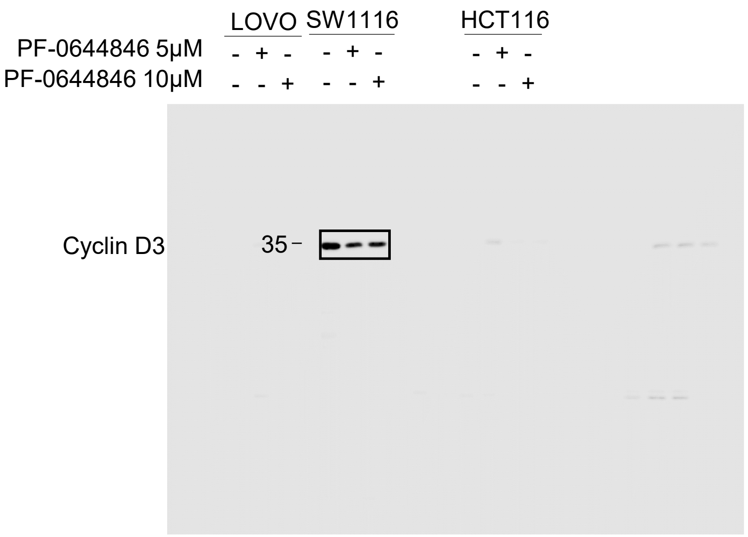

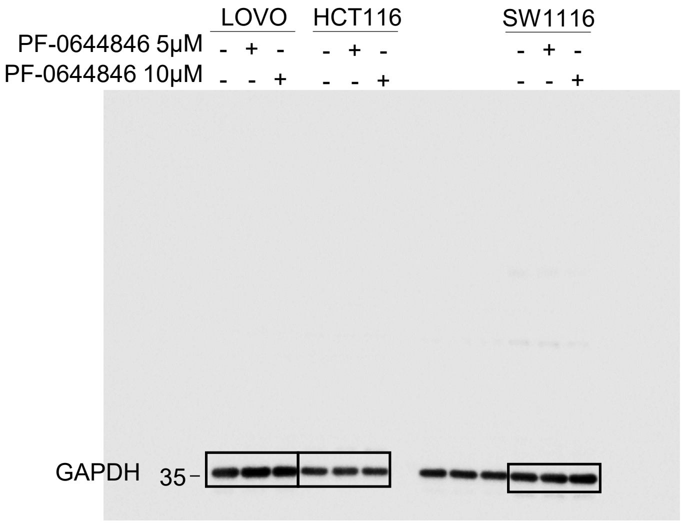

Figure 4H – lower panel

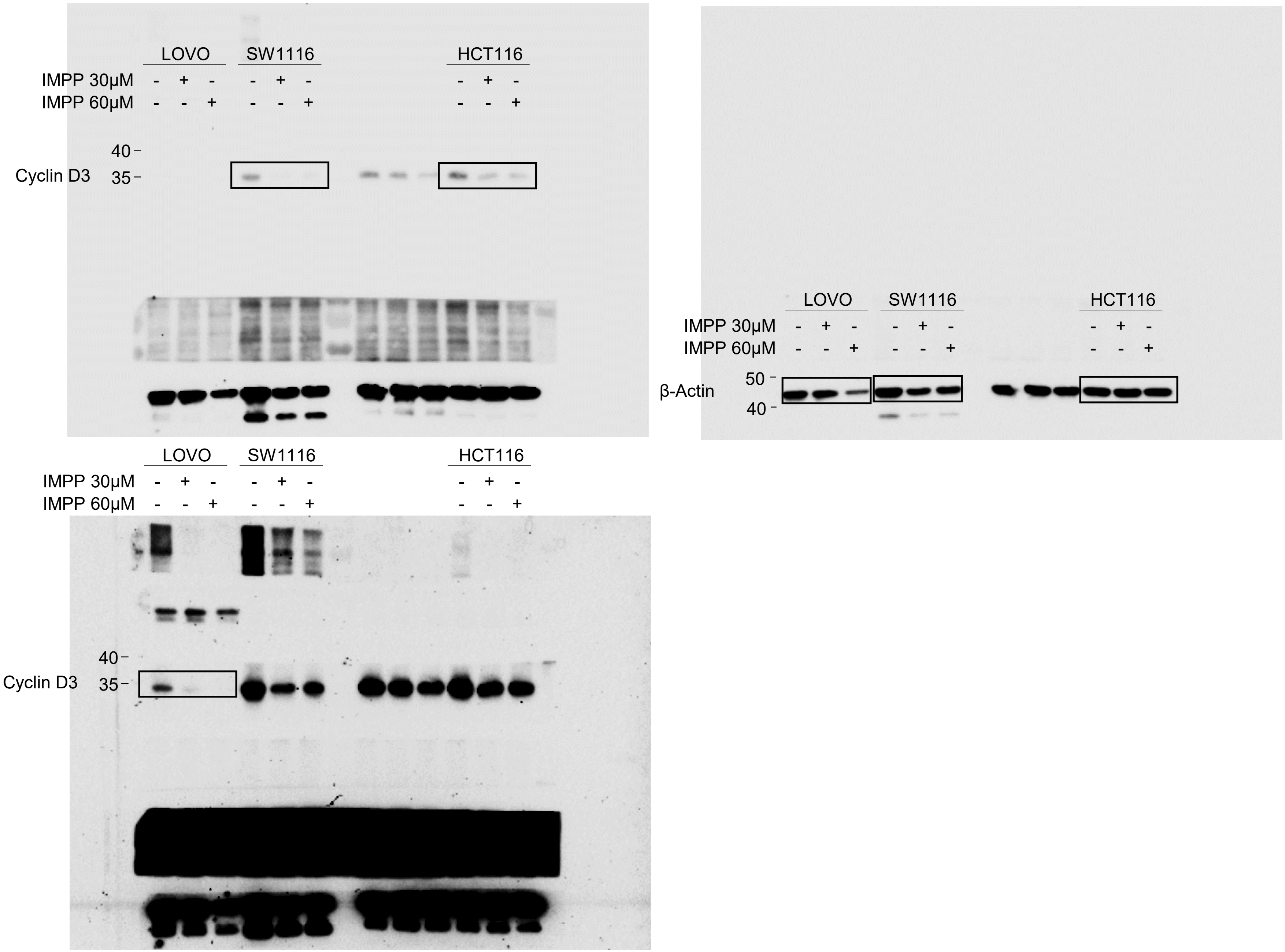

Figure 4I

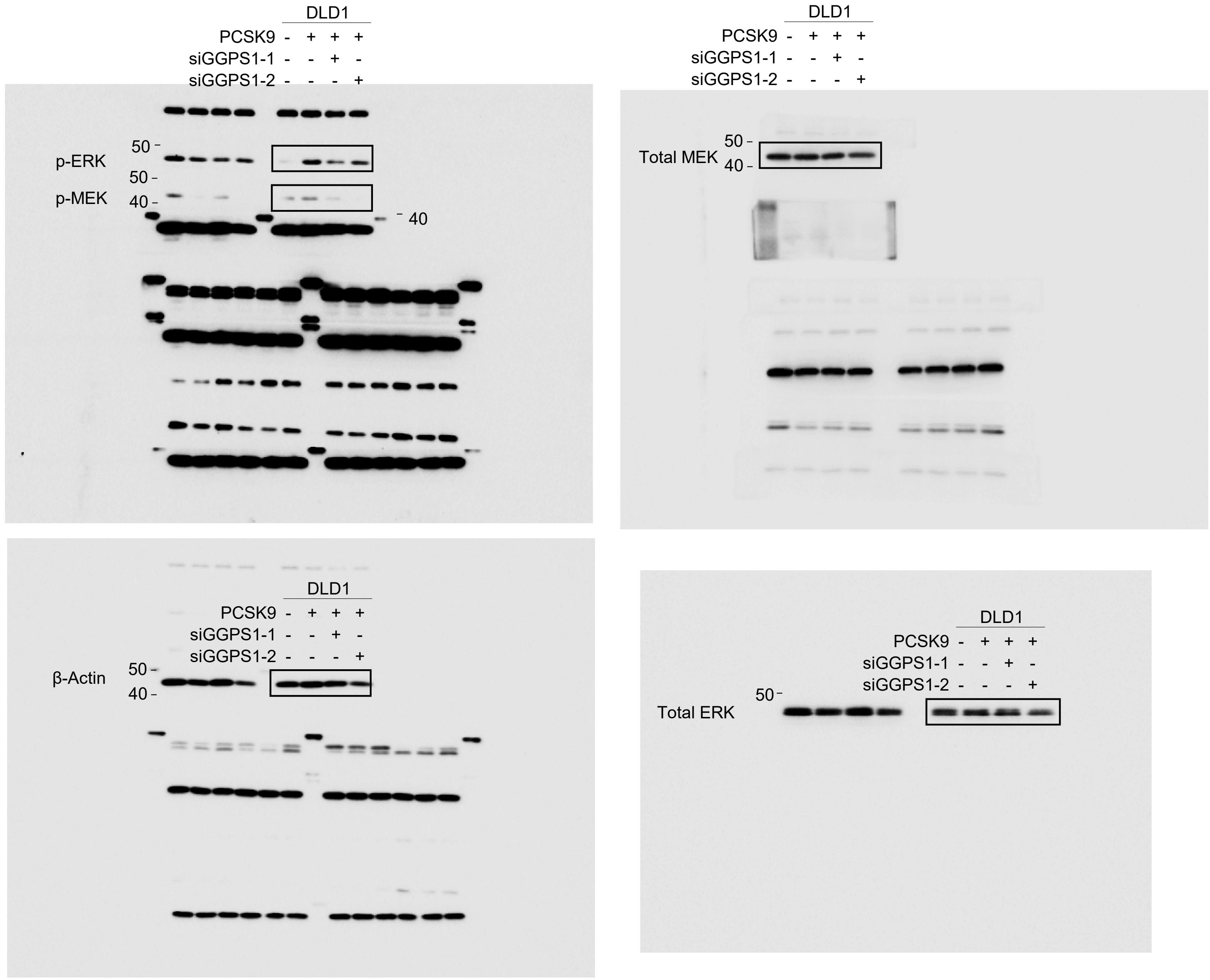
DLD1
PCSK9 - + + +
siGGPS1-1 - - + -
siGGPS1-2 - - - +
p-ERK
p-MEK
50
50
40
40
DLD1
PCSK9 - + + +
siGGPS1-1 - - + -
siGGPS1-2 - - - +
Total MEK
50
40
DLD1
PCSK9 - + + +
siGGPS1-1 - - + -
siGGPS1-2 - - - +
β-Actin
50
40
DLD1
PCSK9 - + + +
siGGPS1-1 - - + -
siGGPS1-2 - - - +
Total ERK
50

Figure 4J

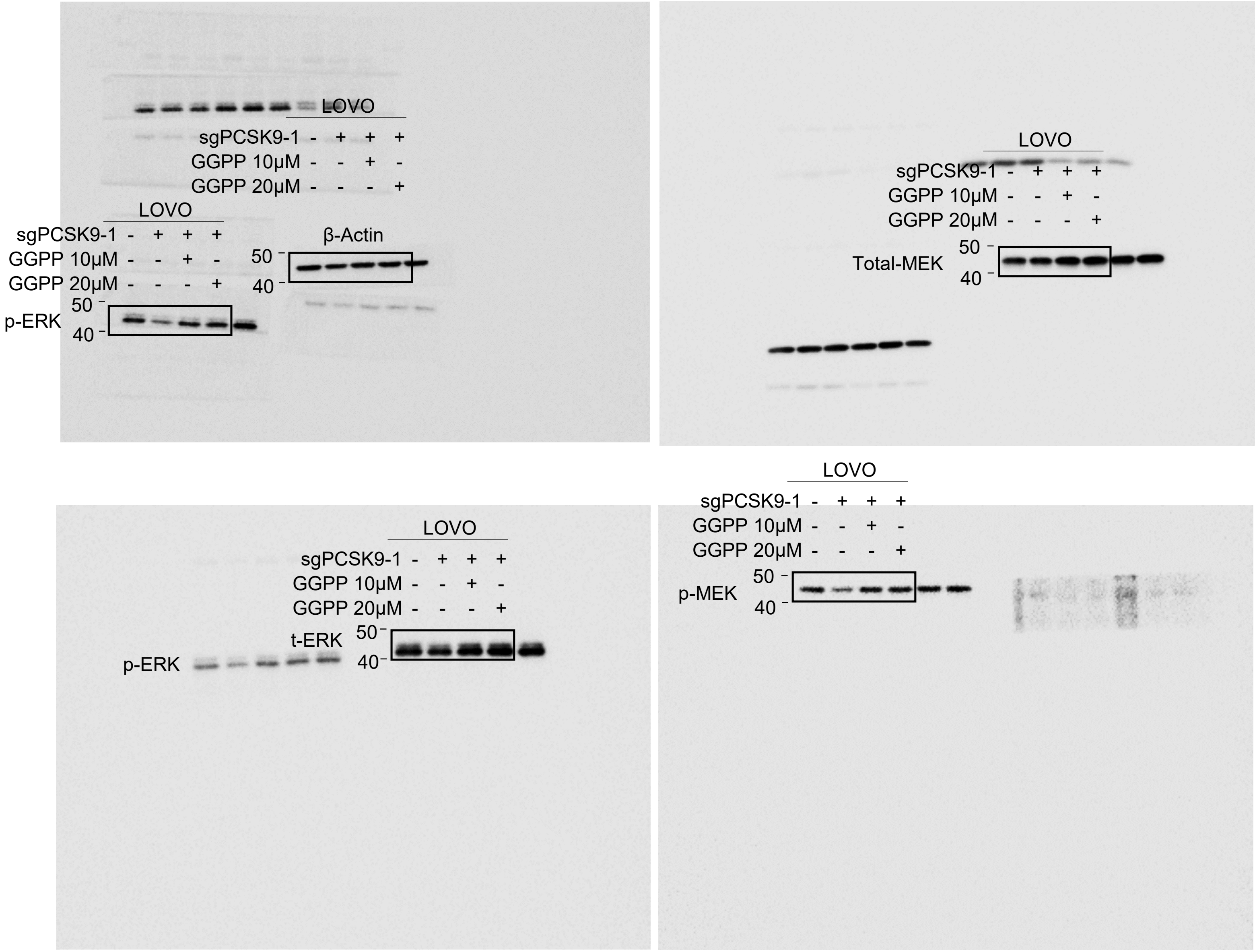
LOVO
sgPCSK9-1 - + + +
GGPP 10μM - - + -
GGPP 20μM - - - +
β-Actin
50
40
LOVO
sgPCSK9-1 - + + +
GGPP 10μM - - + -
GGPP 20μM - - - +
p-ERK
50
40
LOVO
sgPCSK9-1 - + + +
GGPP 10μM - - + -
GGPP 20μM - - - +
Total-MEK
50
40
LOVO
sgPCSK9-1 - + + +
GGPP 10μM - - + -
GGPP 20μM - - - +
t-ERK
p-ERK
50
40
LOVO
sgPCSK9-1 - + + +
GGPP 10μM - - + -
GGPP 20μM - - - +
p-MEK
50
40

Figure 5E – left panel

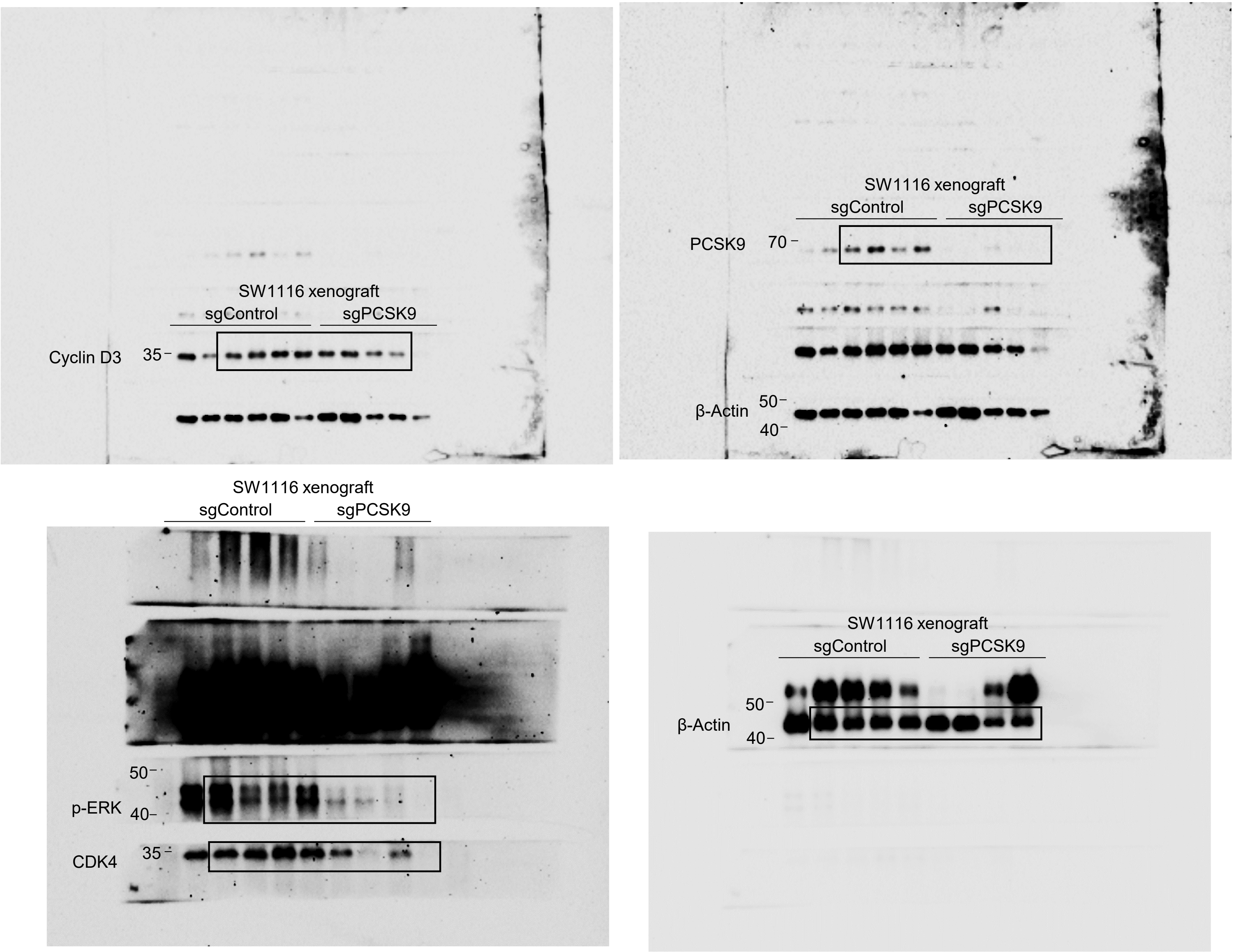

Figure 5E – right panel

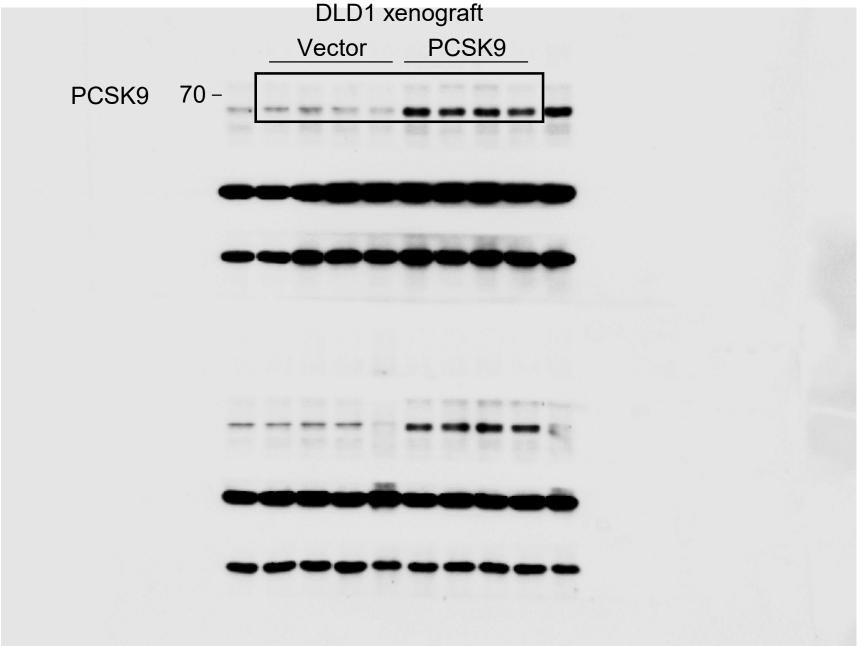

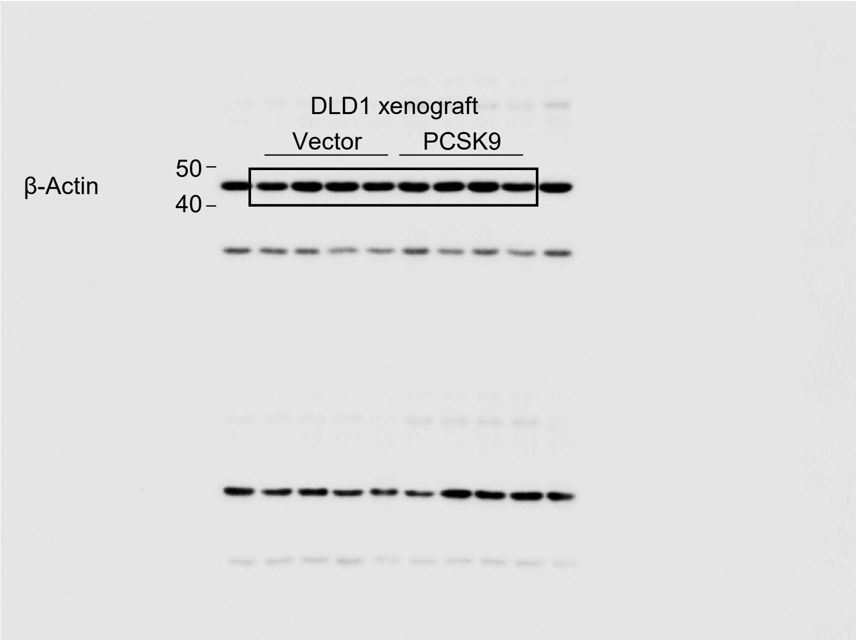

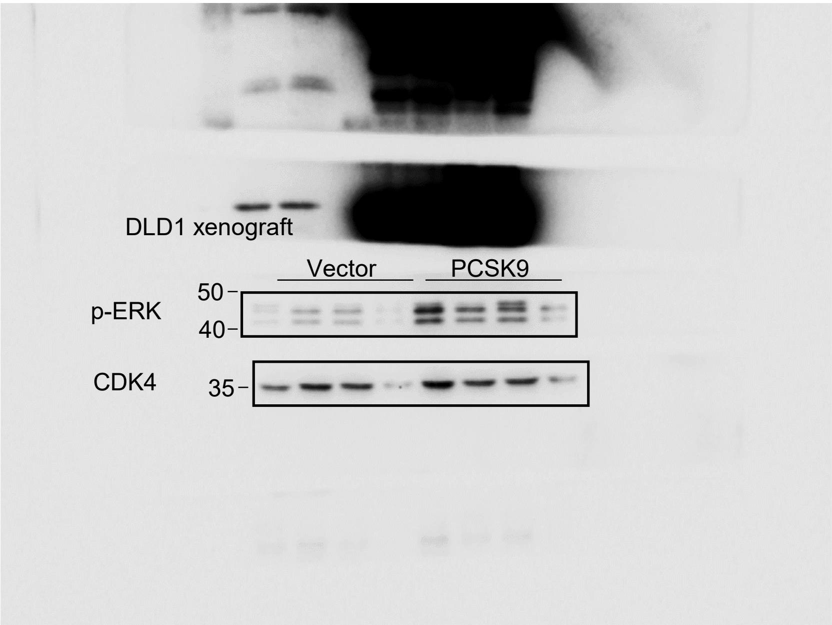

Figure 5J

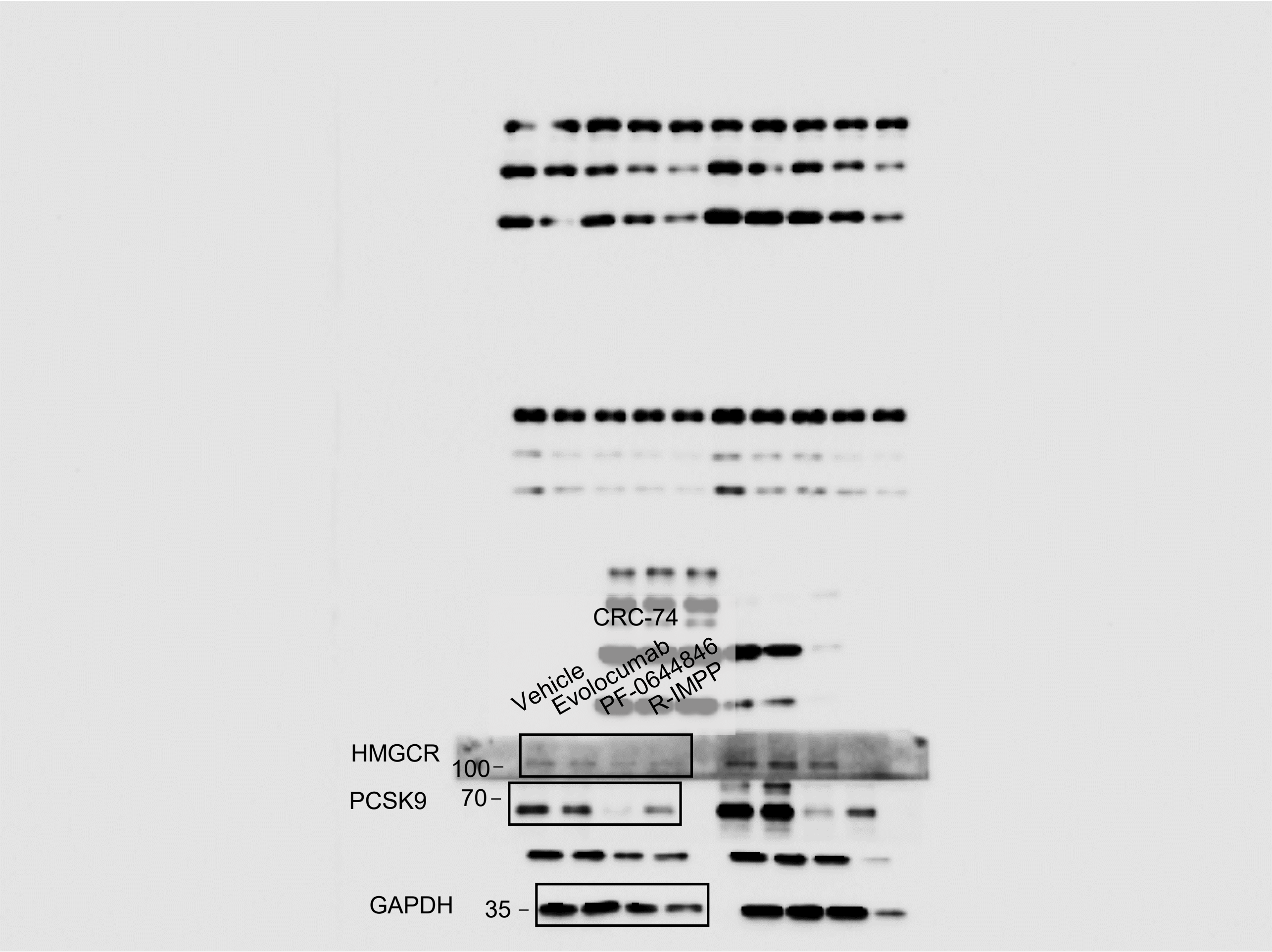
CRC-74
Vehicle
Evolocumab
PF-0644846
R-IMPP
HMGCR
100
PCSK9
70
GAPDH
35

Figure 6E

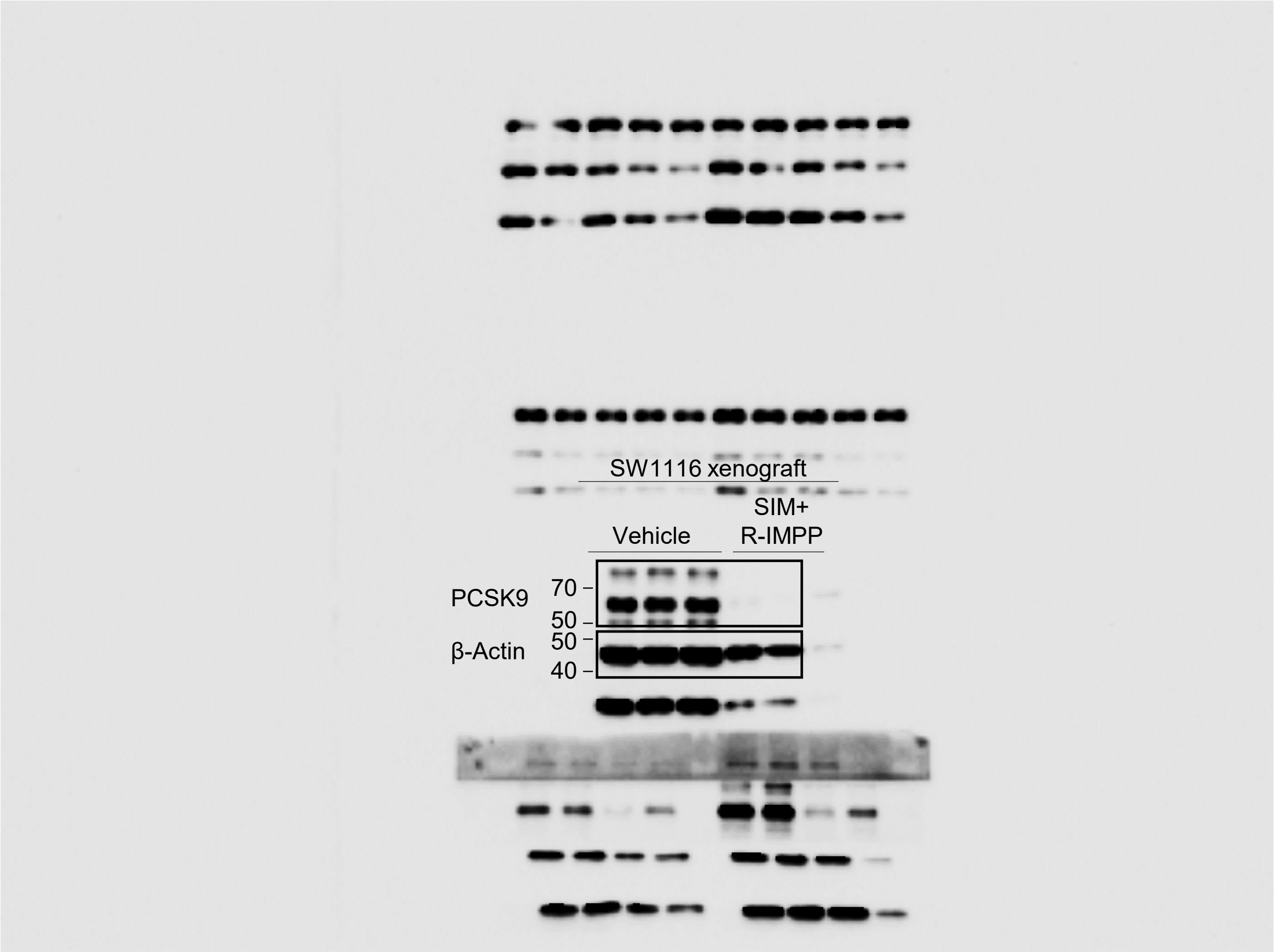

Figure 6G

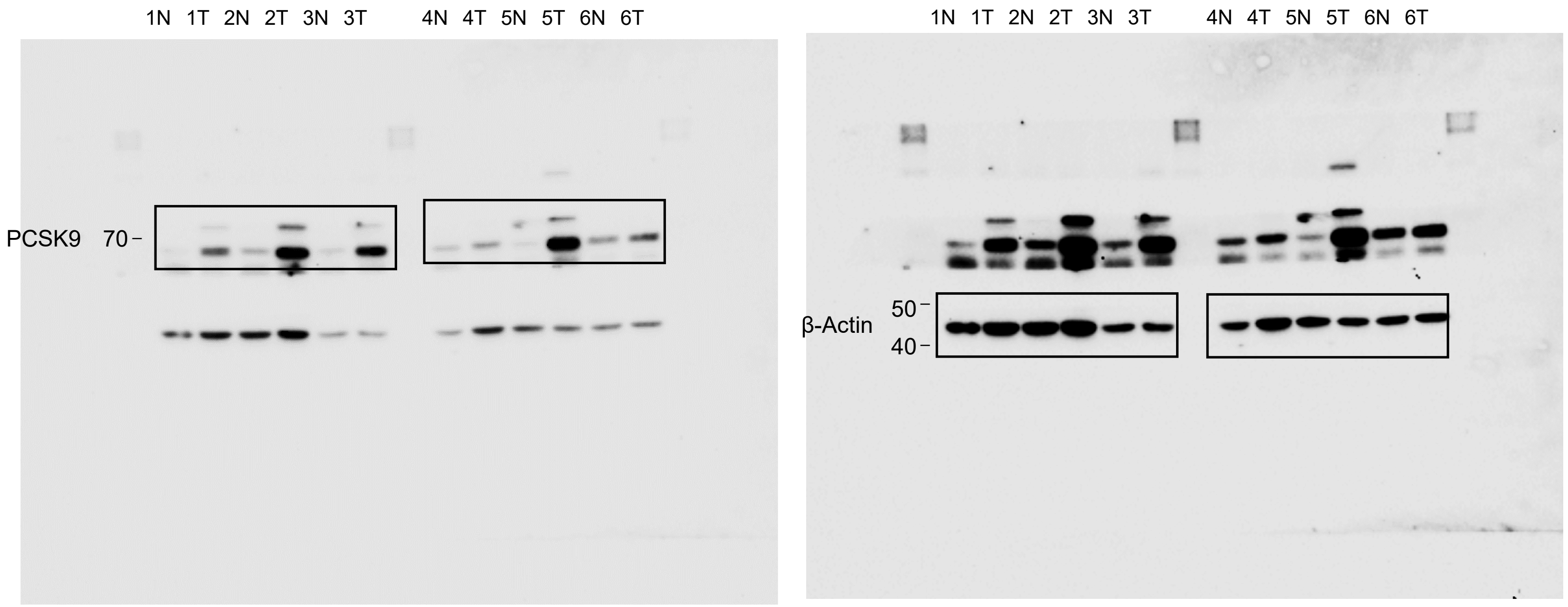
1N 1T 2N 2T 3N 3T 4N 4T 5N 5T 6N 6T
PCSK9
70
1N 1T 2N 2T 3N 3T 4N 4T 5N 5T 6N 6T
β-Actin
50
40

Figure S4A

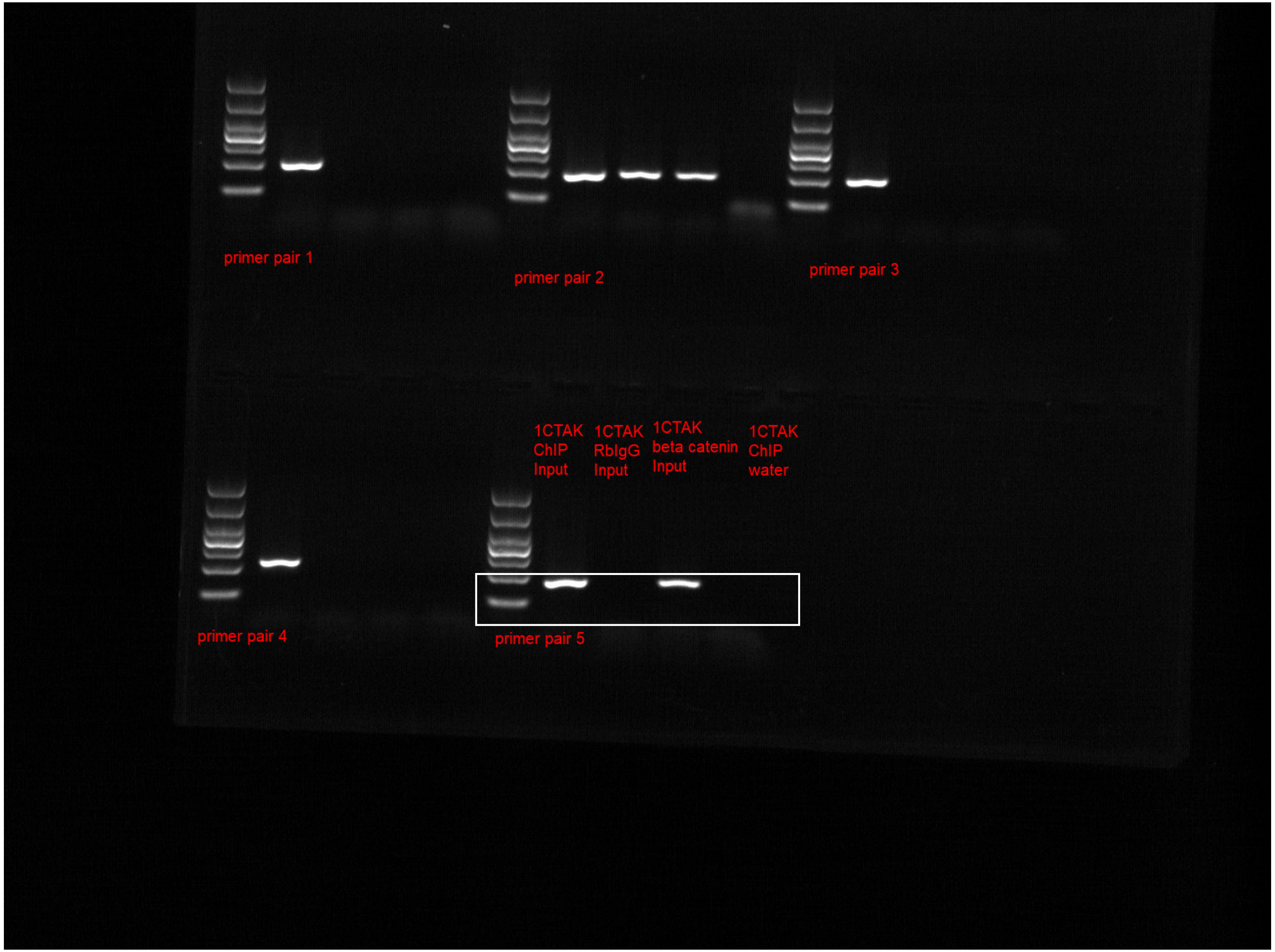
primer pair 1
primer pair 2
primer pair 3
1CTAK ChIP Input
1CTAK RbIgG Input
1CTAK beta catenin Input
1CTAK ChIP water
primer pair 4
primer pair 5

Figure S4C

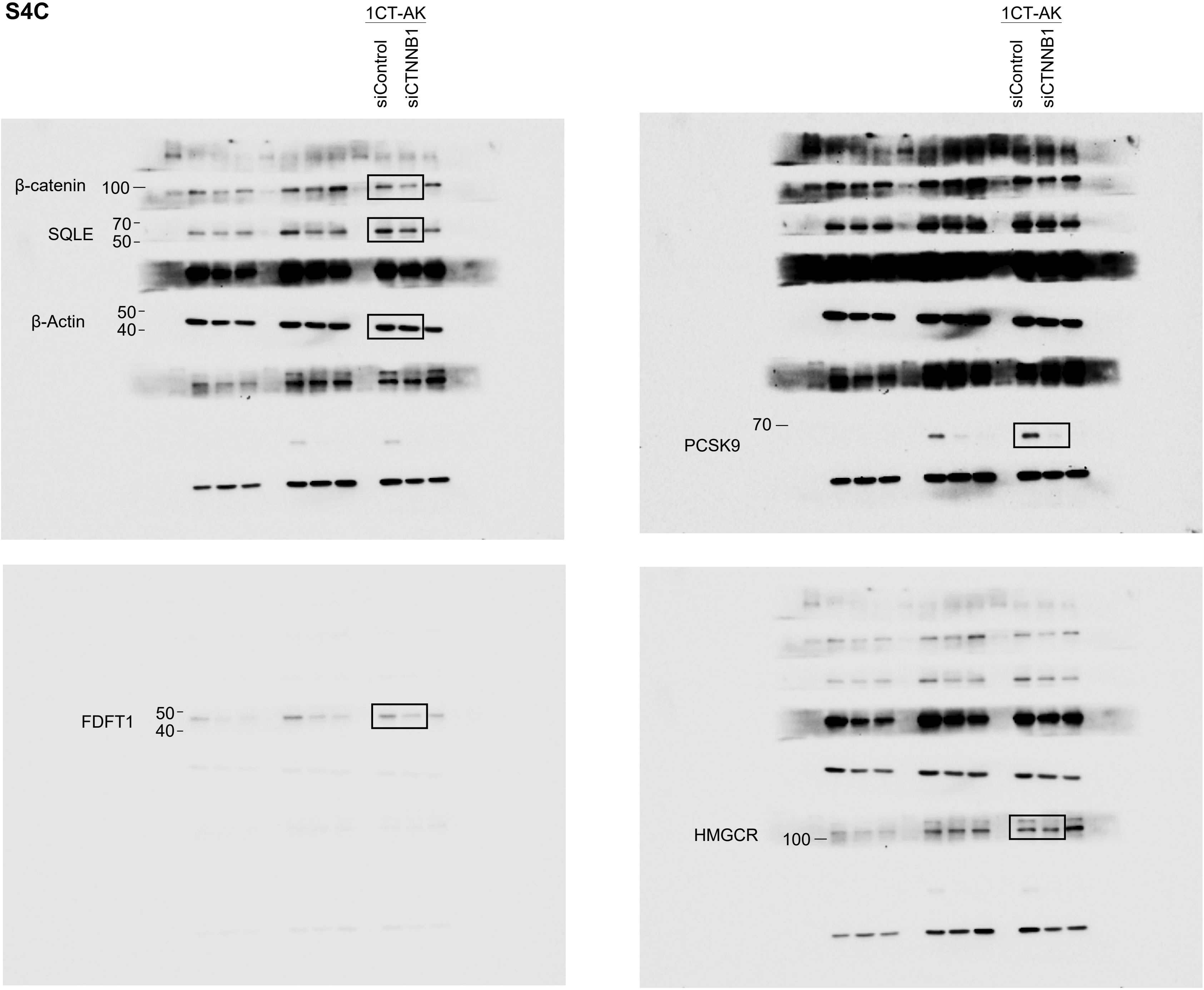
1CT-AK
siControl
siCTNNB1
β-catenin
100
SQLE
70
50
β-Actin
50
40
FDFT1
50
40
1CT-AK
siControl
siCTNNB1
70
PCSK9
HMGCR
100

Figure S5A

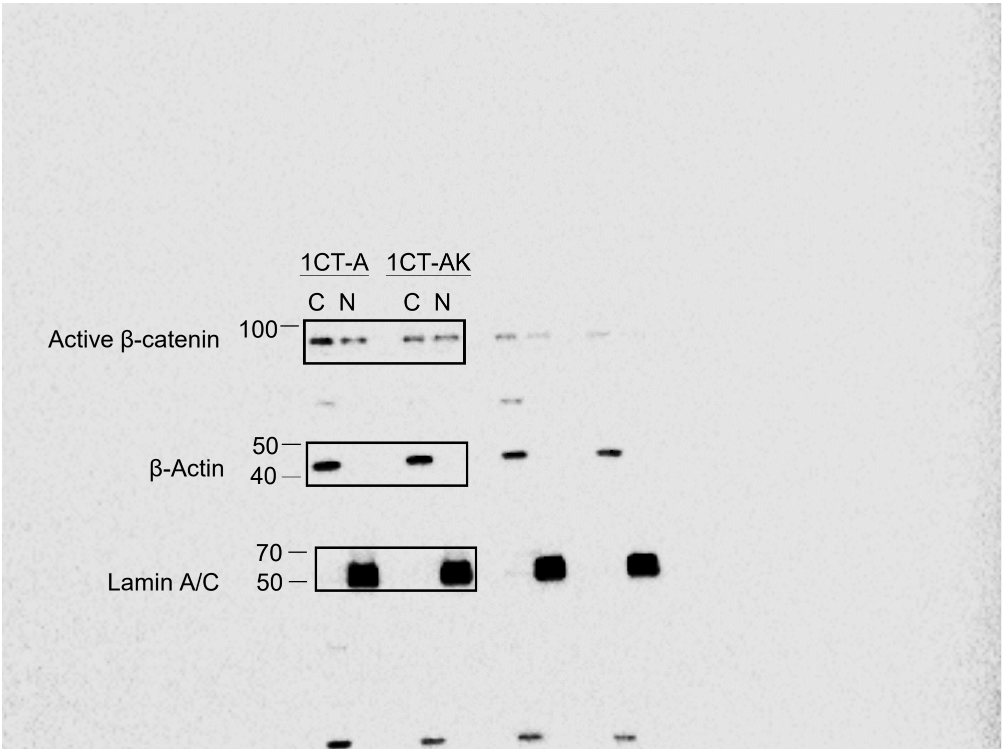

1CT-A
1CT-AK
C
N
C
N
Active β-catenin
100
β-Actin
50
40
Lamin A/C
70
50

Figure S7B

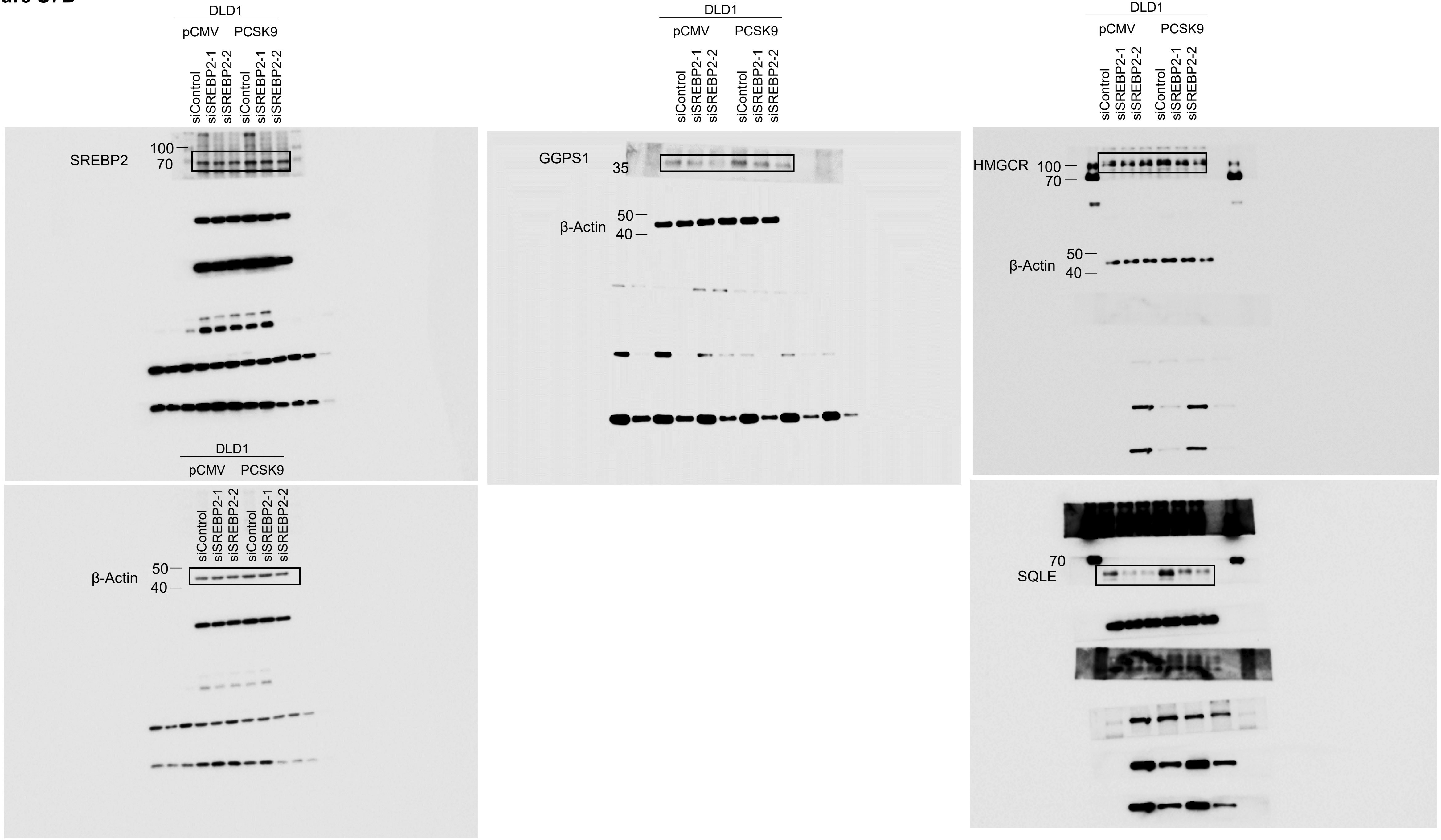
DLD1
pCMV
PCSK9
siControl
siSREBP2-1
siSREBP2-2
siControl
siSREBP2-1
siSREBP2-2
SREBP2
100
70
β-Actin
50
40
GGPS1
35
β-Actin
50
40
HMGCR
100
70
β-Actin
50
40
SQLE
70

Figure S7C

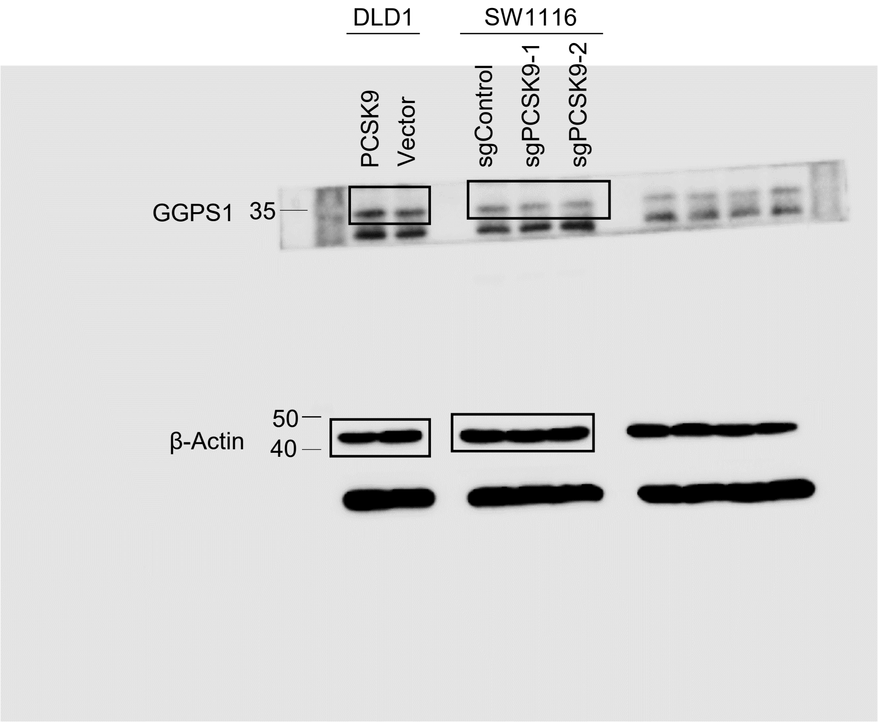
DLD1
SW1116
PCSK9
Vector
sgControl
sgPCSK9-1
sgPCSK9-2
GGPS1
35
β-Actin
50
40

Figure S8A

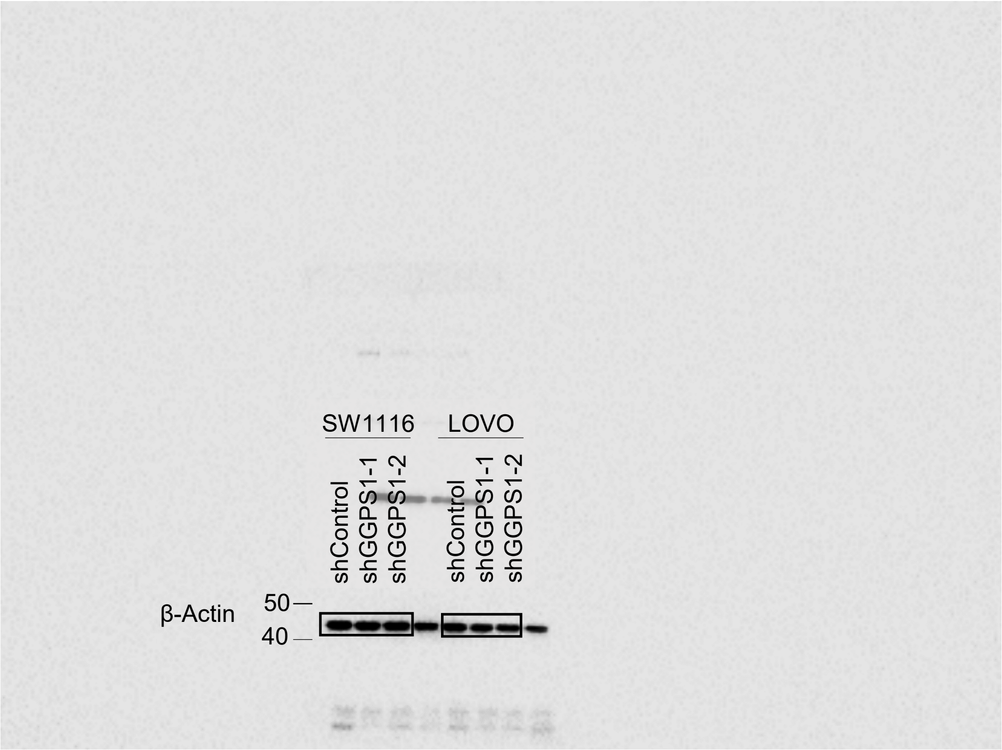
SW1116
LOVO
shControl
shGGPS1-1
shGGPS1-2
shControl
shGGPS1-1
shGGPS1-2
β-Actin
50
40

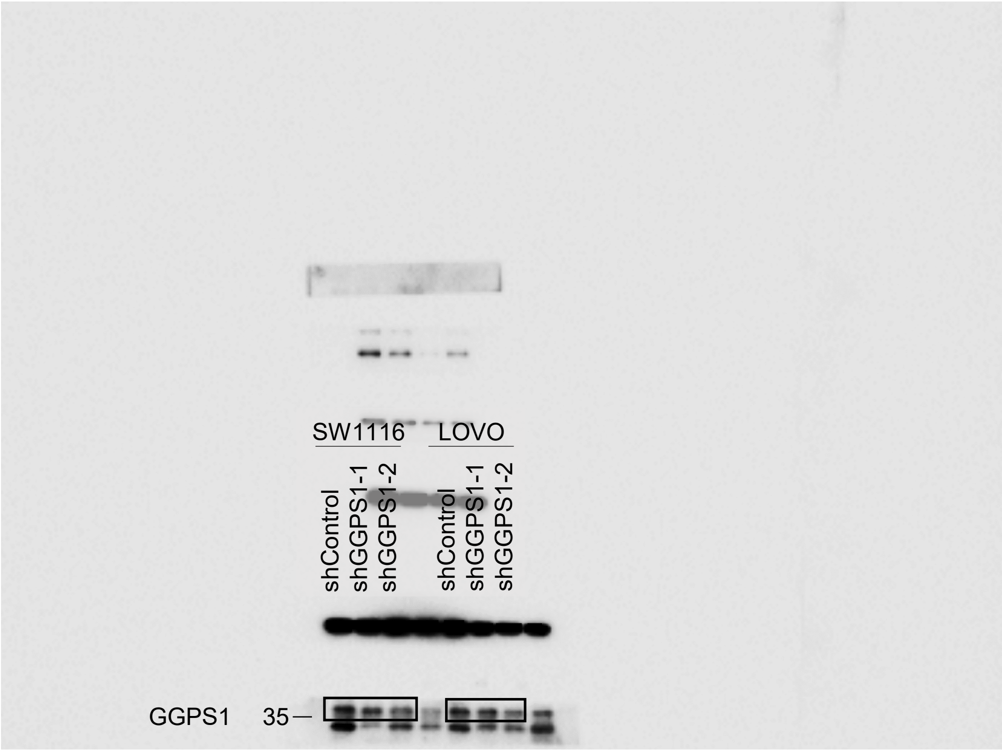
SW1116
LOVO
shControl
shGGPS1-1
shGGPS1-2
shControl
shGGPS1-1
shGGPS1-2
GGPS1
35

Figure S9A

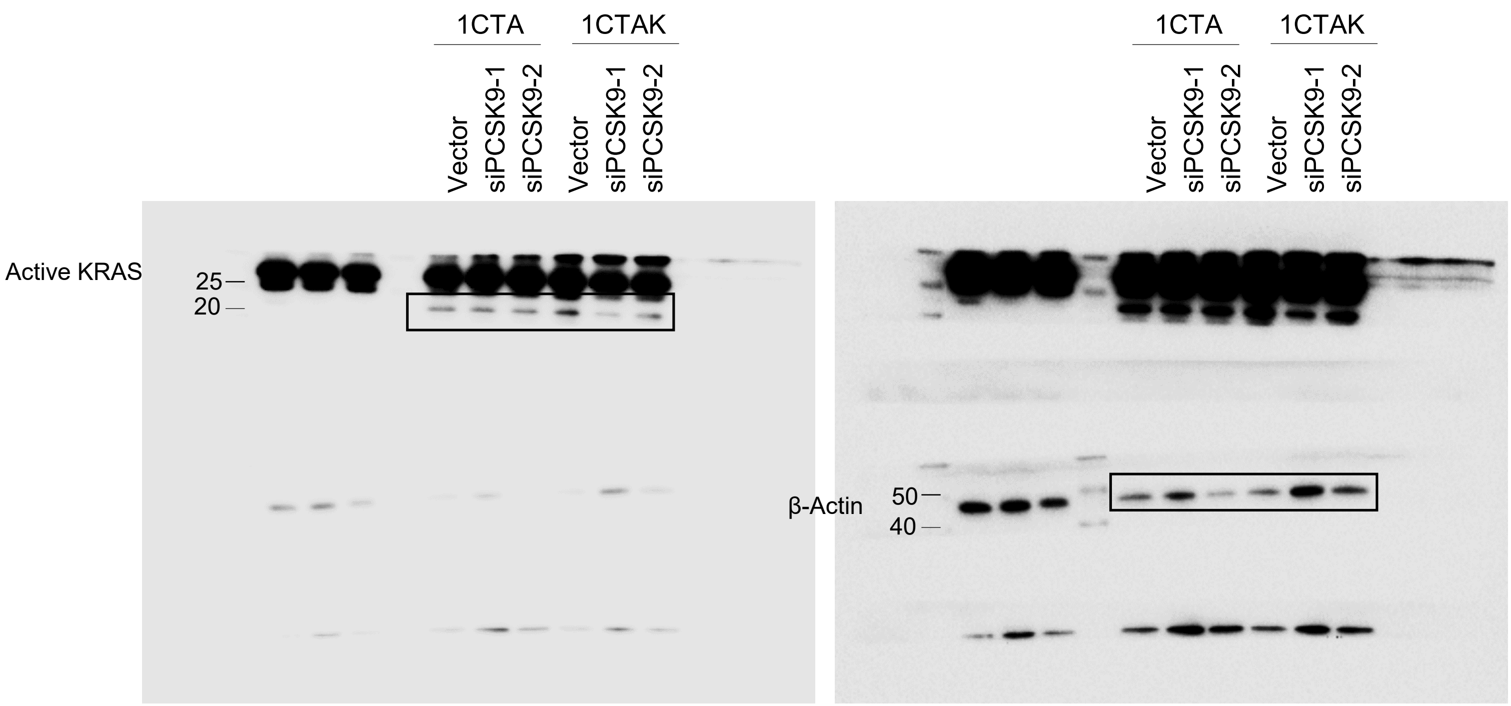
1CTA
1CTAK
Vector
siPCSK9-1
siPCSK9-2
Vector
siPCSK9-1
siPCSK9-2
Active KRAS
25
20
1CTA
1CTAK
Vector
siPCSK9-1
siPCSK9-2
Vector
siPCSK9-1
siPCSK9-2
β-Actin
50
40

Figure S9B

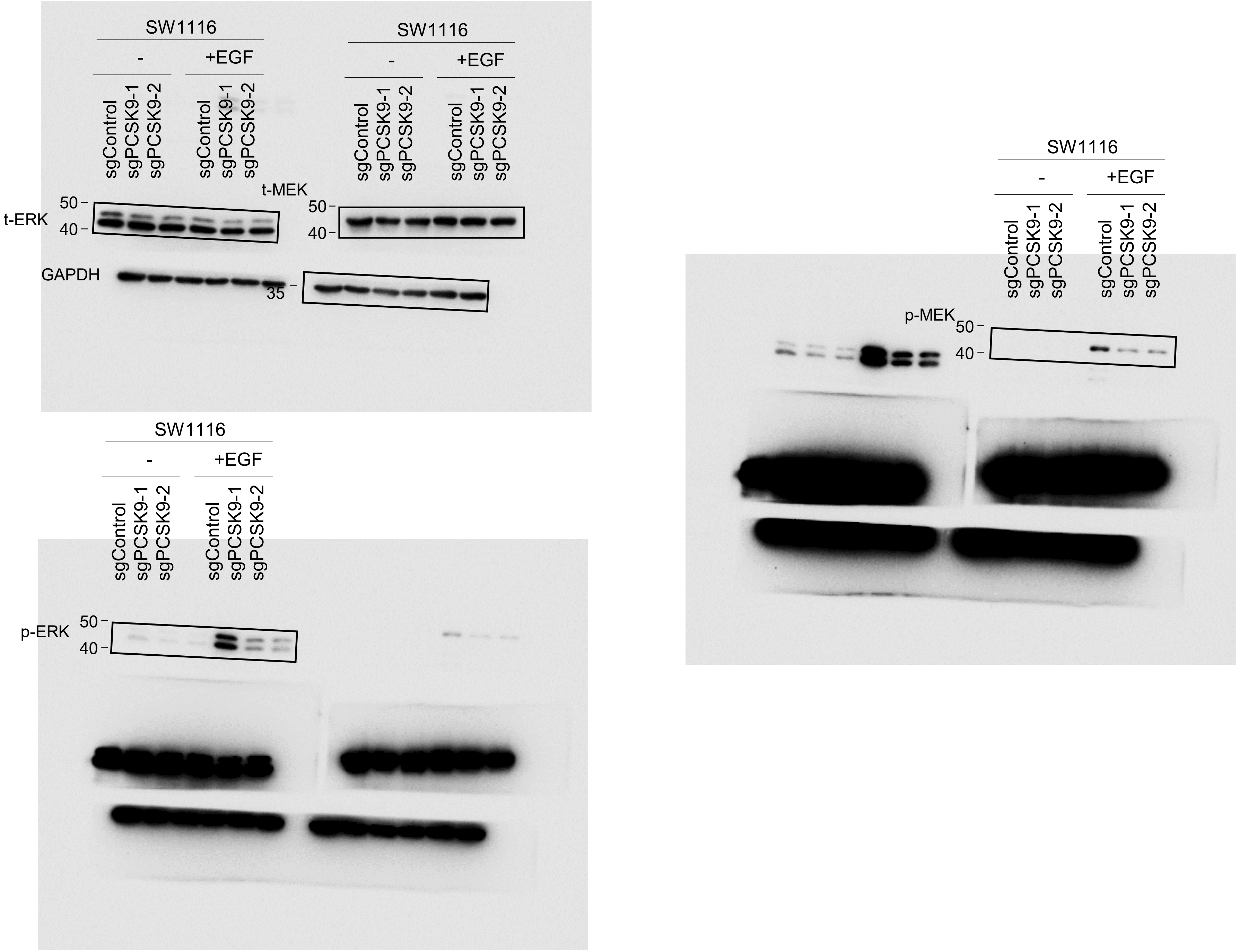
SW1116
-
+EGF
sgControl
sgPCSK9-1
sgPCSK9-2
t-ERK
50
40
t-MEK
GAPDH
35
p-ERK
p-MEK

Figure S10 – Upper panel

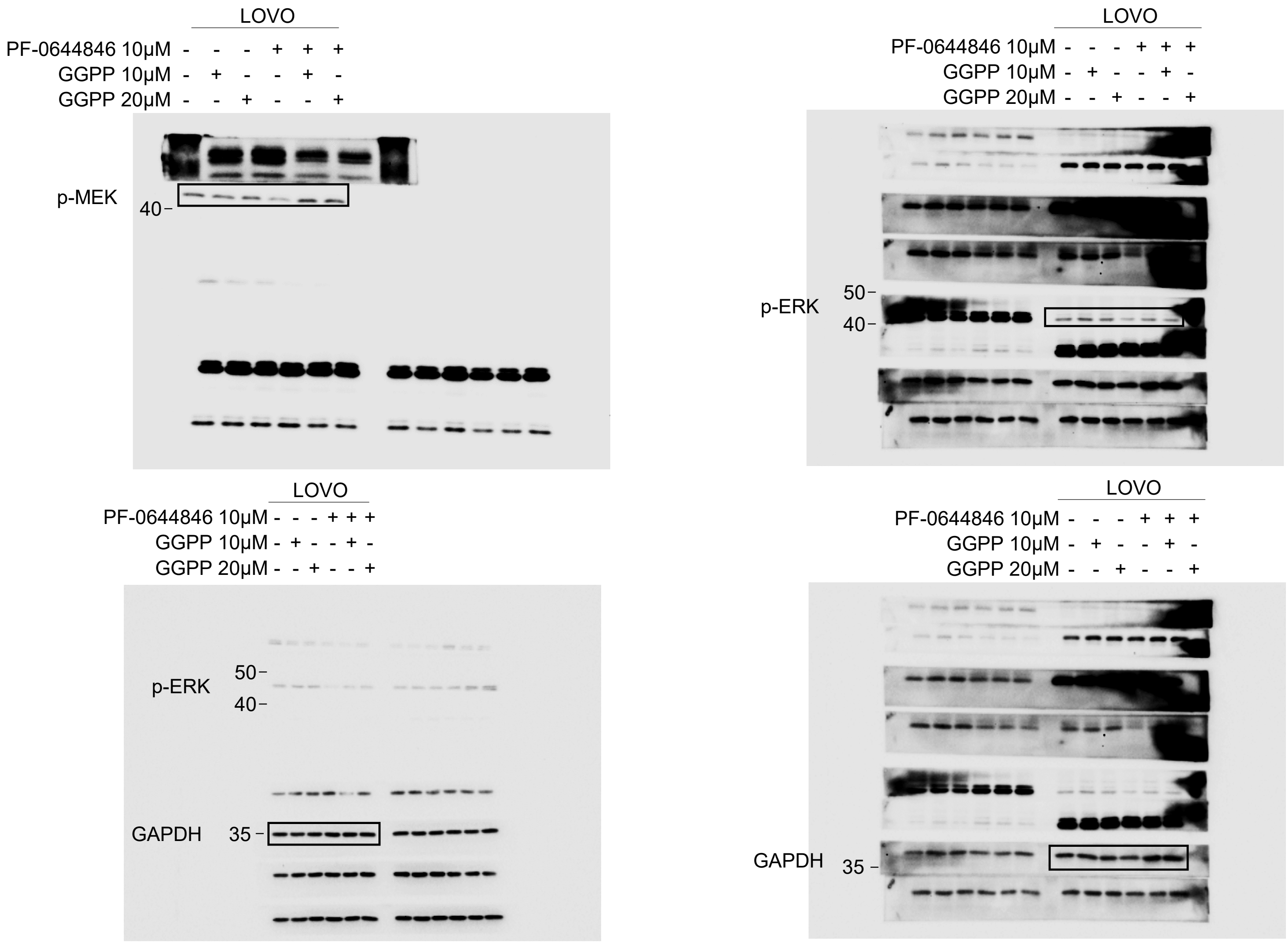

Figure S10 – Lower panel

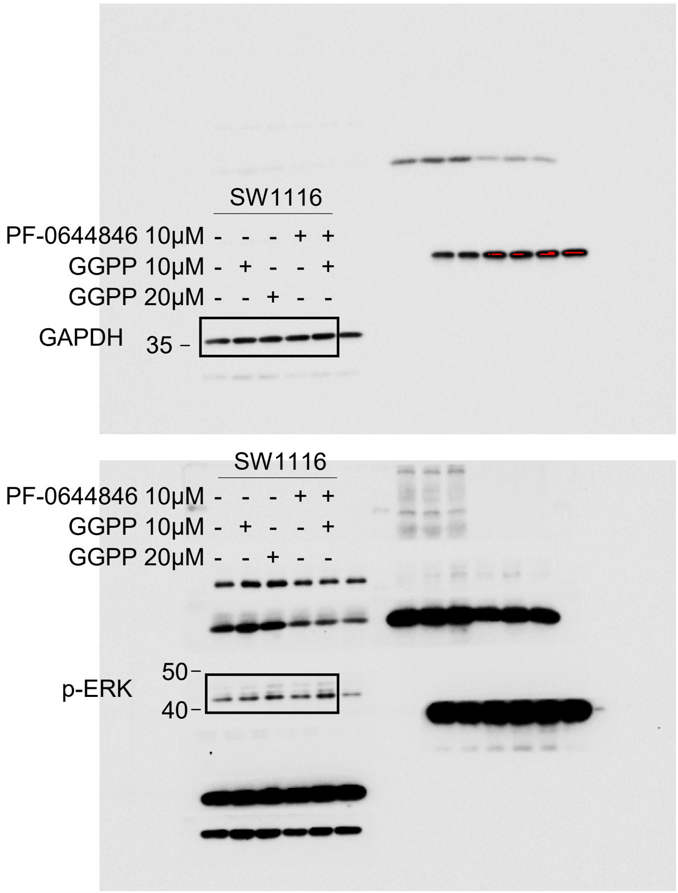

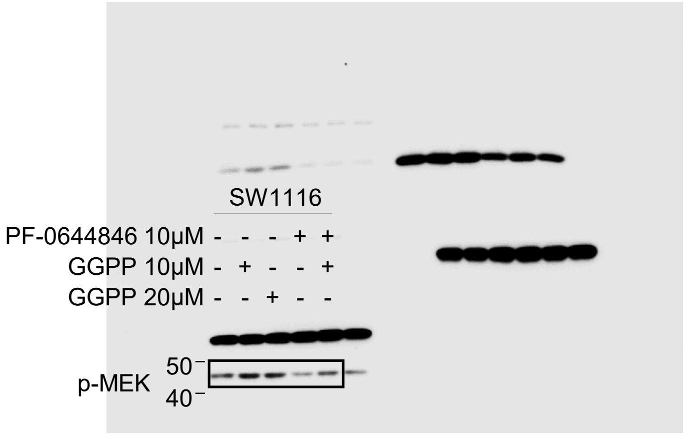

Statistical analysis for Figure 5E, 6E and 6G

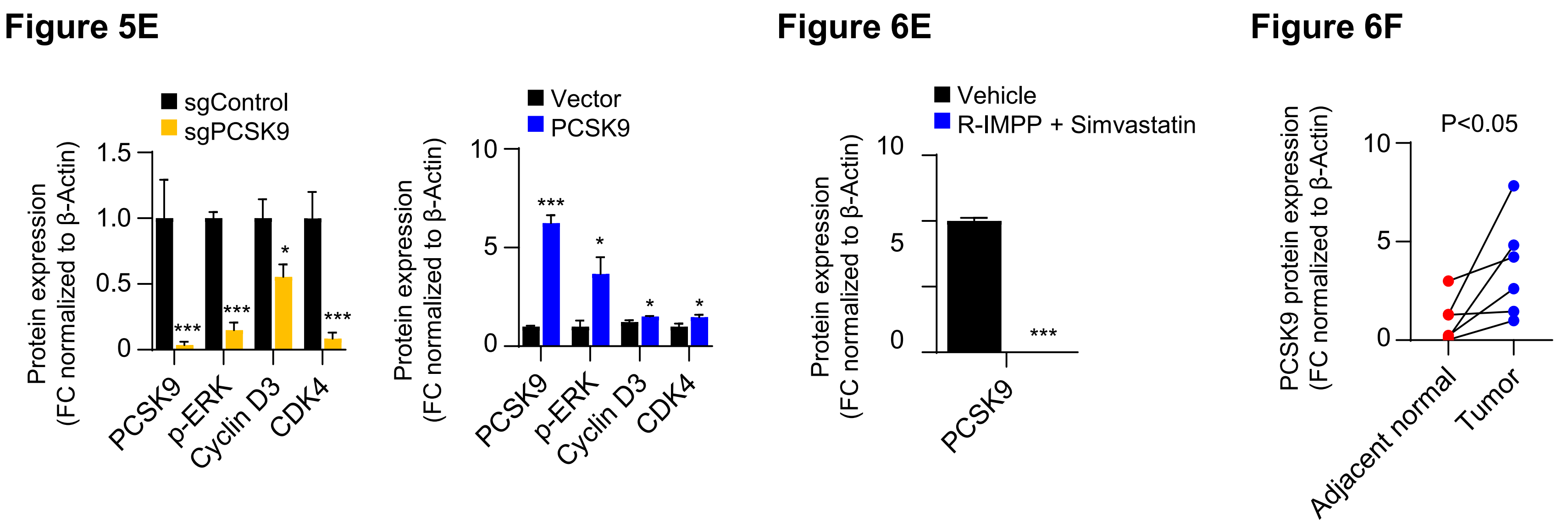

Supplement: Supplementary file 4 — Source Data [file 41467_2022_31663_MOESM4_ESM.zip › Source Data-uncropped blots and gels.pdf]
